# Design, synthesis, computational study and cytotoxic evaluation of some new quinazoline derivatives containing pyrimidine moiety

**DOI:** 10.1038/s41598-023-41530-6

**Published:** 2023-09-02

**Authors:** Somayeh Zare, Leila Emami, Zahra Faghih, Farshid Zargari, Zeinab Faghih, Soghra Khabnadideh

**Affiliations:** 1 School of Pharmacy, Shiraz University of Medical Sciences, Shiraz, Iran; 2 Pharmaceutical Sciences Research Center, Shiraz University of Medical Sciences, Shiraz, Iran; 3 Medical School, Shiraz Institute for Cancer Research, Shiraz University of Medical Sciences, Shiraz, Iran; 4 Pharmacology Research Center, Zahedan University of Medical Sciences, Zahedan, Iran; 5 Department of Chemistry, Faculty of Science, University of Sistan and Baluchestan (USB), Zahedan, Iran

**Keywords:** Biological techniques, Cancer, Computational biology and bioinformatics, Drug discovery

## Abstract

Quinazoline derivatives, as an important category of heterocyclic compounds, have received much attention for the design and development of new drugs due to their various pharmacological properties. Besides, there is a great deal of evidence showing pyrimidine analogs as anticancer agents. Thus, in the present study, for the design of new target compounds with cytotoxic activity, we focused on various quinazolinone and pyrimidine hybrids. A new series of quinazoline-pyrimidine hybrid derivatives (6a-6n) have been designed and synthesized as novel antiproliferative agents. All the synthesized compounds characterized based on their IR, NMR and Mass spectroscopic data. Antiproliferative activities of the new compounds were evaluated against three human cancer cell lines (MCF-7, A549, SW-480). The compounds were found to have appropriate potential with IC_50_ values ranging from 2.3 ± 5.91 to 176.5 ± 0.7 μM against the tested cell lines. Compound 6n exerted the highest antiproliferative activity with IC_50_ values of 5.9 ± 1.69 μM, 2.3 ± 5.91 μM and 5.65 ± 2.33 μM against A549, SW-480 and MCF-7 respectively. The results indicated that 6n could induce apoptosis in A549 cell line in a dose dependent manner and arrest in the S phase of cell cycle. Docking studies were also done to investigate the detailed binding pattern of the synthesized compounds against EGFR. Furthermore, molecular dynamic simulation and binding free energy calculation have been done to rescore initial docking pose of the synthesized compounds using ensemble-based MMGB/PBSA free energy method. According to the results, free energy calculation confirmed biological activity of compounds and also, Arg 817 and Lys 721 residues had the pivotal role in the high potency of 6n. Finally, the drug likeness and in silico ADME study were also predicted.

## Introduction

**Figure 1:**
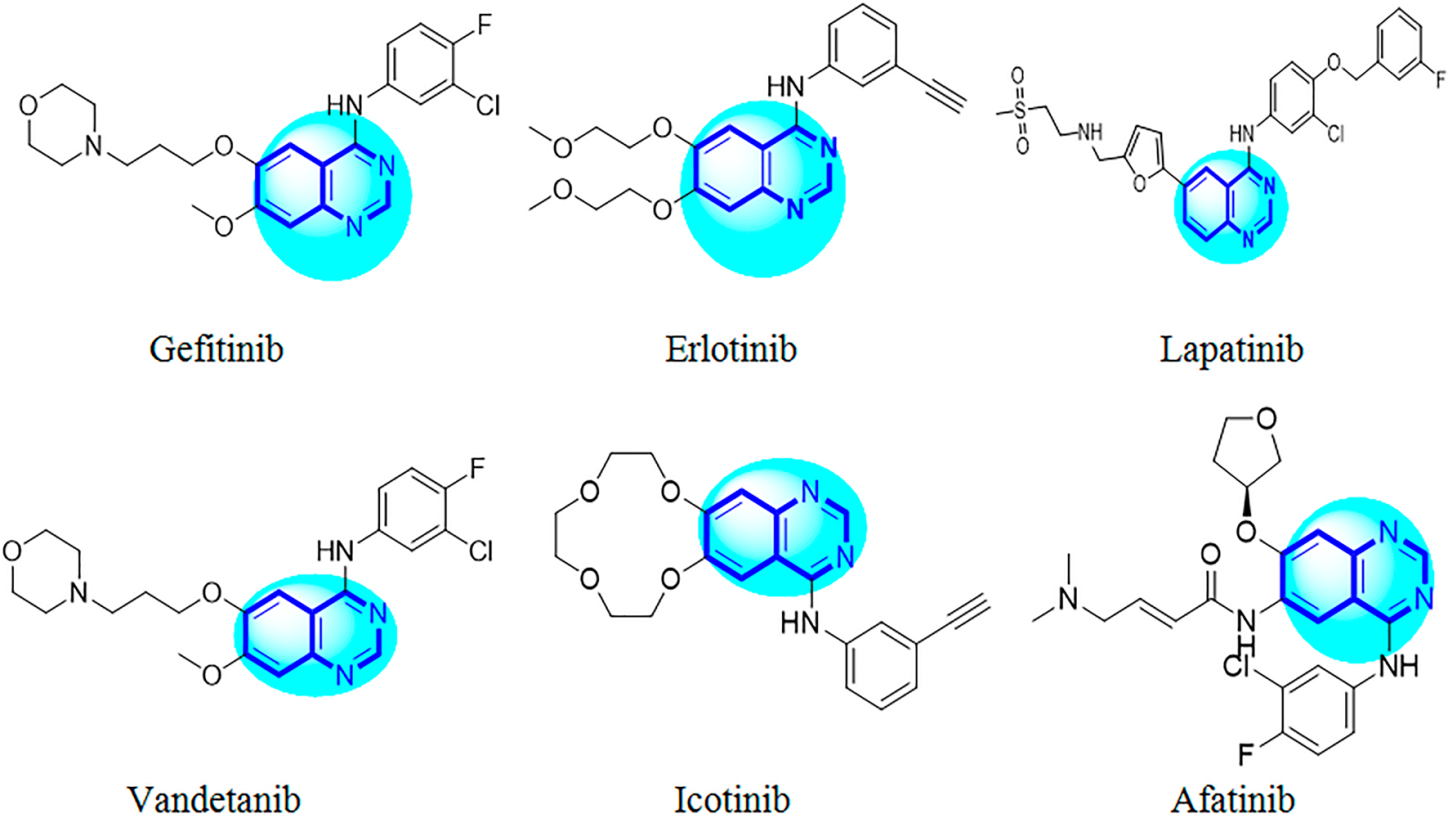
Clinically approved quinazoline scaffolds as EGFR inhibitors.

Cancer, one of the principal causes of death, is a multifactorial disease that comprises numerous genetic defects and is characterized by abnormal growth of cells^1, 2^. According to the World Health Organization (WHO) nearly 10 million cancer deaths occurred in 2022 in worldwide. Chemotherapy is considered a basis in the management of many types of cancer, however, its effectiveness in the curing of cancer is partly troubled by drug resistances^3^. However for most types of disseminated cancers, no effective treatment is available, and development of new active chemotherapeutic agents is urgently needed^4^. More than 90% of the novel drugs bear heterocycle rings in their structure and among them, nitrogen containing heterocyclic compounds display more notable pharmaceutical effect than non-nitrogen compounds^5^. Quinazoline scaffolds represent an important class of biologically active nitrogen heterocyclic compounds and a variety of marketed drugs are based on these moieties^6^ with broad spectrum of pharmacological activities and minimum side effects^7^. Pharmacological activities of quinazoline and its related scaffolds include anti-cancer, antifungal, anti-tumor, anti-malaria, anticonvulsant, anti-microbial, anti-inflammatory and antihyperlipidemic activities^8–12^. Quinazoline compounds have been also shown to inhibit tyrosine kinase activities, and accordingly are useful to prevent tumor growth^13^. Quinazoline derivatives are one of the largest chemical groups reported as potent Epidermal Growth Factor Receptor (EGFR) inhibitors. EGFR, among different receptors and their associated signaling proteins, widely play a critical role in tumor genesis^14, 15^. Evidences for the role of EGFR in inhibition and pathogenesis of various cancers have led to the development of agents that target this receptor. Activation of the EGFR signaling pathway in cancer cells has been linked with increased cell proliferation, angiogenesis and metastasis^16^. In recent years, several quinazoline scaffolds such as Gefitinib (2003), Erlotinib (2004), Lapatinib (2010), Vandetanib and Icotinib (2011), Afatinib (2013) clinically approved as EGFR inhibitors (Fig. 1)^13^.

**Figure 2:**
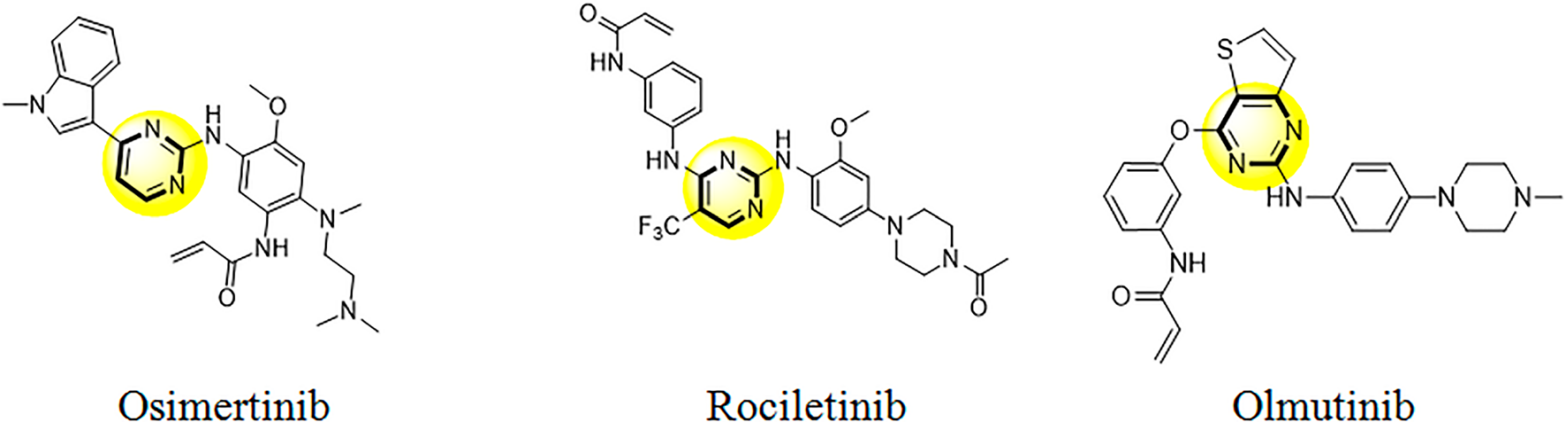
Clinically approved pyrimidine scaffolds as EGFR inhibitors.

In addition, literature review has discovered that many effective EGFR tyrosine kinase inhibitors were derived from pyrimidine nuclei i.e. Osimertinib^17^, Rociletinib^18^ and Olmutinib (Fig. 2)^19^. Recently, efforts have been made to synthesize novel hybrid derivatives of quinazoline and pyrimidine^20^. Hybrid molecules with two or more pharmacophores usually have a better potential to reduce side effects and overcome drug resistance. There are many hybrid molecules in different phases of clinical trials for treatment of various diseases. This suggests that hybridization is a useful strategy for development of the novel drugs^21^. Notably, pyrimidine, quinazoline and their analogues have been reported to have beneficial effects in varieties of cancer^22^. In our previous study we reported a series of quinazolinone–pyrimidine hybrids as dipeptidyl peptidase-4 (DPP4) inhibitors^23^. Here, we have focused on design and synthesis of new series of quinazolinone–pyrimidine hybrids as EGFR inhibitors. In this regard 14 hybrids (6a-6n) were synthesized and characterized by different spectroscopic methods. Biological evaluations of the synthesized compounds were tested against three cancerous cell lines (A549, SW-480 and MCF-7). Molecular docking study was also performed to explore the possible mechanisms of the newly synthesized compounds. Finally, we used ensemble-based MMGB/PBSA free energy method to simulate molecular dynamic and calculate binding free energies to rescore initial docking pose of the synthesized compounds.

## Results and discussion

### Design approach

**Figure 3:**
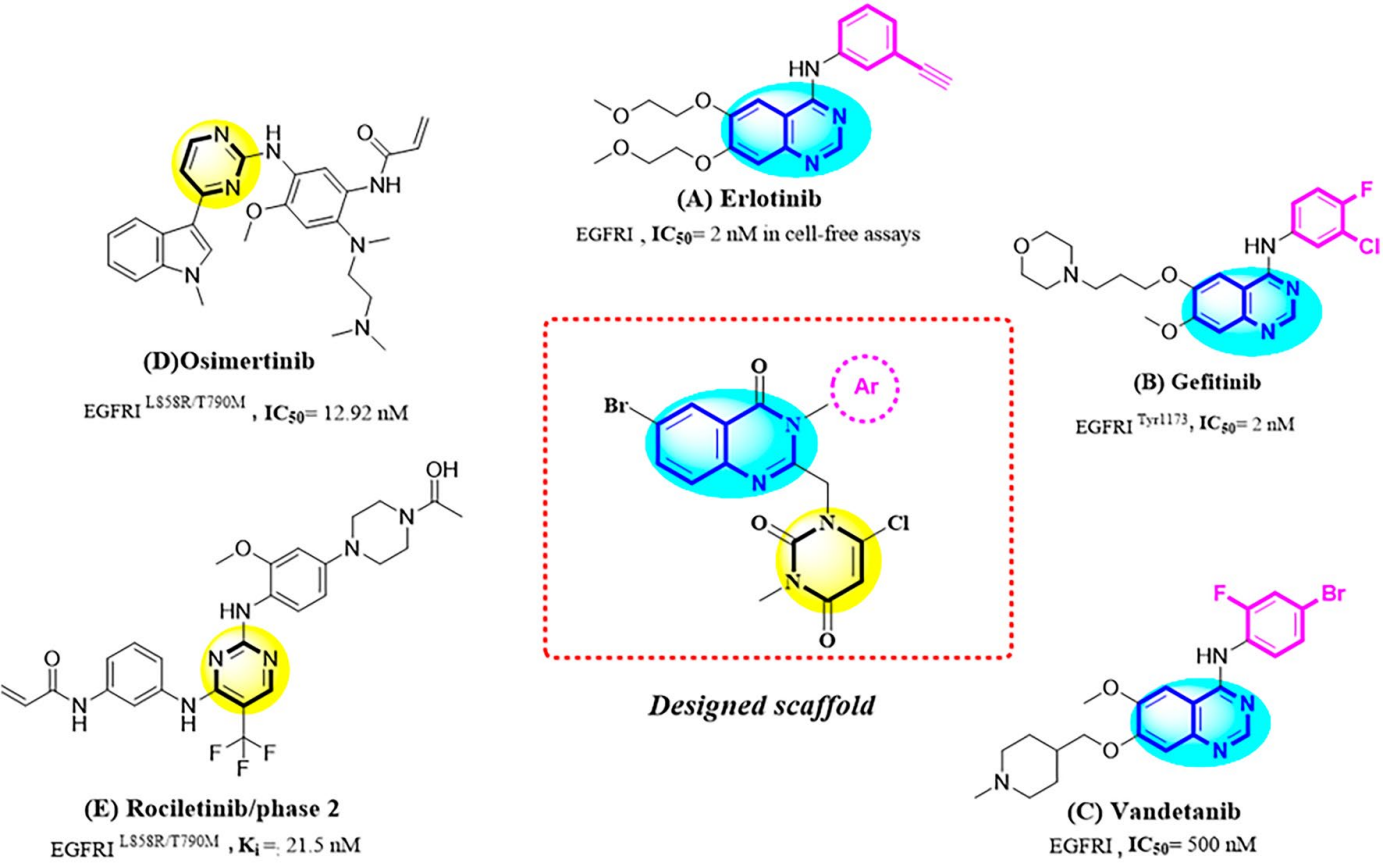
Design of novel quinazoline-pyrimidine derivatives.

EGFR tyrosine kinase is considered as one of the most effective clinically targets for treatment of non-small-cell lung cancer (NSCLC)^24, 25^. Tyrosine kinase inhibitors (TKIs) can be classified into reversible and irreversible inhibitors. These inhibitors bind reversibly and competitively to the ATP-binding site of the tyrosine kinase domain of the intracellular EGFR and prevent its phosphorylation and downstream pathways^26^. Erlotinib and Gefitinib were approved as selective EGFR tyrosine kinase inhibitors by Food and Drug Administration (FDA) for treatment of NSCLC cancer patients^27, 28^. Pyrimidine derivatives such as Osimertinib show significant activity against EGFR with an IC_50_ value of 12.92 nM^29^. Osimertinib was approved by FDA in 2015 and can be used as first-line treatment for advanced NSCLCs according to the current European Society of Medical Oncology (ESMO) recommendation^30^. In this study, we used a combinatorial pharmacophore approach to design novel antiproliferative compounds as EGFR inhibitors. To achieve synergistic cytotoxic effects, the pyrimidine ring was attached to the 6-boromoquinazoline backbone having different benzyl substitution at *N*-3 position with different electronic profiles (Fig. 3).

### Synthesis and characterization of 6-boromo-quinazolinone–pyrimidine hybrids (6a–6n)

The synthesis route of 6-boromo-quinazolinone–pyrimidine hybrids (6a-6n) is shown in Fig. 15. First, a mixture of anthranilic acid (1) and *N*-bromo succinimide in acetonitrile was used to prepare 5-boromoantranilic acid (2). Then 5-boromoantranilic acid was reacted with chloroacetyl chloride under the basic conditions (DIPEA) in DCM at room temperature for 2 h to synthesize 6-bromo benzoxazine-4-one (3). Then, various substituted anilines (4a-4n) were reacted with *3 *via nucleophilic attack under acidic conditions in acetonitrile. Finally, 6-boromoquinazoline derivatives (5a-5n) were refluxed with 6-chloro-3-methyl-uracil at 80 °C in the presence of DIPEA to obtain the final compounds (6a-6n) in good yield. The final compounds were purified by plate chromatography using chloroform/*n-*hexane as eluents (4:1) (Table 1). Chemical structures of the final compounds were confirmed by IR, ^1^H-NMR, ^13^C-NMR, and Mass spectroscopies. In IR analysis, the stretching frequency of carbonyl bonds were observed at 1674–1719 cm^−1^. In ^1^H NMR spectra of compounds 6a-6n compounds a signal at 4.72–5.24 ppm associated with the CH_2_ protons which are between 6-chloro-3-methyl-uracil and quinazolinone. The singlet peak at 8.29–8.41 ppm indicated the presence of a proton at C5 position of the quinazoline moiety. A single signal at 5.98–6.14 ppm corresponds to the proton of uracil. In the ^13^C NMR spectra, the chemical shift at 157.82–164.31 ppm and 47.57–66.4 ppm related to the carbonyl groups and CH_2_ moieties, respectively.

**Table 1:** Chemical structures and physical properties of the synthesized compounds (6a-6n).


Compound	R	M.P. (°C)	Yield (%)	B.E (kcal/mol)	Entry	R	M.P. (°C)	Yield (%)	B.E (kcal/mol)
6a	H	246–248	77	–8.2	6h	4-Br	232–233	73	–8.7
6b	3-CN	255–257	73	–8.6	6i	4-Me	224–225	71	–8.9
6c	3-F	243–244	76	–8.6	6j	4-OMe	225–226	70	–8.7
6d	3-Cl	259–260	78	–9.5	6k	4-phenoxy	201–203	72	–9.8
6e	3-Br	251–252	74	–8.7	6l	2,4-diOMe	247–248	73	–8.1
6f.	4-F	240–242	81	–8.6	6m	3,4-diM	252–253	84	–9
6g	4-Cl	225–226	82	–8.6	6n	3Cl-4F	257–259	79	–9.14

### In vitro cytotoxicity screening

Antiproliferative activities of all the synthesized compounds (6a-6n) were investigated against three human cancerous cell lines, A549 (human lung adenocarcinoma), SW-480 (colorectal cancer), and MCF-7 (human breast cancer). The best cytotoxic effect was obtained for lung cancer (A549) followed by colorectal cancer (SW-480). Compound 6n with two electron withdrawing atoms (Cl and F) at positions 3 and 4 of the phenyl ring showed good activity with IC_50_ = 5.9 ± 1.7 µM, 2.3 ± 0.91 µM and 5.65 ± 2.33 µM compared to Cisplatin with IC_50_ = 15.37 µM, 16.1 µM and 3.2 against A549, SW-480 and MCF-7 cell lines respectively (Table 2).

**Table 2:** Antiproliferative activities of the synthesized compounds (6a-6n) toward three tested cell lines.

Compound	Cytotoxicity (IC_50_ ± SD) µM			
	A549	SW-480	MCF-7	MRC-5
6a	136 ± 4.24	> 200	> 200	ND*
6b	56 ± 2.82	62.5 ± 0.77	96 ± 2.82	ND
6c	51.8 ± 2.1	47.5 ± 2.05	67 ± 1.41	ND
6d	75 ± 2.61	122 ± 2.82	> 200	ND
6e	72.5 ± 3.46	120 ± 5.65	176.5 ± 0.7	ND
6f.	26.5 ± 3.53	23.3 ± 2.4	21.9 ± 5.79	ND
6g	17 ± 3.82	20.9 ± 4.31	19 ± 3.82	ND
6h	8.58 ± 0.63	4.5 ± 1.4	12.1 ± 1.55	159.5 ± 12.02
6i	29 ± 1.41	26.4 ± .3.67	22.5 ± 4.92	ND
6j	32.5 ± 1.26	26.5 ± 2.37	65.4 ± 2.26	ND
6k	8.7 ± 1.76	10.35 ± 3.33	17.2 ± 3.95	312.4 ± 14.9
6l	83.5 ± 3.81	> 200	> 200	ND
6m	126 ± 1.41	> 200	> 200	ND
6n	5.9 ± 1.7	2.3 ± 0.91	5.65 ± 2.33	168 ± 8.48
Cisplatin	15.37 ± 1.61	16.1 ± 1.87	3.2 ± 0.8	13.5 ± 1.5

**Figure 4:**
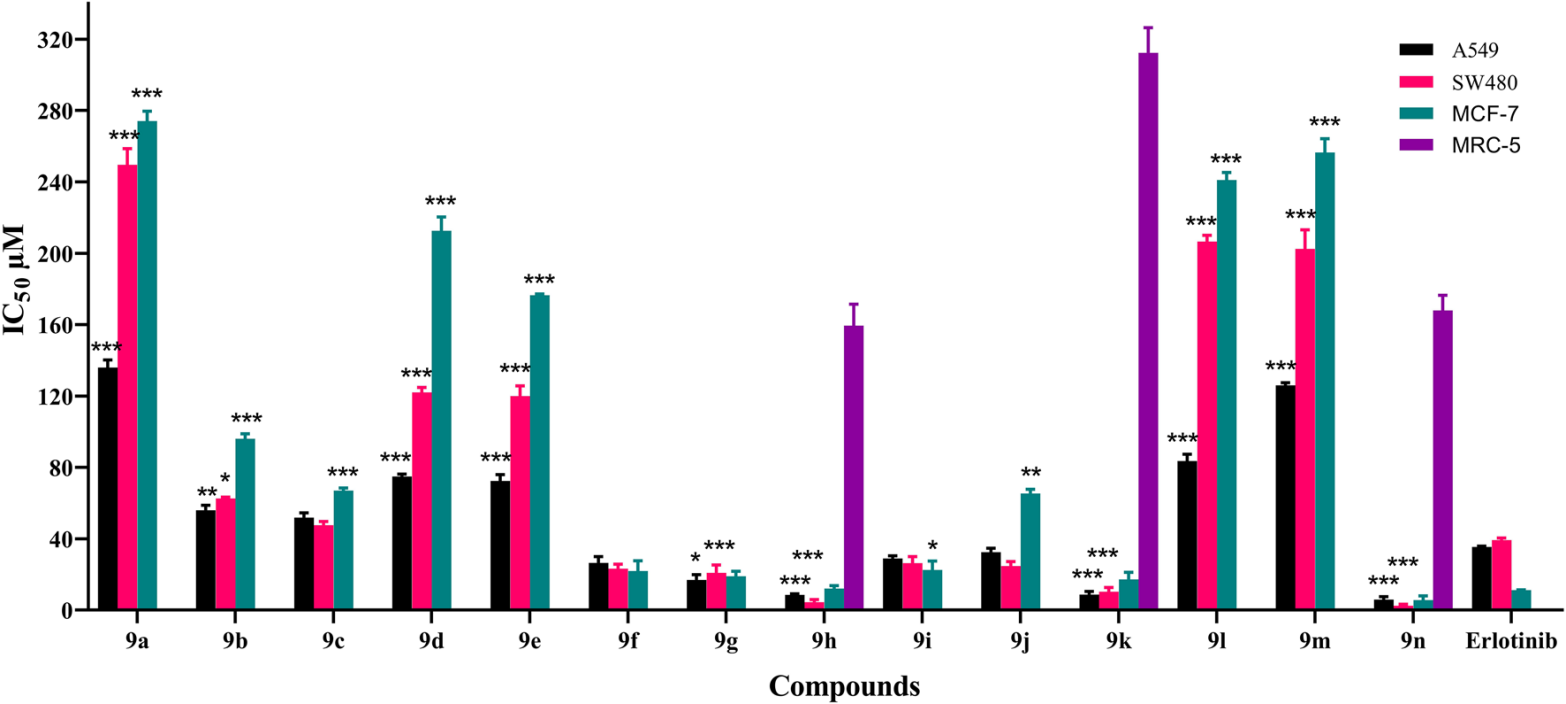
Cytotoxic effects of the synthesized compounds (6a-6n) on three cancer and normal tested cell lines (*p < 0.05, **p < 0.01, ***p < 0.001).

The synthesized compounds could be classified in two categories as mono-substituted (6a-6k) and di-substituted (6l-6n). In the mono substituted category compounds 6h and 6k with bromine and phenoxy groups at *para* position of the phenyl ring displayed significant antiproliferative effects (Table 2). The presence of electron-withdrawing groups (halogen atoms) at *para* position of the phenyl ring in compounds 6f., 6g and 6h seems to cause these compounds more active than compounds 6i and 6j with electron-donating groups in order of Br > Cl > F. Changing the halogen substitutions from *para* to *meta* position resulted in 1.95–8.44 folds’ decrease in activity. In the di-substituted groups, all compounds had low cytotoxic activity in the range of > 80 µM except 6n, which is consistent with the mono-substituted compounds. The unsubstituted compound 6a was less active compared to all other substituted compounds. Structure activity relationship (SAR) studies showed that compounds containing one or two halogen substitutions on the phenyl ring, have significant antiproliferative activity (Fig. 4). On the other hand, the presence of OMe and Me groups reduced the efficacy. In addition, the presence of substitution at *para* position was more effective than in the *meta* position. Having bulky substitution (4-phenoxy) on the phenyl ring *(*6k*)* also increased the inhibitory activity. In addition, to evaluate the selectivity activity of compounds between normal and cancerous cell lines, the cytotoxic activity on the normal lung cell line (MRC-5) was applied for three potent compounds (6h, 6k and 6n). The results showed a desire selectivity (Table 2).

### Apoptosis detection

**Figure 5:**
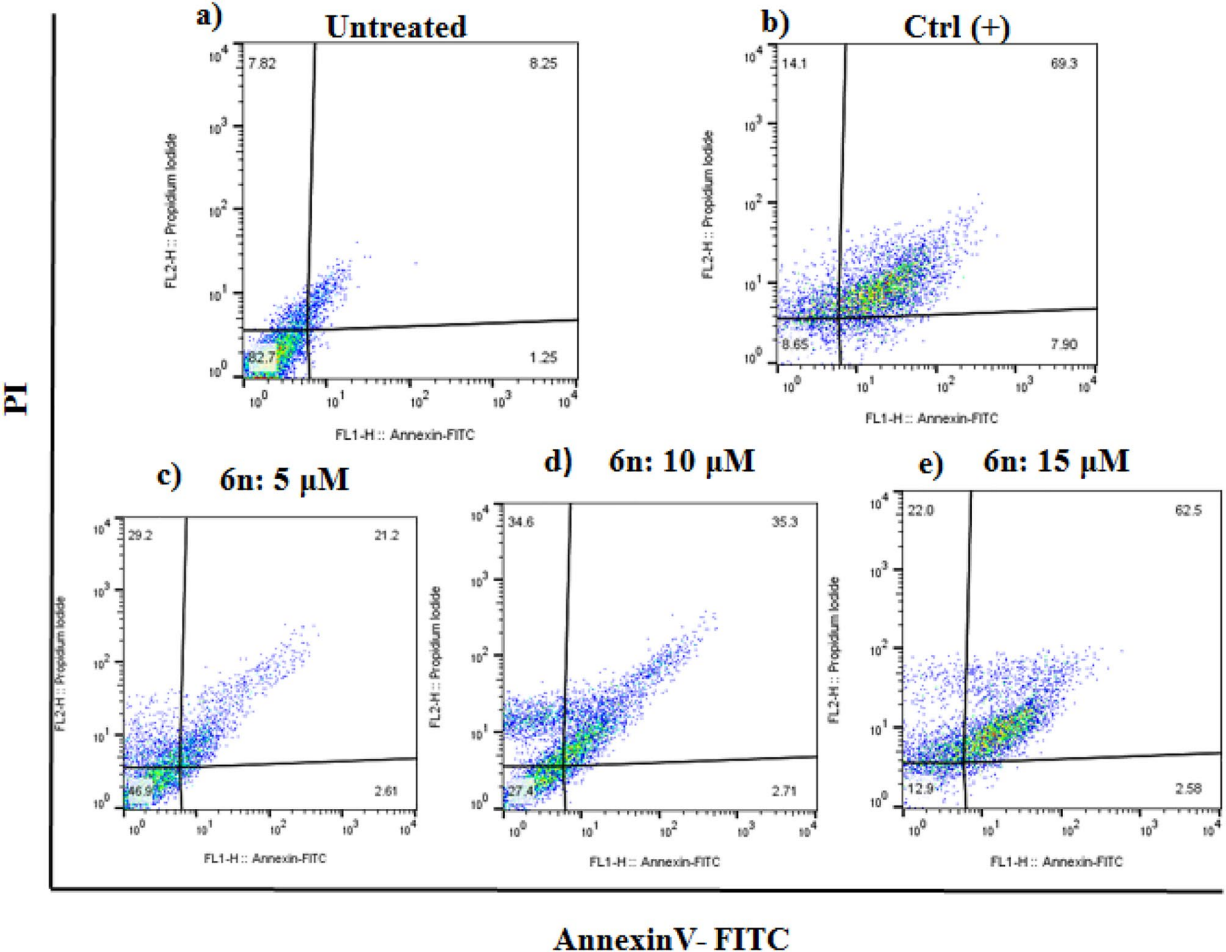
Flow cytometric analysis of apoptotic effect on A549 cell line after 72 h for *6n*, (**a**) Untreated cells, (**b**) Ctrl ( +) heated at 56 °C, (**c**) 6n (5 µM), (**d**) 6n (10 µM), (**e**) *6n* (15 µM).

Cell death mechanism of compound 6n as promising compound was investigated via Annexin V-Propodium Iodide (PI) double staining technique. As could be seen, using this technique, four staining patterns could be detected: viable cells Q4: (Av^neg^/PI^neg^), early apoptotic cells Q3: (Av^pos^/PI^neg^), late apoptotic cells Q2: (Av^pos^/PI^pos^) and necrotic cells Q1: (Av^neg^/PI^pos^). Annexin V was applied to detect the phosphatidyl serin, which moved to outer membrane during apoptosis, and PI was used to distinguish live and dead cells. The apoptotic activity of 6n on A549 cell line in three different concentrations is shown in Fig. 5. The results showed that 6n significantly induced apoptotic death in the dose dependent manner from (2.61% early apoptosis and 21.2% for late apoptosis in 5 µM concentration), to 33.26% and 65.08% apoptotic death in 10 µM and 15 µM concentration.

### Cell cycle analysis

**Figure 6:**
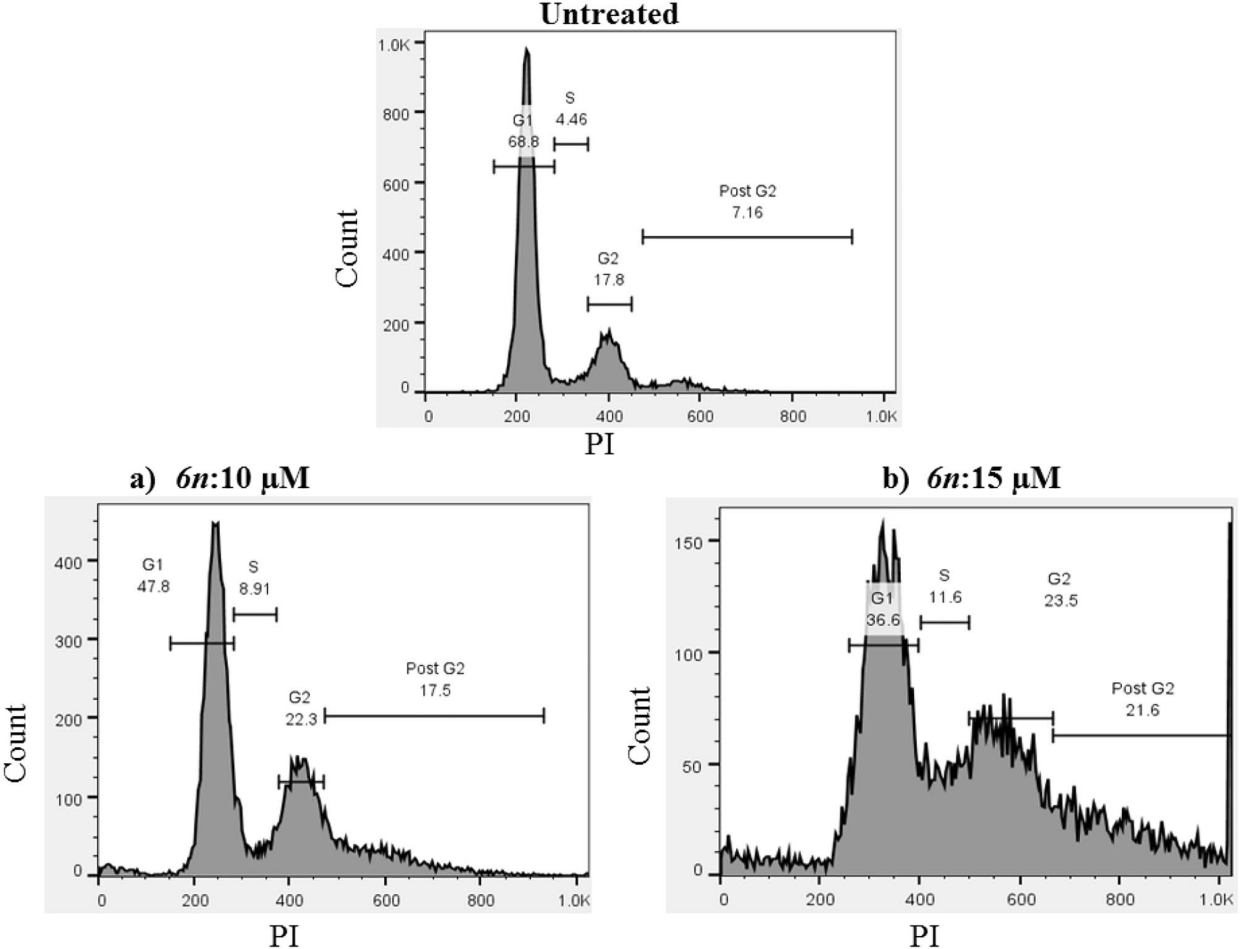
The effect of different concentrations of compound **6n** (**a**) 10 μM and (**b**)15 μM on the cell cycle of A549 cell line after 72 h of treatment.

To investigate the effect of compound 6n on the cell cycle distribution of A549 cell line, a flow cytometric based method was used. For this purpose, A549 cells were treated with two concentrations of 6n (10 and 15 μM) for 72 h (Fig. 6). As could been seen, the A549 cells exposed to compound 6n showed accumulation in the S-phase with a percentage of 8.91% and 11.6% following treatment with 10 and 15 μM, respectively, compared to untreated cells (4.46%). A slight decrease in the G2/M population was also observed. These results collectivel suggest that compound 6n could induce arrest at S-phase.

### Molecular docking study

**Figure 7:**
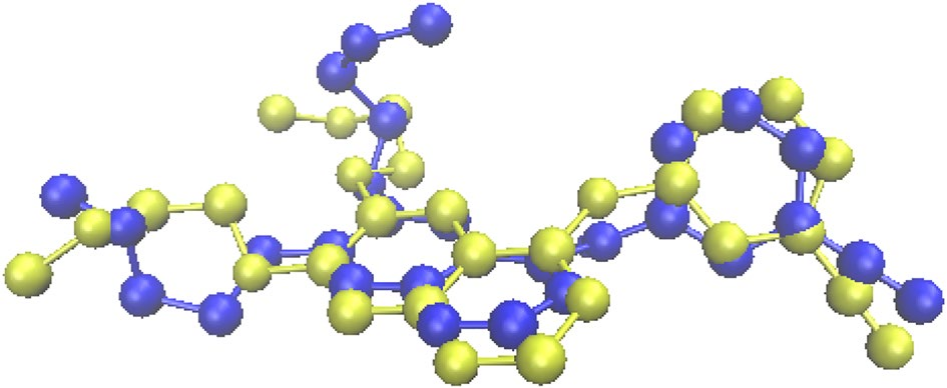
Erlotinib in two different conformations at the EGFR active site (PDB: 1M17): The redocked model was indicated by a yellow color, and the blue color illustrated the crystal orientation.

Molecular docking simulations based on the 3D structure of EGFR (PDB code: 1M17) was performed using Auto Dock Vina software for the synthesized compounds (6a-6n) and Erlotinib as an internal ligand^31^. In Fig. 7, the superimposing of the internal ligand (Erlotinib) before and after the docking operation was displayed. The binding free energies and interaction details of Erlotinib and the synthesized compounds (6a-6n) against EGFR were summarized in Table 3.

**Table 3:** The detailed interactions of the all synthesized compounds on 1M17 using AutoDock Vina.

Entry	Amino Acid and distance (A°)	Ligand involved moiety	Type of interaction
*6a*	Lys 721 (1.96)	C = O moiety	Hydrogen bond
	Asp 831 (3.91), Phe 699 (3.85, 4.60), Pro 853 (5.39)	Quinazoline & pyrimidine moiety	Pi interactions (pi Anion, Pi-Pi Stacked, pi-alkyl)
	Val 702 (4.67), Phe 699 (4.61)	Br	alkyl
	Gly 833, Glu 734, Leu 834, Asp 813, Arg 817	–	Vander waals
*6b*	Arg 817 (3.70), Lys 721 (3.0), Cys 773 (4.13)	CN, C = O & pyrimidine moiety	Hydrogen bond
	Asp 831 (4.10), Asp 776 (4.53), Val 702 (4.65), Lue 820 (5.43), Ala 719 (4.90), Lys 721 )5.24)	Quinazoline & pyrimidine moiety	pi Anion, Pi-Donor ydrogen Bond, Pi-sigma, Pi-Pi Stacked, Pi-Alkyl )
	Ala 719 (4.35), Lys 721 (3.78)	Br	alkyl
	Phe 699, Asp 818, Asp 813, Thr 766, Lue 694	–	Vander waals
*6c*	Leu 694 (3.6), Lys 721 (2.93)	Pyrimidine & C = O moiety	Hydrogen bond
	Asp 831 (4.58), leu 694 (3.95), Phe 699 (4.97), Lys 721 (4.85), Leu 820 (5.37), Ala 719 (5.08), Val 702 (4.22, 4.93)	–	Pi interactions (pi-Anion, , pi-sigma, pi–pi, pi alkyl)
	Met 742 (5.04), Leu 764 (4.42), Lys 721 (4.20)	Br	Alkyl
	Gly 772, Met 769, Gly 695, Gly 697, Thr 766	–	Vander waals
*6d*	Cys 773 (3.67), Gly 772 (2.64), Phe 771 (3.34)	Pyrimidine moiety	Hydrogen bond
	Leu 694 (5.09, 5.16), phe 699 (4.73), Ala 719 (5.25), Leu 820 (4.87)	Quinazoline & phenyl moiety	pi-alkyl, pi-sulfur
	Ala 719 (5.25), Leu 820 (4.77)	phenyl moety	Alkyl
	Gly 695, Val 702, Gln 767, Asp 776, Thr 771, Thr 766, Lue 768	–	Vander waals
*6e*	Lys 721 (3.09), Met 769 (3.41)	C = O & pyrimidine moiety	Hydrogen bond
	Val 702 (4.29, 4.70), Ala 719 (4.39), Lue 694 (5.24), Lue 820 (3.93), Lys 721 (5.05)	Quinazoline & pyrimidine moiety	Pi-Anion, Pi-Alkyl
	Lys 72 (3.93), Lue 764 (4.77)	Br	Alkyl
	Phe 699, Cys 773, Gly 772, Lue 768, Met 742, Thr 766	–	Vander waals
*6f.*	Lys 721 (2.60), Met 769 (3.59)	C = O moiety and CH_3_	Hydrogen bond
	Asp 831 (4.77), Lue 694 (4.25), Lue 820 (5.37), Lys 721 (4.73), Ala 719 (4.73)	Quinazoline & pyrimidine moiety	Pi-Anion, Pi-Alkyl
	Lue 764 (4.55), Lys 721 (3.71)	Br	Alkyl
	Asp 776, Phe 771,Phe 699, Leu 768Gly 772, Gly 695, Gly 697, Thr 766	–	Vander waals
*6g*	Cys 773 (3.09), Lue 820 (366)	Pyrimidine and CH_2_ moiety	Hydrogen bond
	Asp 831 (3.54, 3.49), Phe 699 (4.72, 5.38), Val 702 (4.33), Ala 719 (4.18), Leu 694 (4.71)	–	Pi interactions (pi Anion, pi–pi stacked, pi alkyl)
	Phe 699 (4.64)	Br	Alkyl
	Lys 721, Gly 772, Arg 817, Asp 776	–	Vander waals
Entry	Amino acid	Ligand involved moiety	Type of interaction
*6h*	Lys 721 (2.96, 1.88), Thr 830 (2.82), Glu 738 (3.66)	Quinazoline & pyrimidine moiety	Hydrogen bond
	Asp 831 (4.0, 3.77), Met 742 (5.96), Phe 699 (3.97, 4.76), Val 702 (4.95, 4.52), Ala719 (5.38), Lys 721 (5.22), Lue 820 (5.94)	Quinazoline & pyrimidine moiety	pi Anion, Pi-Sulfur, Pi–Pi Stacedk, Pi-Alkyl
	Lue 694 (4.33)	Br	Alkyl
	Cys 751, Gly 772, Met 769, Thr 766	–	Vander waals
*6i*	Leu 694 (3.62), Lys 721 (2.96)	Pyrimidine & C = O	Hydrogen bond
	Asp 831 (4.57), leu 694 (3.94), Phe 699 (4.96), Lys 721 (4.08), Leu 694 (3.94), Leu 820 (5.35), phe 699 (4.96), Lys 721 (4.87), Val 702 (4.24, 5.43),Ala 719 (5.11)	–	pi-Anion, , pi-sigma, pi–pi, pi- alkyl
	Lys 721 (4.03), Lue 764 (4.43), Met 742 (5.02)	Br	Alkyl
	Met 769, Thr 766, Gly 695, Gly 772	–	Vander waals
*6j*	Met 769 (3.54), Cys 773 (2.54)	pyrimidine & OCH_3_ moiety	Hydrogen bond
	Lue 820 (3.92), Val 702 (4.97, 5.29), Lys 721 (4.57), Cys 773(4.18), Lue 694 (4.77, 4.09)	Quinazoline, phenyl & pyrimidine moiety	Pi-sigma, Pi-Alkyl
	Met 742 (4.95), Lys 721 (4.26), Lue 764 (3.99)	Br	Alkyl
	Asp 831, Th r830, Glu 738, Gly 772, Lue 768, Pro 770, Ala 719 , Thr 766	–	Vander waals
*6k*	Cys 773 (3.59)	Pyrimidine moiety	Hydrogen bond
	Lys 721 (4.65, 4.33), Leu 820 (5.4, 5.07), Lys 721 (433), Val 702 (4.43), Lue 694 (5.04)	Phenyl rings	pi Cation, Pi-Sigmam, Pi–Sulfur, Pi-Alkyl
	Gly 772, Asp 831, Mrt 742, Thr 830, Thr 766, Glu 738, Ala 719, Lue 764, Glu 738	–	Vander waals
*6l*	Asp 776 (3.61), Asn 818 (3.48)	Pyrimidine moiety	Hydrogen bond
	Asp 831 (3.61), Val 702 (3.85, 4.13), Phe 699 (4.12, 4.30), Lue 694 (5.25), Lue 820 (5.29)	Quinazoline, pyrimidine & pheny moiety	pi Anion, Pi-sigma, Pi–Pi Stacked, Pi-Alkyl
	Met 769 (5.08), Arg 817 (3.76), Lue 820 (4.47), Ala 719 (3.81)	Br & OCH_3_ moiety	Alkyl
	Lys 721, Thr 830, Lue 768, Gly 695	–	Vander waals
*6m*	Ser 888 (2.01), Lys 889 (2.08)	C = O moiety	Hydrogen bond
	Lys 889 (5.35, 4.55)	Quinazoline & Br	Pi-Alkyl and Alkyl
	Pro 912, Pro 913, Arg 779, Asp 892, Lys 889	–	Vander waals
*6n*	Gly 772, (3.46) Lys 721 (2.79)	Quinazoline & phenyl moiety	Hydrogen bond
	Asp 831 (3.89, 3.68), Phe 699 (3.84, 4.58), Ala 719 (5.44), Lys 721 (5.39), Val 702 (4.83, 5.22), lue 820 (5.34)	Quinazoline, pyrimidine & pheny moiety	pi anion, Pi–Pi stacked, Pi-Alkyl
	Lue 694 (3.86)	Cl moiety	Alkyl
	Cys 751, Cys 773, Met 742, Met 769, Thr 830, Thr 766, Gln 767	–	Vander waals
	Gln 738 (3.19)	Pyrimidine moiety	Halogen interaction

**Figure 8:**
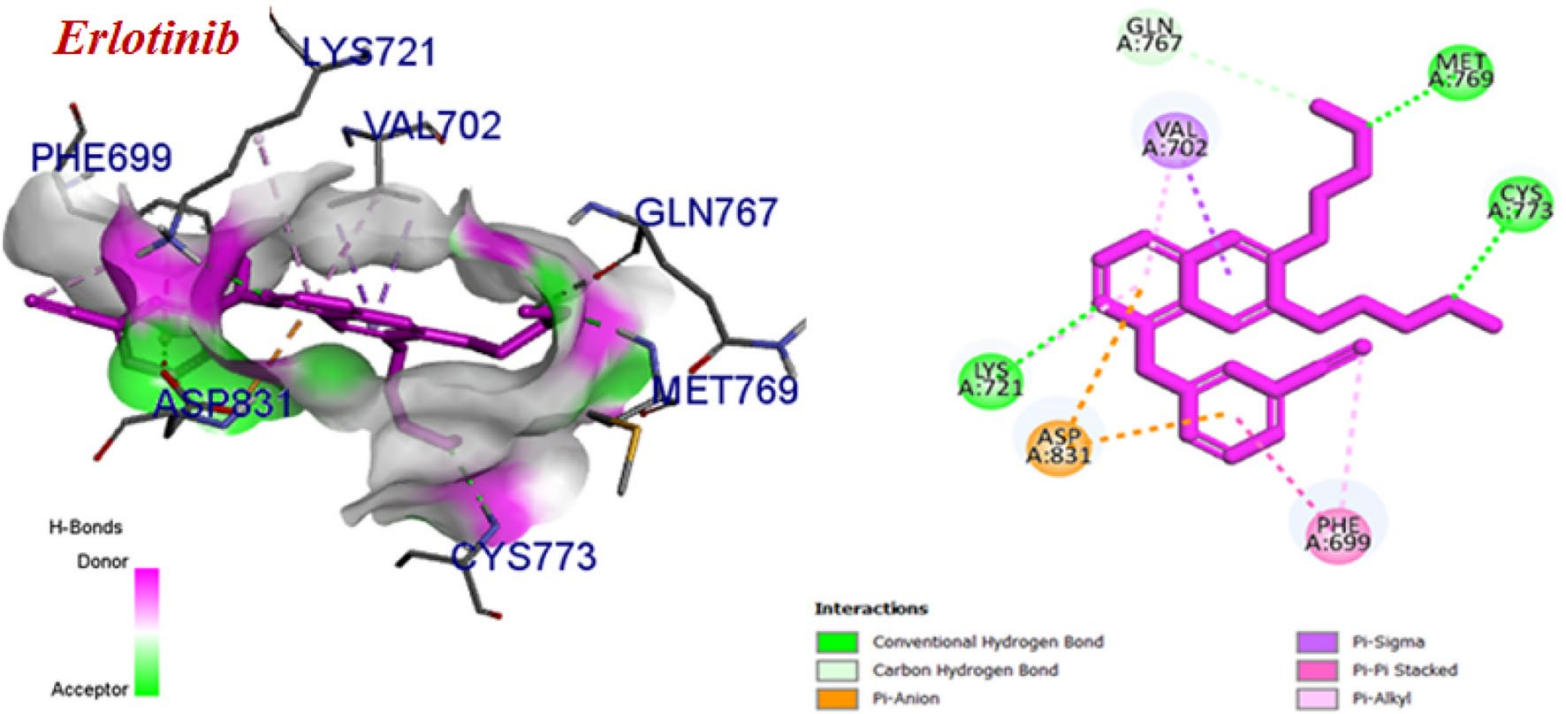
Predicted binding mode of Erlotinib with 1M17.

All of the compounds exhibited better affinity to EGFR protein and also, had higher binding energy compared to Erlotinib. According to the proposed binding mode of Erlotinib (Fig. 8), the planar region of Erlotinib (quinazoline moiety) interacted via hydrogen bonds and pi interactions with Lys 721, Asp 831, and Val 702. Additional hydrogen bond interactions were observed between Cys773, Met769 and Gln 767 and aliphatic chain. Besides, hydrophobic region (phenyl ring) formed pi interactions with Phe 699 and Asp 831.

**Figure 9:**
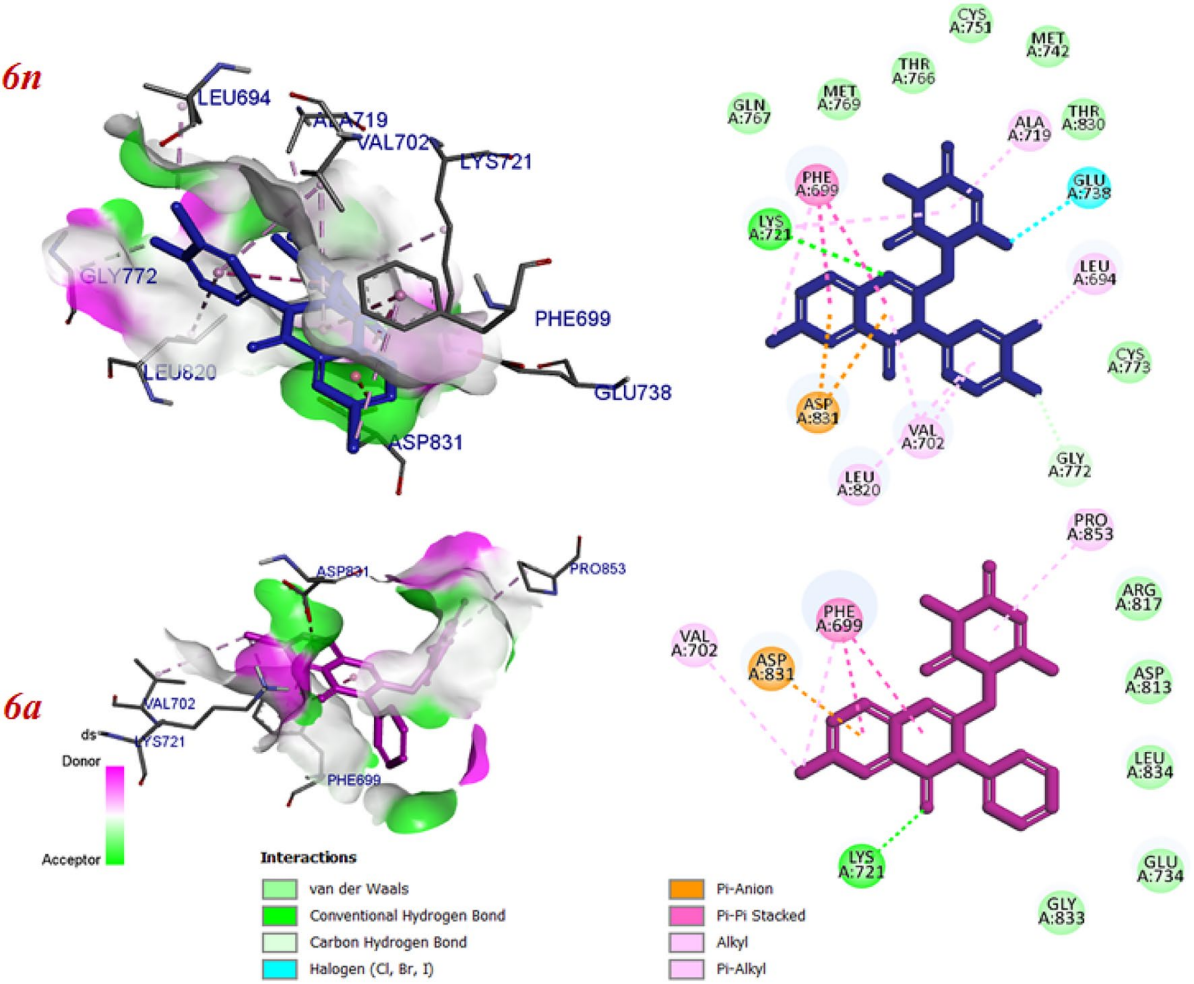
Predicted binding mode of compound 6n and 6a with 1M17.

The binding mode of the most potent compound (6n) and the less active compound (6a) were showed in Fig. 9. In compound 6n, the planar region (quinazoline pharmacophore) interacted via two hydrogen bonds with Lys 721 and Gly 772 and also, formed pi interactions with PHE 699, Asp 831 and Val 702. The pyrimidine moiety involved in pi-alkyl interactions with Lys 721 and ALA 719 and halogen bond with Glu 738. The 3-chloro-4-fluoro phenyl substituents of compound 6n formed two interactions including hydrogen and pi-alkyl with Gly 772, Leu 820, Val 702 and Leu 694. Some hydrophobic interactions with Met 769, Met 742, Gln 767, Thr 766, Thr 830, Cys 751, Cys 773 were also, seen. These additional interactions may explain the reason of the highest cytotoxic activity of compound 6n among the other compounds. The proposed binding mode for compound 6a includes hydrogen bonding and pi interactions with Lys 721 and Asp 831, Phe 699 by the planar quinazoline ring. Additionally, it formed alkyl bond with Val 702 through Br atom. The pyrimidine moiety formed one pi-alkyl interaction with Pro 853 and also, some hydrophobic interactions with Arg 817, Asp 813, Leu 834, Glu 734 and Gly 833 were observed. The results of docking studies showed that most compounds have key amino acids in the active site of EGFR like Erlotinib, such as Cys 773, Lys 721, Val 702, Phe 699.

### Molecular dynamic study

**Figure 10:**
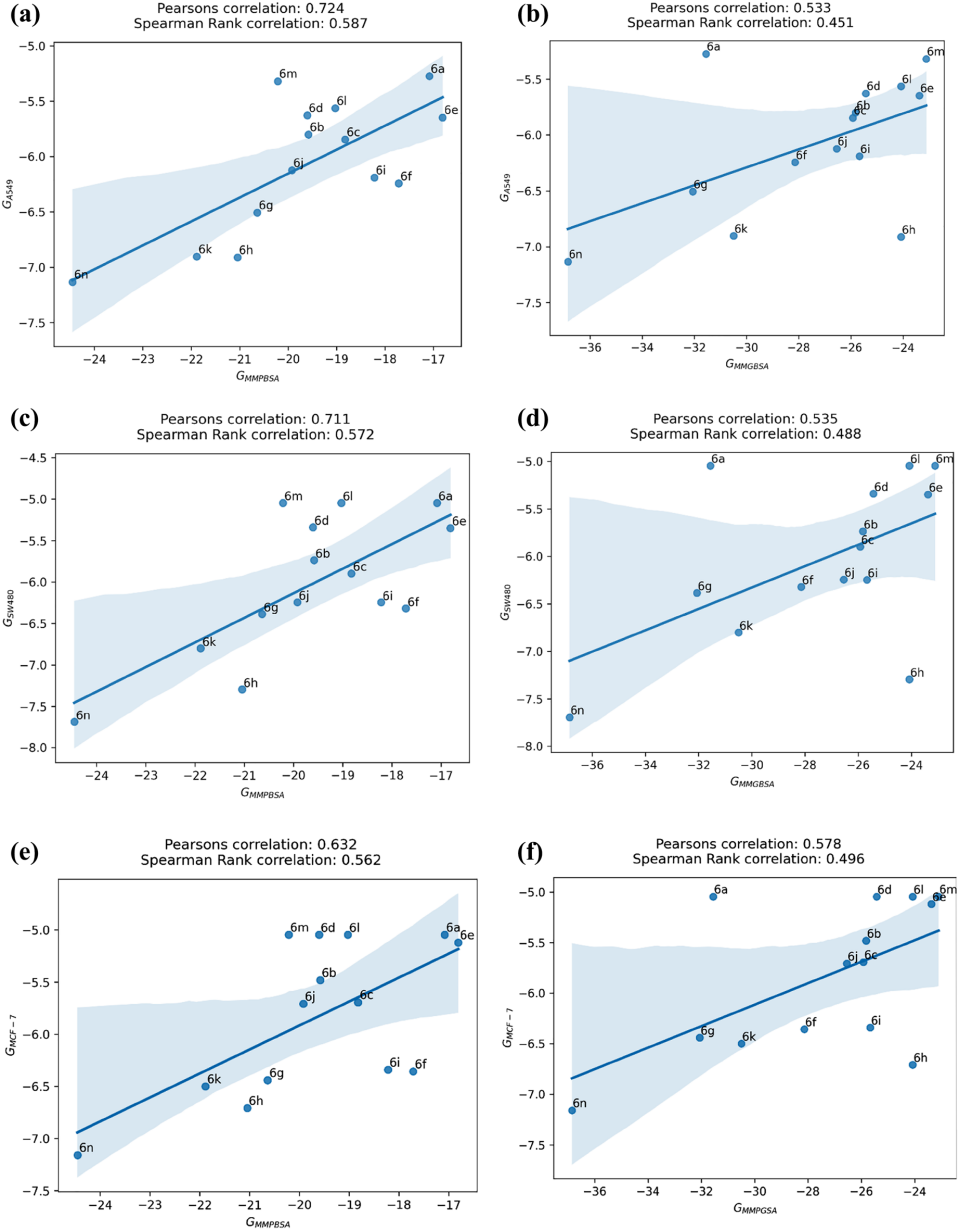
Pearson and Spearman Rank correlation between MMPBSA and experimental binding energy obtained from anti-proliferative activities toward (**a**) A549, (**c**) SW-480, and (**e**) MCF-7, and between MMGBSA and experimental binding energy obtained from anti-proliferative activities toward (**b**) A549, (**d**) SW-480 and (**f**) MCF-7.

The MMPB/GBSA binding energies of all ligands (6a-6n) and the corresponding experimental binding energies obtained from the cytotoxicity data related to A549, SW-480 and MCF-7 cell lines are shown in Table 4. All binding free energy values are ordered based on experimental values of A549 cell line. Compound 6n has the maximum binding energy for both MMPBSA and MMGBSA and this is in line with cytotoxicity (IC_50_) values after converting them to binding energies using ΔG =  − RT ln (IC_50_) equation. Pearson and Spearman's correlation coefficients were used to measure the correlation between values of MMPB/GBSA and experiment data (Fig. 10). The Pearson correlation coefficient (r) is known as the most usable correlation coefficient that summarizes the characteristics of a dataset. Also, it describes the strength and linear relationship direction between two quantitative variables. Spearman's rank correlation is a nonparametric statistical dependence coefficient between the rankings of two variables. Pearson coefficient works with a linear relationship between the two variables whereas the Spearman coefficient works with monotonic relationships as well. On the other hand, Pearson works with raw data values of the variables whereas Spearman works with rank-ordered variables. Conferring to Fig. 10, MMPBSA results correlate well with experimental binding energies according to its Pearson (r_P_ = 0.724, 0.711 and 0.632 for A549, SW-480 and MCF-7, respectively) and Spearman correlation coefficient (r_s_ = 0.533, 0.535 and 0.578 for A549, SW-480 and MCF-7, respectively). In addition, the correlation is stronger when we rank MMPBSA binding energy values with binding energies obtained from the cytotoxicity of A549 cell lines (Fig. 10a). Pearson correlation predicts the binding energy ranking of ligands 6n, 6 h, 6 k, 6 g, 6f. and 6a in the correct order. These ligands are chosen for further analysis.

**Table 4:** MMPB/GBSA binding energy and experimantal bining energy obtained from anti-proliferative activities toward (a) A549, (b) SW-480, and (c) MCF-7 for selected ligands (in kcal/mol). *Experimental ∆G was converted with the relation ∆*G* =  − *RT* ln (IC_50_). Data in kcal/mol.

Compound	ΔG_MMPBSA_	ΔG_MMGBSA_	ΔG* _exp, A549_	ΔG* _exp, SW480_	ΔG* _exp, MCF−7_
6n	− 24.45	− 36.85	− 7.133	− 7.691	− 7.159
6 h	− 21.04	− 24.07	− 6.911	− 7.294	− 6.708
6 k	− 21.88	− 30.50	− 6.903	− 6.800	− 6.499
6 g	− 20.63	− 32.06	− 6.506	− 6.384	− 6.440
6f.	− 17.71	− 28.13	− 6.243	− 6.320	− 6.356
6i	− 18.21	− 25.66	− 6.190	− 6.246	− 6.340
6j	− 19.92	− 26.54	− 6.122	− 6.243	− 5.708
6c	− 18.82	− 25.92	− 5.846	− 5.898	− 5.694
6b	− 19.58	− 25.82	− 5.800	− 5.735	− 5.481
6e	− 16.81	− 23.37	− 5.647	− 5.348	− 5.120
6d	− 19.60	− 25.42	− 5.627	− 5.339	− 5.046
6 l	− 19.02	− 24.07	− 5.563	− 5.046	− 5.046
6 m	− 20.21	− 23.10	− 5.320	− 5.046	− 5.046
6a	− 17.08	− 31.55	− 5.274	− 5.046	− 5.046

#### Binding free energy, energy decomposition analysis, hydrogen bond and clustering analysis

**Figure 11:**
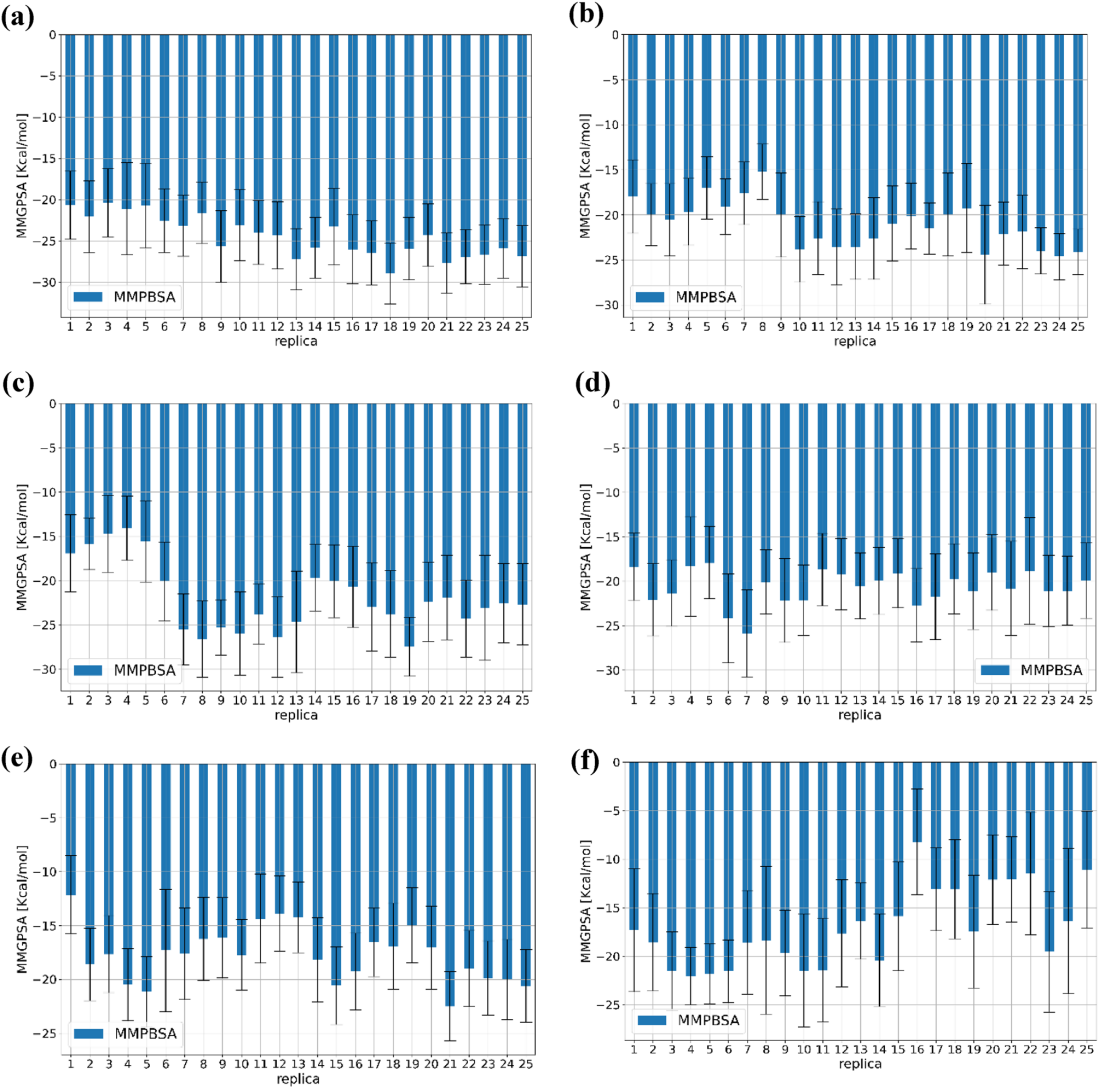
Binding free energy of (**a**) 6n (**b**) 6h (**c**) 6k (**d**) 6g (**e**) 6f. and (**f**) 6a using ensemble based free energy calculation.

To show the factors responsible for the inhibitor activities of **6n**, **6 h**, **6 k**, **6 g**, **6f.** and **6a** ligands, the MM/PBSA method was run. Figure 11 illustrated the changing amount of binding free energy for each replica in the ensemble as well as the associated uncertainties. The final binding free energy can be estimated by the average binding energy of each replica. The total binding free energies are found in a range of − 24.45 and − 17.08 kcal·mol^−1^ (Table 5). Table 4 shows the contribution terms in the binding free energies of these inhibitors against EGFR target. The more contributed of the net binding free energy of 6n is belong to VDW energy term with − 53.56 kcal·mol^−1^ energy. Another contribution is G_Non-Polar_ term which is essentially always small and similar for all selected ligands (ranging from − 4.71 to − 3.90), the intermolecular electrostatic interactions is normally calculated using Coulomb’s law with atomic charges taken from the MM force field. Consequently, the results depend on the charges used for the receptor and the ligand. This energy term has a more positive effect on binding energy of 6n and 6 k (− 22.42 and − 19.51 kcal·mol^−1^).

**Table 5:** Binding free energy and its components obtained by MMPBSA calculation for all ligands.

	6n	6 h	6 k	6 g	6f	6a
MMPBSA						
E_VDW_	− 53.56	− 37.59	− 47.31	− 48.45	− 45.34	− 48.41
E_elec_	− 22.42	− 14.26	− 19.51	− 16.26	− 7.51	− 10.19
E _PB_	56.24	34.72	49.59	48.75	39.48	46.11
G_Non−Polar_	− 4.71	− 3.90	− 4.65	− 4.66	− 4.34	− 4.59
ΔG_gas_	− 75.99	− 51.86	− 66.82	− 64.72	− 52.85	− 58.60
ΔG_solv_	51.53	30.81	44.94	44.08	35.14	41.52
ΔG_Bind_	− 24.45 ± 2.7	− 21.04 ± 2.7	− 21.88 ± 2.7	− 20.63 ± 2.7	− 17.71 ± 2.7	− 17.08 ± 2.7

**Figure 12:**
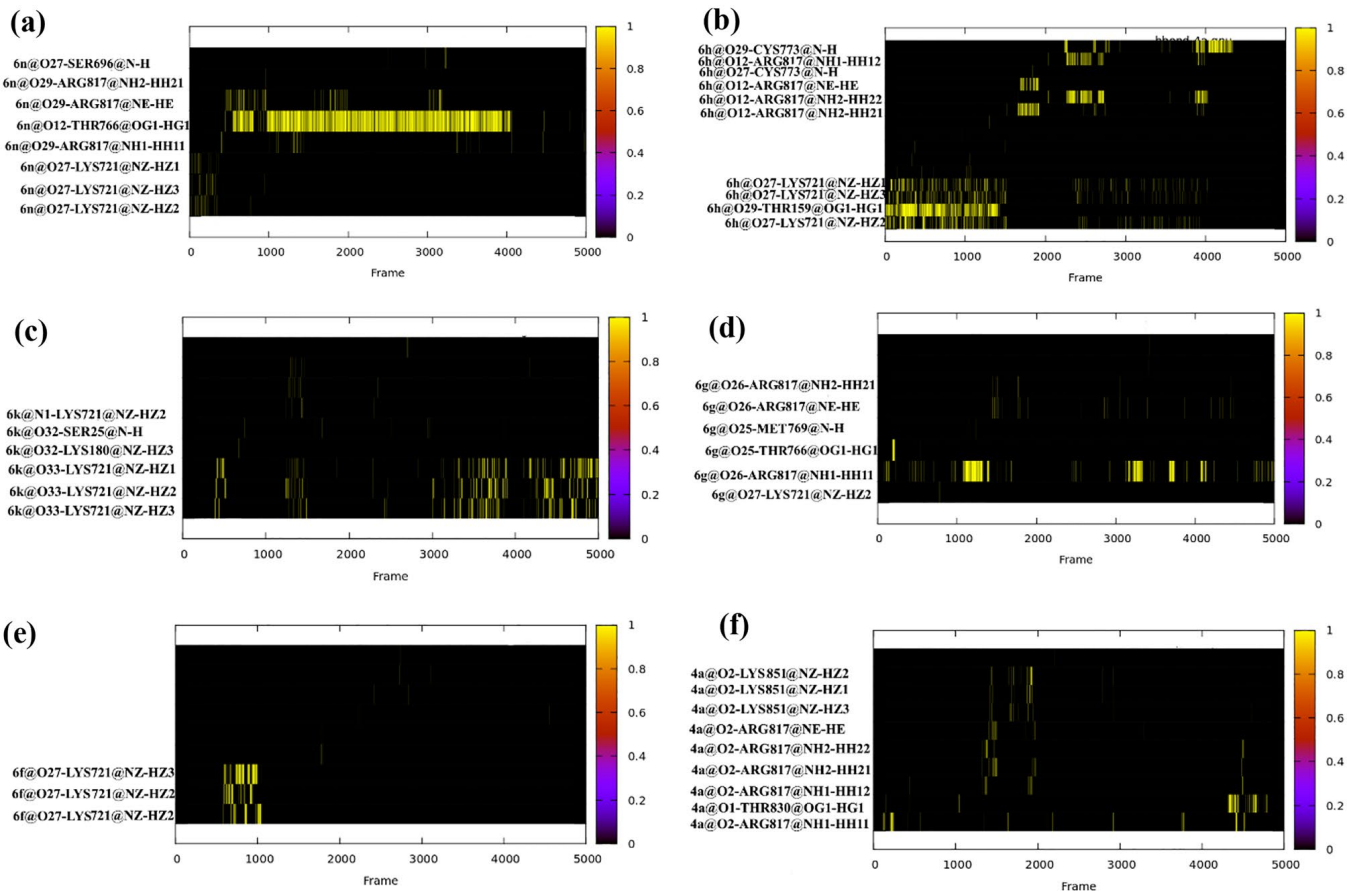
Hydrogen bonding occupancy plots versus time for (**a**) *6n* (**b**) *6 h* (**c**) *6 k* (**d**) *6 g* (**e**) *6f.* and (**f**) *6a.*

The strength of intermolecular electrostatic interactions is depending on the number of hydrogen bonds, water bridges and other polar interactions between the protein and the ligand. To evaluate the type and strength of hydrogen bonds between selected ligands and the protein, MD simulation of ligand-enzyme was accomplished during the whole ensemble-based trajectories. We used the H-bond module of AmberTools 22 to check the hydrogen-bonding profiles between the chosen ligands and the enzymes. As default values, the angle and H-bonding distance threshold were set to 135’ and 3.0 Å, respectively. Figure 12 demonstrates the hydrogen bonding occupancy plots versus time for all aforementioned ligands. The number of hydrogen bonds for all compounds were varied between 0 and 1. Compounds 6n, 6 h and 6 k with the highest number of hydrogen bonds and the highest occupancies are expected to have stronger interactions. By exact inspection of hydrogen bonds from all selected ligands, we found Arg 817 and Lys 721 had strong hydrogen bonds with oxygen atom of quinazolinone motif which has pivotal role in binding of these ligands. It is supposed that the close contacts between oxygen in the pyrimidine ring (O12) of 6n with –OG atom of Thr 766 has the highest occupancies during the MD simulation (Fig. 12a). Likewise, the same contact between oxygen in pyrimidine ring (O12) of 6 h with –NH group of Arg 817 with moderate occupancies acknowledged its high potency toward EGFR (Fig. 12b). It is believed that another interaction which has a great contribution to the inhibition activities of these ligands is forming hydrogen bond between –NH_2_ group of Lys 721 with the oxygen atom of the quinazolinone group of these ligands. Similarly, compound 6 g forms the close contact between oxygen atom of its quinazolinone group and –NH_2_ group in Arg 721.

**Figure 13:**
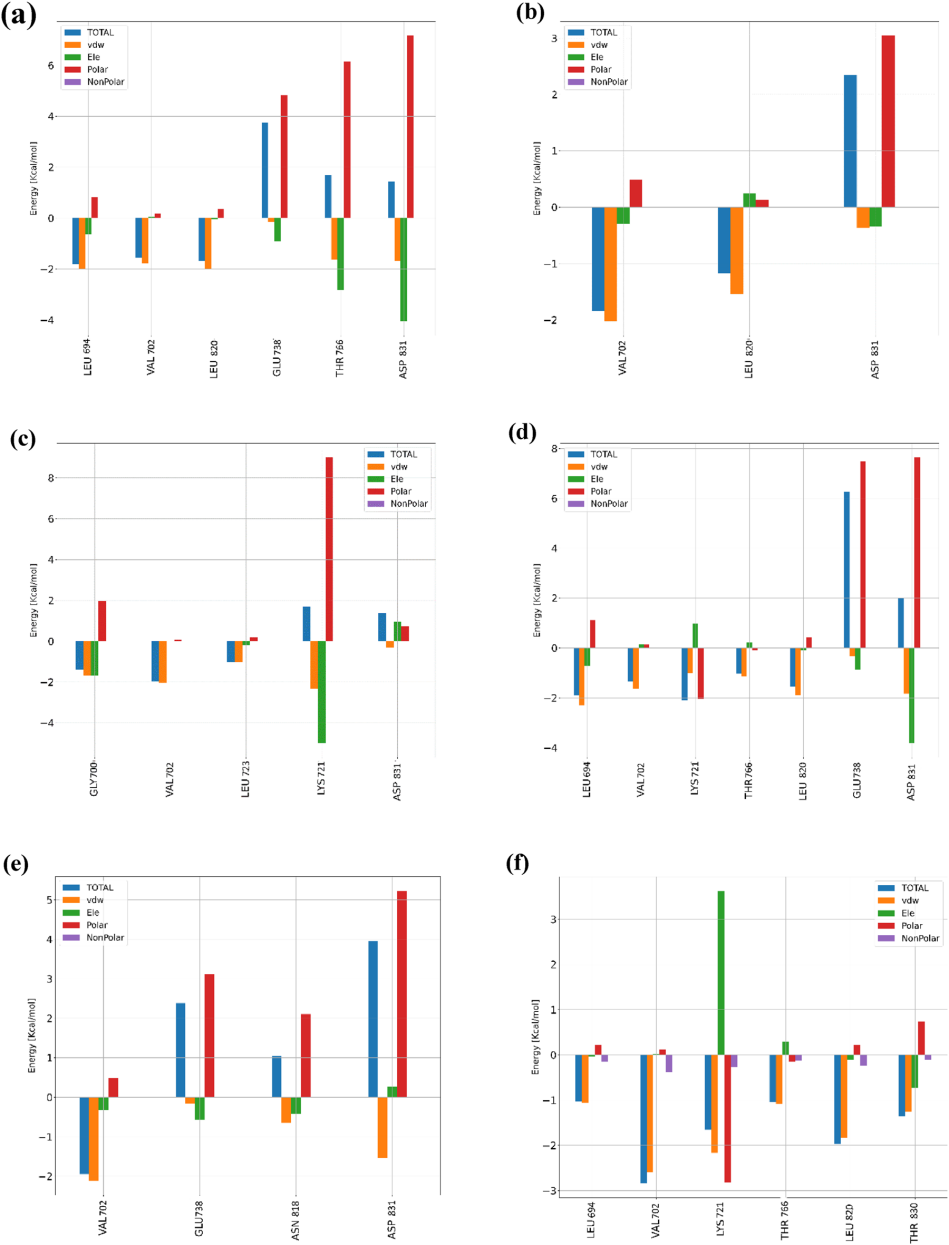
Decomposition of binding free energies for the EFGR and (**a**) 6n (**b**) 6 h (**c**) 6 k (**d**) 6 g (**e**) 6f. and (**f**) 6a complexes into contributions from individual residues.

The MMGBSA formulation can provide supplementary information to evaluate the comparative contribution of every residue toward the binding free energy of the enzyme/ligand complex. Val 702 and Asp 831 are a common contribution in the binding energy of 6n, 6 h and 6 k in the active site of EFGR target. Among these residues, Val 702 plays a key role in the stablishing of these ligands through VDW interactions. However, Asp 831 disfavours the binding of these ligands through their high polar solvation energy term (Fig. 13).

**Figure 14:**
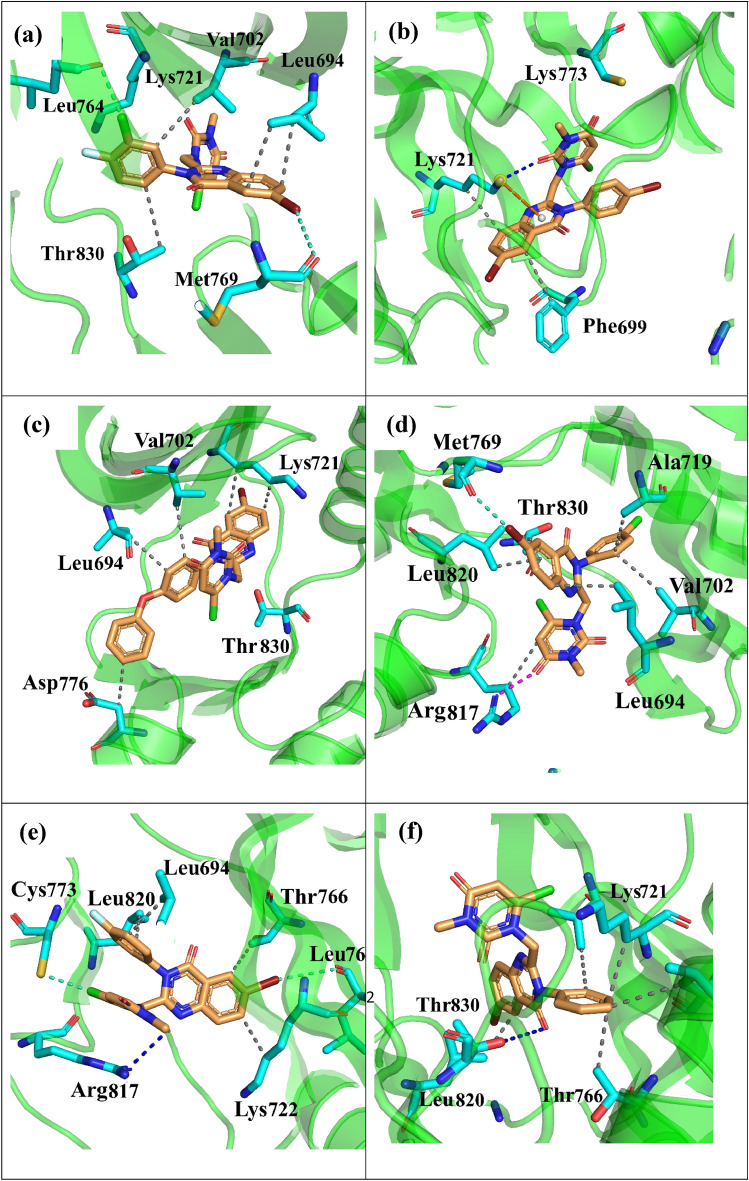
Representative binding modes obtained from clustring analysis for (**a**) *6n* (**b**) *6 h* (**c**) *6 k* (**d**) *6 g* (**e**) *6f.* and (**f**) *6a*.

Conformational clustering was conducted on the molecular dynamic trajectories of the previously described ligand–protein complexes to select intellectual representative conformations for advanced evaluation^32–34^. Clustering was carried out using k-means algorithms and the RMSD of residues with a sieving frame of 10. The representative structures of the more populated cluster were choosing as a model to illustrate the much often interactions between aforementioned ligands and the active site of EGFR. The results depicted in Fig. 14a,b,c,d,e,f, nevertheless, these models do not necessarily reflect all main interactions and can be refer to the other analysis such as H-bond analysis for more accurate data. According to Fig. 14, visual analysis of the protein active site discloses 2 primary interactions that influence the accommodation of aforementioned ligands in the active site: hydrophobic "clamp" that provides affinity; and hydrogen-bonding network that determines binding mode. Figure 14a shows the halogen bond manifestation between bromine atom of 6n and Met 769. According to Fig. 14a, residues such as Leu 694, Val 702, Lys 721, Leu 764, Thr 830 stabilize the 6n in the active site of enzyme by forming several alkyl-alkyl staking interaction and this is in line with what we observed in decomposition analysis of this ligand. Figure 14b shows the main electrostatic interactions of 6 h including H-bond between oxygen atom in quinazolinone moiety with Lys 721 (Fig. 14b). In addition, the π-π and alkyl-alkyl stacking interactions between pyrimidine moiety and -NH_2_ group of Lys 721 and Phe 699 is participated in ligand stabilization. Although hydrogen bond analysis proved forming of H-bond between oxygen atom in quinazolinone moiety of the 6 k with Lys 721, same as the 6 h, this is not appearing in its interaction map of representative configuration. Figure 14c illustrates the possible hydrophobic cavities involving Leu 694, Val 702, Lys 721, Asp 776 and Thr 830 with 6 k in the active site of the protein. Figure 14d and 12e show the representative configuration of 6 g and 6f., respectively. 6 g and 6f. had the same configuration in the active site of the protein. These two structures formed H-bond between pyrimidine oxygen atoms with Arg 817. Despite the fact that the main interaction, is forming H-bond between quinazoline oxygen atom with Lys 721, this interaction has not been considered as a stable one for 6f. justifying the underestimation of MMPBSA free energy for this ligand in our calculation (Fig. 14d,e).

#### In silico Physicochemical parameter (ADME) prediction

In this part of the study, all the synthesized compounds (6a-6n) and Erlotinib were conducted to determine their physicochemical properties using http://www.swissadme.ch/ online server (Table 6). According to the Lipinski’s rule, compound's absorption will be better if they perform at least three of these following rules: all of the compounds fulfilled the Lipinski rule except molecular weight (MW) of some compounds.

**Table 6:** Physicochemical properties of Erlotinib and synthesized compounds.

Compound	MW^a^	LogP^b^	HBD^c^	HBA^d^	TPSA (A^2^) ^e^	*n*-RB^f^	Lipinski violation
6a	473.71	3.40	0	4	78.89	3	0
6b	49.72	2.76	0	5	102.68	3	0
6c	491.70	3.78	0	5	78.89	3	0
6d	508.15	3.88	0	4	78.89	3	1
6e	552.60	3.99	0	4	78.89	3	1
6f.	491.70	3.78	0	5	78.89	3	0
6g	508.15	3.88	0	4	78.89	3	1
6h	552.60	3.99	0	4	78.89	3	1
6i	487.73	3.62	0	4	78.89	3	0
6j	503.73	2.83	0	5	88.12	4	1
6k	565.8	4.18	0	5	88.12	5	1
6l	533.76	2.53	0	5	98.35	5	1
6m	501.76	3.82	0	4	78.89	4	0
6n	526.14	4.26	0	5	78.89	5	1
Erlotinib	393.44	1.89	1	6	74.73	10	0
*Rule of Lipinski*	≤ 500	≤ 5	≤ 5	≤ 10	≤ 140	≤ 10	≤ 1

In silico study of all compounds and Erlotinib were evaluated for ADME properties by using preADMET online server (www.preadmet.bmdrc.org). According to ADME profiles and the obtained results (Table 7), we can suggest that the synthesized compounds have better intestinal absorption in humans (97.54–98.24) than Erlotinib (96.28). Preferred properties of these compounds enable them to facilitate the movement across various biological membranes (30). The Caco-2 permeability parameters indicate that all compounds have moderate ability to penetrate biological membranes. Also, the designed compounds demonstrate low to moderate crossing of the blood–brain barrier (0.58–1.92). All compounds similar to Erlotinib have relatively high binding to plasma protein. Furthermore, compounds 6k and 6l can inhibit cytochrome P3A4, which is the main enzyme involved in the metabolism of drugs, while Erlotinib doesn’t have this ability.

**Table 7:** ADME profile of Erlotinib and the synthesized compounds.

Compound	Absorption			Distribution		Metabolism
	% HIA^a^	In vitro Caco-2 cell permeability (nm s^−1^)	In vitro Skin permeability ((log Kp, cm h^−1^)	% In vitro plasma protein bonding	%BBB^b^	CYP2C19 inhibitor, CYP2C9 inhibitor, CYP2D6 inhibitor, CYP3A4 inhibitor
6a	97.62	27.51	− 3.62	98.15	0.58	No, No, No, No
6b	98.24	21.87	− 3.44	100	0.78	No, No, No, No
6c	97.54	27.91	− 3.83	100	0.74	No, No, No, No
6d	97.73	29.61	− 3.45	99.23	0.80	No, No, No, No
6e	97.90	30.93	− 3.29	97.47	0.80	No, No, No, No
6f.	97.54	27.91	− 3.83	98.57	1.80	No, No, No, No
6g	97.73	29.72	− 3.45	99.41	1.92	No, No, No, No
6h	97.90	31.25	− 3.29	96.77	1.90	No, No, No, No
6i	97.57	28.72	− 3.49	98.74	0.81	No, No, No, No
6j	97.61	58.69	− 3.70	97.40	1.33	No, No, No, No
6k	97.85	33.44	− 2.73	98.43	0.75	No, Yes, No, Yes
6l	97.84	29.87	− 3.85	95.10	1.09	No, No, No, No
6m	97.60	29.95	− 3.45	96.85	0.75	No, Yes, No, Yes
6n	97.74	30.10	− 3.75	97.29	1.63	No, No, No, No
Cisplatin	96.28	54.87	− 2.86	93.15	0.04	No, No, No, No

## Conclusion

In summary, we designed and synthesized some new quinazoline-pyrimidine hybrid derivatives incorporating different phenyl moieties at position 3 of the quinazoline ring (*N-*3). All the synthesized compounds (6a-6n) were successfully characterized using IR, NMR (^1^H & ^13^C) and Mass spectroscopic techniques. The antiproliferative activities were examined against a panel of three human cancer cell lines (A549, SW-480, and MCF-7) using MTT assay. Among the tested compounds, 6n showed the highest antiproliferative activities against the tested cell lines. This compound could also induce apoptosis in A549 cell line in a dose dependent manner, and also could arrest the cells in the S phase of cell cycle. Molecular docking study and predicting the possible interactions between target compounds and EGFR showed that the binding site of the proposed compounds with EGFR active site and docking simulation were in good agreement with the results of biological screening. The ensemble-based MMPB/GB calculations was used to inhibitory ranking of all synthesized ligands against different cell lines. The MMPBSA calculation is well correlated with the cytotoxic activity in A549 cell line. We used the ensemble trajectories for H-bond, clustering and energy decomposition analysis to investigate the vital residues associated with the binding of ligand in the active site of the protein. Accordingly, we found the crucial role of Arg 817 and Lys 721 in binding affinities of potent ligands (6n and 6h). Furthermore, the ADME study was calculated and the designed compounds were shown to be compatible with the Lipinski's rule.

## Experimental

### Chemistry

All solvents and reagents were obtained from the Sigma, Aldrich and Merck. Reaction progress was monitored using thin-layer chromatography (TLC) on silica gel plates. Melting points were measured by Electrothermal 9200 apparatus (Electrothermal, UK). Infrared spectra (KBr) were recorded on a VERTEX70 spectrometer (Bruker, Germany). ^1^HNMR and ^13^CNMR spectra were obtained using VARIAN-INOVA 500 MHz Bruker in CDCl_3_ or DMSO‑d6 solution. Mass spectra were determined with Agilent Technologies (HP).

### General procedure for the synthesis of 2-amino-5-bromobenzoic acid (2)

Anthranilic acid (1) (1 mmol) was treated with *N*-bromo succinimide (1.2 mmol) in acetonitrile at room temperature for 2 h, then the remained solvent was removed by vacuum evaporation and the reaction mixture washed with acetonitrile to give the pure white solid.

### General procedure for the synthesis of 6-bromo-2-(chloromethyl)-4H-benzo[d][1,3]oxazin-4-one (3)

2-Amino-5-bromobenzoic acid (2) (1 mmol), diisopropylethylamine (DIPEA) (1.5 mmol) and chloroacetyl chloride (1.2 mmol) were stirred in dichloromethane (10 mL) at room temperature for 2 h. After completion the reaction, 20 mL H_2_O was added to the reaction mixture and extracted with ethyl acetate (3 × 20 mL). The organic layers were dried by sodium sulfate and concentrated under vacuum to give the white solid compound.

### General procedure for the synthesis of 6-bromo-2-(chloromethyl)-3-substituted quinazoline-4(3H)-one (5a-5n)

Intermediate 3 was reacted with various substituted anilines (4a-4n) (1 mmol) under acidic condition in the presence of 1.5 mmol PCl_3_ through refluxing in acetonitrile (CH_3_CN) at 80 °C for 24 h. Then the reaction was poured into a saturated NaHCO_3_ solution and extracted with ethyl acetate (3 × 20 mL). The organic layers were dried by sodium sulfate and concentrated under vacuum to give the white solid compounds.

### General procedure for the synthesis of 1-((6-bromo-4-oxo-3- substituted -3,4-dihydroquinazolin-2-yl) methyl)-6-chloro-3-methylpyrimidine-2,4(1H,3H)-dione (6a–6n)

**Figure 15:**
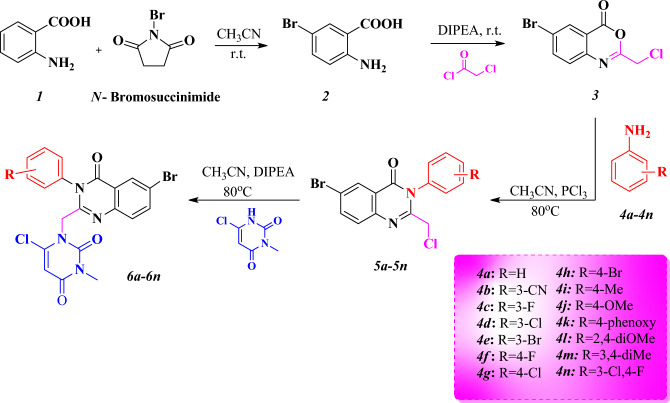
Synthesis of 1-((6-bromo-4-oxo-3- substituted -3,4-dihydroquinazolin-2-yl) methyl)-6-chloro-3-methylpyrimidine-2,4(1H,3H)-dione (6a–6n).

In the final step, intermediates 5a–5n (2 mmol) and 6-chloro-3-methyl uracil (2 mmol) were refluxed in acetonitrile in the presence of 1.5 mmol DIPEA at 80 °C for 24 h. After completion the reaction, the crude products extracted with ethyl acetate (3 × 20 mL). The organic layers were dried by sodium sulfate and concentrated under vacuum to give the final target compounds (Fig. 15)***.***

*1-((6-Bromo-4-Oxo-3-Phenyl-3,4-Dihydroquinazolin-2-Yl)Methyl)-6-Chloro-3-Methylpyrimidine-2,4(1H,3H)-Dione (6a)*.IR (KBr, cm^−1^): 2961 (CH), 2928 (CH), 1715 (C = O), 1684 (C = O), 1670 (C = O), 1611 (C = N), 1519 (C = C), 1476 (C–N), 1441 (C–O), 847 (C–Br). ^1^H NMR (500 MHz, CDCl_3_) *δ*_H_ (ppm): 8.35 (s, 1H, H-5-quinazoline), 7.81 (d, 1H, *J* = 10 Hz, H-7-quinazoline), 7.467 (d, 1H, *J* = 10 Hz, H-8-quinazoline), 7.36–7.39 (m, 3H, phenyl), 7.31 (t, 2H, *J* = 10 Hz, phenyl, 6.01 (s, 1H, uracil), 4.81 (s, 2H, quinazoline-CH_2_-uracil), 3.33 (s, 3H, CH_3_). ^13^C NMR (125 MHz, CDCl_3_) *δ*_C_ (ppm): 164.31, 162.30, 160.71, 151.12, 149.87, 145.62, 145.43, 137.95, 131.05, 130.01, 129.94, 129.65, 129.53, 122.33, 121.12, 117.84, 117.66, 102.38, 47.68, 28.47.

*3-(6-Bromo-2-((6-Chloro-3-Methyl-2,4-Dioxo-3,4-Dihydropyrimidin-1(2H)-Yl)Methyl)-4-Oxoquinazolin-3(4H)-Yl) Benzonitrile (6b).* IR (KBr, cm^−1^): 3065 (CH), 2957 (CH), 2239 (C≡N), 1719 (C = O), 1674 (C = O), 1666 (C = O), 1611 (C = N), 1580 (C = C), 1464 (C–N), 1437 (C–O), 847 (C–Br). ^1^H NMR (500 MHz, CDCl_3_) *δ*_H_ (ppm): 8.33 (s, 1H, H-5-quinazoline), 7.89 (d, 1H, *J* = 10 Hz, H-7-quinazoline), 7.83(d, 1H, *J* = 10 Hz, H-8-quinazoline), 7.76–7.79 (m, 1H, phenyl), 7.72 (s, 1H, phenyl), 7.67 (d, 1H, *J* = 5 Hz, phenyl), 7.49 (d, 1H, *J* = 5 Hz, phenyl), 6.02 (s, 1H, uracil), 4.72–4.81 (m, 2H, quinazoline-CH_2_-uracil), 3.32 (s, 3H, CH_3_). ^13^C NMR (125 MHz, CDCl_3_) *δ*_C_ (ppm): 160.60, 160.40, 151.04, 149.03, 145.45, 145.26, 138.27, 136.25, 133.90, 132.94, 131.88, 131.60, 129.73, 129.51, 122.08, 121.47, 117.02, 114.99, 102.46, 47.60, 28.47. MS (*m/z*, %): 499.0 (M + 1, 9.89)^+^, 464.0 (100), 406.0 (30.44), 377.0 (11.16), 339.0 (62.34), 259.1 (39.07), 221.0 (20.85), 170.0 (15.87).

*1-((6-Bromo-3-(3-Fluorophenyl)-4-Oxo-3,4-Dihydroquinazolin-2-Yl)Methyl)-6-Chloro-3-Methylpyrimidine-2,4(1H,3H)-Dione (6c).* ^1^H NMR (500 MHz, CDCl_3_) *δ*_H_ (ppm): 8.36 (s, 1H, H-5-quinazoline), 7.82 (d, 1H, *J* = 5 Hz, H-7-quinazoline), 7.63–7.59 (m, 1H, phenyl) , 7.48 (d, 1H, *J* = 5 Hz , H-8-quinazoline), 7.31 (t, 1H, *J* = 10 Hz, phenyl), 7.20 (d, 1H, *J* = 10 Hz, phenyl), 7.14 (d, 1H, phenyl), 6.01 (s, 1H, uracil), 4.79–4.88 (m, 2H, quinazoline-CH_2-_uracil), 3.33 (s, 3H, CH_3_). ^13^C NMR (125 MHz, CDCl_3_) *δ*_C_ (ppm): 164.41, 162.40, 160.69, 160.42, 151.10, 149.50, 145.57, 138.01, 136.49, 131.92, 129.66, 123.96, 122.30, 121.19, 117.76, 116.02, 115.84, 102.38, 47.57, 28.46. MS (*m/z*, %): 492.1 (M + 1, 13.60)^+^, 457.1 (100), 399.1 (33.63), 332.0 (31.72), 252.1 (28.50), 223.0 (26.51), 170.0 (2.53), 75.1 (9.23).

*1-((6-Bromo-3-(3-Chlorophenyl)-4-Oxo-3,4-Dihydroquinazolin-2-Yl)Methyl)-6-Chloro-3-Methylpyrimidine-2,4(1H,3H)-Dione (6d).* IR (KBr, cm^−1^): 2961 (CH), 2928 (CH), 1709 (C = O), 1680 (C = O), 1665 (C = O), 1609 (C = N), 1587 (C = C), 1468 (C–N), 1437 (C–O), 837 (C–Br). ^1^H NMR (500 MHz, CDCl_3_) *δ*_H_ (ppm): 8.36 (s, 1H, H-5-quinazoline), 7.82 (d, 1H, *J* = 10 Hz, H-7-quinazoline), 7.57 (s, 2H, phenyl), 7.48 (d, 1H, *J* = 10 Hz, H-8-quinazoline), 7.41 (s, 1H, phenyl), 7.30 (s, 1H, phenyl), 6.01 (s, 1H, uracil), 4.83 (s, 2H, quinazoline-CH_2_-uracil), 3.33 (s, 3H, CH_3_). ^13^C NMR (125 MHz, CDCl_3_) *δ*_C_ (ppm): 160.70, 160.44, 151.10, 149.47, 145.57, 145.41, 138.04, 136.27, 136.26, 131.54, 130.71, 129.67, 129.53, 128.44, 126.37, 122.28, 121.22, 102.39, 47.66, 28.48. MS (*m/z*, %): 508.1 (M^+^, 0.85), 473.1(100), 416.1 (24.38), 348 (50.29), 233.1 (40.02), 111.1 (23.59).

*1-((6-Bromo-3-(3-Bromophenyl)-4-Oxo-3,4-Dihydroquinazolin-2-Yl)Methyl)-6-Chloro-3-Methylpyrimidine-2,4(1H,3H)-Dione (6e).* IR (KBr, cm^−1^): 2965 (CH), 2922(CH), 1709 (C = O), 1680 (C = O), 1661 (C = O), 1605 (C = N), 1574 (C = C), 1472(C–N), 1431 (C–O), 835 (C–Br)*.* ^1^H NMR (500 MHz, CDCl_3_) *δ*_H_ (ppm): 8.36 (s, 1H, H-5-quinazoline), 7.83 (d, 1H, *J* = 10 Hz, H-7-quinazoline), 7.73 (d, 1H, *J* = 10 Hz, H-8-quinazoline), 7.56 (s, 1H, phenyl), 7.48–7.52 (m, 2H, phenyl), 7.34 (d, 1H, *J* = 5, phenyl), 6.02 (s, 1H, uracil), 4.83 (s, 2H, quinazoline-CH_2_-uracil), 3.34 (s, 3H, CH_3_). ^13^C NMR (125 MHz, CDCl_3_) *δ*_C_ (ppm): 160.71, 160.44, 151.09, 149.45, 145.55, 145.40, 138.04, 136.37, 133.61, 131.75, 131.22, 129.67, 129.54, 126.83, 123.94, 122.27, 121.23, 102.39, 47.67, 28.48. MS (*m/z*, %): 551.9 (M^+^, 10.18), 516.9 (100), 459.9 (24.65), 391.9 (56.95), 312.0 (23.77), 233.1 (30.9), 157.0 (14.68).

*1-((6-Bromo-3-(4-Fluorophenyl)-4-Oxo-3,4-Dihydroquinazolin-2-Yl)Methyl)-6-Chloro-3-Methylpyrimidine-2,4(1H,3H)-Dione (6f.).* IR (KBr, cm^−1^): 2964 (CH), 2922(CH), 1715(C = O), 1686 (C = O), 1670 (C = O), 1607 (C = N), 1510 (C = C), 1473 (C–N), 1441 (C–O), 847(C–Br). ^1^H NMR (500 MHz, CDCl_3_) *δ*_H_ (ppm): 8.35 (s, 1H, H-5-quinazoline), 7.79 (d, 1H, *J* = 10 Hz, quinazoline), 7.44 (d, 2H, *J* = 15 Hz, phenyl), 7.24 (s, 2H, quinazoline), 7.08 (d, 2H, *J* = 10 Hz, phenyl), 5.98 (s, 1H, uracil), 4.81 (s, 2H, quinazoline-CH_2_-uracil), 3.86 (s, 3H, CH_3_). ^13^C NMR (126 MHz, DMSO-*d*_6_) *δ*_C_ (ppm): 161.05, 160.92, 160.06, 155.15, 155.11, 151.05, 145.58, 137.83, 131.60, 130.96, 130.87, 129.83, 128.49, 122.68, 120.16, 116.56, 116.33, 105.36, 66.88, 27.91; MS (*m/z*, %): 490.1 (M^+^, 10.72), 457.2 (100), 399.1 (81.43), 331.1 (29.89), 223.1 (31.89), 170.0 (16.87).

*1-((6-Bromo-3-(4-Chlorophenyl)-4-Oxo-3,4-Dihydroquinazolin-2-Yl)Methyl)-6-Chloro-3-Methylpyrimidine-2,4(1H,3H)-Dione (6g).* IR (KBr, cm^−1^) : 2959 (CH), 2922 (CH), 1713 (C = O), 1682 (C = O), 1674 (C = O), 1609 (C = N), 1493 (C = C), 1470 (C–N), 1435 (C–O), 847 (C–Br). ^1^H NMR 500 MHz, CDCl_3_) *δ*_H_ (ppm): 8.37 (s, 1H, H-5-quinazoline), 7.83 (d, 1H, *J* = 5 Hz, H-7-quinazoline), 7.61 (d, 2H, *J* = 5 Hz, phenyl), 7.49 (d, 1H, *J* = 5 Hz, H-8-quinazoline), 7.34 (d, 2H, *J* = 5 Hz, phenyl), 6.03 (s, 1H, uracil), 4.82 (s, 2H, quinazoline-CH_2_-uracil), 3.34 (s, 3H, CH_3_). ^13^C NMR (125 MHz, CDCl_3_) *δ*_C_ (ppm):160.70, 160.56, 151.10, 149.63, 145.60, 145.40, 137.99, 136.56, 133.64, 130.89, 129.66, 129.54, 129.42, 122.29, 121.17, 102.40, 47.67, 28.47. MS (*m/z*, %):508.1 (M^+^, 13.47), 473.1 (100), 415.1 (26.23), 394.1 (0.63), 233.2 (32.79).

*1-((6-Bromo-3-(4-Bromophenyl)-4-Oxo-3,4-Dihydroquinazolin-2-Yl)Methyl)-6-Chloro-3-Methylpyrimidine-2,4(1H,3H)-Dione (6h).*IR (KBr, cm^−1^): 2959 (CH), 2932 (CH), 1715 (C = O), 1674 (C = O), 1659 (C = O), 1607 (C = N), 1578 (C = C), 1470 (C–N), 1437 (C–O), 833 (C–Br). ^1^H NMR (500 MHz, CDCl_3_) *δ*_H_ (ppm): 8.35 (s, 1H, H-5-quinazoline), 7.82 (d, 1H, *J* = 10.Hz, H-7-quinazoline), 7.76 (d, 2H, *J* = 10 Hz, phenyl), 7.48 (d, 1H, *J* = 10 Hz, H-8-quinazoline), 7.27 (d, 2H, *J* = 10 Hz, phenyl), 6.01 (s, 1H, uracil), 4.81 (s, 2H, quinazoline-CH_2_-uracil), 3.33 (s, 3H, CH_3_). ^13^C NMR (125 MHz, CDCl_3_) *δ*_C_ (ppm): 160.68, 160.48, 151.08, 149.56, 145.58, 145.38, 137.98, 134.17, 133.87, 129.69, 129.65, 129.52, 124.63, 122.26, 121.16, 102.38, 47.66, 28.46. MS (*m/z*, %): 551.9 (M^+^, 13.14), 517.0 (100), 457.0 (42.69), 391.9 (16.34), 314.0 (22.34), 233.1 (23.81), 170.0 (13.71).

*1-((6-Bromo-4-Oxo-3-(P-Tolyl)-3,4-Dihydroquinazolin-2-Yl)Methyl)-6-Chloro-3-Methylpyrimidine-2,4(1H,3H)-Dione (6i).* IR (KBr, cm^−1^): 3065–3046 (C–H), 2963–2924 (C–H), 1709 (C = O), 1682 (C = O), 1688 (C = O), 1605 (C = N), 1510 (C = C), 1333 (C–N), 1395 (C–O), 762 (C–Br).^1^H NMR (500 MHz, CDCl_3_) *δ*_H_ (ppm): 8.38 (s, 1H, H-5-quinazoline), 7.81 (d, 1H, *J* = 5 Hz, H-7-quinazoline), 7.47 (d,1H, *J* = 5 Hz,H-8-quinazoline), 7.41 (d, 2H, *J* = 5 Hz, phenyl), 7.24 (d, 2H, *J* = 5 Hz, phenyl), 6.01 (s, 1H, uracil), 4.83 (s, 2H, quinazoline-CH_2_-uracil), 3.34 (s, 3H, CH_3_), 2.47 (s, 3H, CH_3_ phenyl). ^13^C NMR (125 MHz, CDCl_3_) *δ*_C_ (ppm): 160.78, 160.77, 151.14, 150.19, 145.73, 145.56, 140.56, 137.74, 132.49, 131.23, 129.54, 127.57, 122.49, 120.88, 102.29, 100.76, 47.74, 28.45, 21.32. MS (*m/z*, %): 488.1 (M + 1^+^, 15.04), 453.1 (100), 394.1 (22.57), 327.0 (33.98), 233.1 (15.31), 146.1 (26.51), 170.0 (2.53), 75.1 (4.37).

*1-((6-Bromo-3-(4-Methoxyphenyl)-4-Oxo-3,4-Dihydroquinazolin-2-Yl)Methyl)-6-Chloro-3-Methylpyrimidine-2,4(1H,3H)-Dione (6j).* IR (KBr, cm^−1^): 3011 (CH), 2957(CH), 1704 (C = O), 1682 (C = O), 1668 (C = O), 1611 (C = N), 1437 (C = C), 1431(C–N), 1422 (C–O), 837 (C–Br) .^1^H NMR (500 MHz, CDCl_3_) *δ*_H_ (ppm): 8.35 (s, 1H, H-5-quinazoline), 7.78 (dd, 1H, *J* = 10 Hz, *J* = 5Hz, H-7-quinazoline), 7.44 (d, 1H, *J* = 15 Hz, H-8-quinazoline), 7.47 (d, 1H, *J* = 5 Hz, phenyl), 7.08 (d, 1H, *J* = 10 Hz, phenyl), 5.98 (s, 1H, uracil), 4.81 (s, 2H, quinazoline-CH_2_-uracil), 3.86 (s, 3H, CH_3_), 3.31 (s, 3H, OCH_3_). ^13^C NMR (125 MHz, CDCl_3_) *δ*_C_ (ppm): 160.99, 160.83, 160.73, 151.19, 150.46, 145.75, 145.59, 137.78, 129.64, 129.58, 128.98, 127.47, 122.50, 120.91, 115.82, 102.34, 55.68, 47.78, 28.49. MS (*m/z*, %): 504.0 (M + 1^+^, 19.33), 469.0 (100), 411.0 (26.62), 343.0 (32.78), 249.1 (12.35), 221.0 (9.19).

*1-((6-Bromo-4-Oxo-3-(4-Phenoxyphenyl)-3,4-Dihydroquinazolin-2-Yl)Methyl)-6-Chloro-3-Methylpyrimidine-2,4(1H,3H)-Dione (6k).* IR (KBr, cm^−1^): 2963 (CH), 2826 (CH), 1719 (C = O), 1684 (C = O), 1676 (C = O), 1609 (C = N), 1504 (C = C), 1487 (C–N), 1437 (C–O), 847 (C–Br). ^1^H NMR (500 MHz, CDCl_3_) *δ*_H_ (ppm): 8.38 (s, 1H, H-5-quinazoline), 7.82 (d, 1H, *J* = 10 Hz, H-7-quinazoline), 7.48 (d, 1H, *J* = 10 Hz, H-8-quinazoline), 7.40–7.43 (m, 2H, phenyl), 7.30 (d, 2H, *J* = 10 Hz, phenyl), 7.20–7.23 (m, 1H, *J* = 5 Hz, phenyl), 7.17 (d, 2H, *J* = 5 Hz, phenyl), 7.12 (d, 2H, *J* = 5 Hz, phenyl), 6.02 (s, 1H, uracil), 4.86 (s, 2H, quinazoline-CH_2_-uracil), 3.34 (s, 3H, CH_3_). ^13^C NMR (125 MHz, CDCl_3_) *δ*_C_ (ppm): 160.85, 160.77, 159.34, 155.53, 150.22, 145.70, 145.53, 137.84, 130.14, 129.63, 129.56, 129.30, 129.21, 124.71, 122.45, 120.99, 120.20, 119.36, 102.35, 47.77, 28.47. MS (*m/z*, %): 566.2 (M^+^, 14.06), 530.2 (100), 473.2 (98.51), 392.2 (1264), 312.1 (18.20).

*1-((6-Bromo-3-(2,4-Dimethoxyphenyl)-4-Oxo-3,4-Dihydroquinazolin-2-Yl)Methyl)-6-Chloro-3-Methylpyrimidine-2,4(1H,3H)-Dione (6l).* IR (KBr, cm^−1^): 2940 (CH), 2841 (CH), 1705 (C = O), 1695 (C = O), 1624 (C = O), 1605 (C = N), 1587 (C = C), 1327(C–N), 1418 (C–O), 837 (C–Br). ^1^H NMR (500 MHz, CDCl_3_) *δ*_H_ (ppm): 8.41 (s, 1H, H-5-quinazoline), 7.84 (d, 1H, *J* = 10 Hz, H-7-quinazoline), 7.52 (d, 1H, *J* = 10 H-8-quinazoline), 7.16 (d, 1H, *J* = 5 Hz, phenyl), 6.61 (s, 1H, phenyl), 6.58 (d, 1H, *J* = 5 Hz, phenyl), 6.14 (s, 1H, uracil), 5.24 (d, 1H, *J* = 10 Hz, quinazoline-CH_2_-uracil), 5.08 (d, 1H, *J* = 15 Hz, quinazoline-CH_2_-uracil_2_), 3.85 (s, 3H, OCH_3_), 3.81 (s, 3H, OCH_3_), 3.45 (s, 3H, CH_3_). ^13^C NMR (126 MHz, CDCl_3_) *δ*_C_ (ppm): 162.23, 161.93, 160.58, 156.40, 155.47, 155.35, 151.27, 145.82, 137.71, 129.95, 129.77, 129.37, 122.83, 121.02, 116.00, 106.36, 105.30, 99.94, 66.40, 55.93, 55.67, 28.16. MS (*m/z*, %): 534.0 (M + 1^+^, 14.08), 348 (100), 374.0 (39.64), 359.0 (44.98), 343.0 (100), 315.0 (11.25), 220.1 (5.85), 153.1 (19.18).

*1-((6-Bromo-3-(3,4-Dimethylphenyl)-4-Oxo-3,4-Dihydroquinazolin-2-Yl)Methyl)-6-Chloro-3-Methylpyrimidine-2,4(1H,3H)-Dione (6m).* IR (KBr, cm^−1^): 2965–2918 (C–H), 2858 (C–H), 1713 (C = O), 1686 (C = O), 1672 (C = O), 1602 (C = N), 1503 (C = C), 1275 (C–N), 1395 (C–O), 762 (C–Br, stretch). ^1^H NMR (500 MHz, CDCl_3_) *δ*_H_ (ppm): 8.38 (s, 1H, H-5-quinazoline), 7.80 (d,1H, *J* = 5 Hz, H-7-quinazoline), 7.47 (d, 1H, *J* = 10 Hz, phenyl), 7.36 (d, 1H, *J* = 10 Hz, H-8-quinazoline), 7.12 (s, 1H, phenyl), 7.08 (d, 1H, *J* = 10 Hz , phenyl), 6.00 (s, 1H, uracil), 4.85(s, 2H, quinazoline-CH_2_-uracil), 3.34 (s, 3H, CH_3_), 2.34 (s, 3H, CH_3_-phenyl), 2.35 (s, 3H, CH_3_-phenyl). ^13^C NMR (125 MHz, CDCl_3_) *δ*_*C*_ (ppm): 160.81, 151.17, 150.26, 145.76, 145.60, 139.42, 139.26, 137.70, 132.67, 131.61, 129.59, 129.55, 128.50, 124.89, 122.52, 120.83, 102.29, 47.79, 29.69, 28.47, 20.00, 19.65. MS (*m/z*, %):502.1 (M + 1^+^, 14.43), 467.1 (100), 409.1 (20.61), 366.0 (9.48), 341.1 (40.57), 247.1 (16.68), 221.0 (4.81), 116.1 (17.35).

*1-((6-Bromo-3-(3-Chloro-4-Fluorophenyl)-4-Oxo-3,4-Dihydroquinazolin-2-Yl)Methyl)-6-Chloro-3-Methylpyrimidine-2,4(1H,3H)-Dione (6n).* IR (KBr, cm^−1^): 3071–3038 (C–H), 2959 (C–H), 1713 (C = O), 1678 (C = O), 1682 (C = O), 1611 (C = N), 1503 (C = C), 1267 (C–N), 1395 (C–O), 835 (C–Br, stretch). ^1^H NMR (500 MHz, CDCl_3_) *δ*_H_ (ppm): 8.29 (d, 1H, *J* = 5, H-5-quinazoline), 7.76 (dd, 1H, *J* = 10 Hz, *J* = 5 Hz, H-7-quinazoline), 7.41–743 (m, 2H, phenyl), 7.34 (t, 1H, *J* = 10 Hz, H-8-quinazoline) 7.22–7.26 (m, 1H, phenyl), 5.96 (s, 1H, uracil), 4.77 (s, 2H, quinazoline-CH_2_-uracil), 3.27 (s, 3H, CH_3_). ^13^C NMR (125 MHz, CDCl3) *δ*_C_ (ppm): 160.70, 157.82, 151.12, 149.42, 145.51, 145.35, 138.20, 131.54, 130.67, 129.74, 128.26, 123.56, 123.37, 122.18, 121.38, 118.63, 118.41, 102.48, 47.64, 28.51. MS (*m/z*, %): 526.0 (M^+^, 14.08), 491.0 (100), 434.0 (26.32), 366.0 (27.78), 251.1 (28.00), 223.0 (20.49), 221.0 (4.81), 116.1 (10.24).

### Cell culture assay

Three human cancer cell lines with origin of breast (MCF-7), lung (A549), and colorectal cancer (SW-480) were obtained from the National Cell Bank, Pasteur Institute, Tehran, Iran. The cells were cultured in 1640 RPMI medium (Bio Idea, Iran), 10% fetal bovine serum (FBS; Gibco, USA) and 1% penicillin–streptomycin (Biosera, France) and were maintained at 37 °C in a 5% CO_2_ incubator. The cell lines were separately seeded in 96-well plates at a density of 8000 cells/well and were kept for 24 h to reattach. The cells were then treated with 6 different concentrations of the synthesized compounds and further incubated for 72 h. Then, the media were removed and replaced with 100 μL of MTT solution (0.5 mg/mL) was added and incubated for another 4 h. To dissolve the purple formazan crystals, 100 μL dimethyl sulfoxide (DMSO) (Sigma-Aldrich, Germany) was added to each well and left for 30 min. Finally, the absorbance of each well was obtained at 570 nm using an ELISA plate reader (Biotek, Winooski, VT, USA). To obtain inhibitory index of each compound, first the cell viability was normalized to the untreated control and then, a plot of inhibition index versus concentration was depicted. Curve Expert software version 1.40 was used to calculate the inhibitory concentration 50 (IC_50_) values.

### Apoptosis analysis

The AnnexinV/PI detection kit was applied to assess the ability of compound *6n* with the lowest IC_50_ in the induction of apoptosis in A549 cell line. To do this, 1 × 10^5^ cells in 500 μL complete culture media were cultured for overnight followed by exposure to compound *6n* for 72 h. After the incubation time, AnnexinV/PI staining using eBioscience™ Annexin V apoptosis detection kit (Invitrogen). The cells were gently harvested and washed once with 1X phosphate buffered saline (PBS), and once with 1000 μL 1X binding buffer. In the next step, the cells were suspended in 100 μL binding buffer containing 5 μL fluorescein isothiocyanate conjugated (FITC) Annexin V for 15 min and 5 μL Propodium Iodide (PI) solution. The cells were then acquired on BD FACS Calibur™ flow cytometry (BD Biosciences, San Jose, CA, USA) and apoptosis rates were calculated as the sum of early apoptosis and late apoptosis using Flowjo software.

### Cell cycle analysis

The effect of 6n was also assessed according to our previous protocol^23^. Briefly, 1 × 10^5^ A549 cells were seeded in a 24-well plate in 500 mL complete cell culture media and after 24 h, were treated with two concentrations of 6n (10 and 15 μM). After 72 h, the cells were harvested and washed twice with cold 1X pbs and stabilized with cold ethanol (70%) in drop-wised manner while vortexing. After keeping one day in the freezer, the fixed cells were washed with 1X pbs and treated with a mixture of ribonuclease (10 µg/ml) (Sigma-Aldrich, Germany) to remove RNA, and Propidium Iodide (PI, 20 µg/ml) (Sigma-Aldrich, Germany) to stain DNA. The cells were acquired on BD FACS Calibur™ flow cytometry (BD Biosciences, San Jose, CA, USA). The data were analyzed with FlowJo software packages version 10.

### Statistical analysis

For each analysis, the data were presented as the mean SD. One-way ANOVA statistical analyses were carried out using GraphPad Prism 8.0 software (GraphPad Software Inc.).

### Docking procedure

Molecular docking procedure was carried out using AutoDock 4.0 software. The 3D EGFR complex structure was obtained from Protein Data Bank (PDB code: 1M17). ACD chem BioDraw Uitra13.0 was used to draw the structures, then the molecules were minimized by molecular mechanics (MM^+^) and semi-empirical (AM1) method utilizing hyperchem software^35^. In order to prepare the protein, protein's unwanted water and the cognate ligand were removed and the missing hydrogen atoms were added then merged nonpolar hydrogens based on their carbon atoms. AutoDock Vina in conjunction with a batch script (DOCKFACE) was used to dock a grid box with a size of 70*70*70. Then the centroid of x = 20.143, y = 0.376, z = 52.210 and docking parameters were set with the default settings. Discovery studio 2021 software was used to analyze binding interactions between docked compounds and receptors. Lastly, Discovery studio 2021 client software was used to generate the pictures.

### Molecular dynamics simulations

To generate full Amber starting files including the AMBERff14SB force field, the Ambertools package was used ^36^ for the protein, and the General Amber Force Field (GAFF)^37^ employing the AM1-BCC charge model was used for ligands^38^. The protonation states of all the titratable residues were set at their default values at pH 7.0 and the *N-* and C-termini of the receptor was acetylated and aminated, respectively. The TIP3P water model was presented to solvate the compounds and ions added to the box to accomplish a neutral system^39^. An electrolyte concentration of 150 mM KCl was added to the system to mimic physiological salt concentrations. The total size for each system was approximately 63,000 atoms.

### Simulation setup protocol

All MD simulations were performed using the GPU version of the PMEMD in the Amber22 software package^40^. The periodic boundary condition (PBC) was used in three dimensions. The sharpest descent algorithm was utilized to minimalize their energy before the MD simulation of protein − ligand complexes, and the leap-frog algorithm was accounted for to assimilate their motions. Similar to previous research, the Particle Mesh Ewald (PME) method was used within this procedure to perceive the influence of long-range molecules' electrostatic interfaces^41^. In addition, the LINCS algorithm was surveyed in both equilibration and production runs to mimic the constraining of the H-bonds in this procedure^42^. The nonbonded interaction cutoff was set at 12.0 Å. We used ensemble-based ESMACS (Enhanced sampling of molecular dynamics with the approximation of continuum solvent) protocol^43–45^. This procedure is based on the setting of independent MD simulations to achieve a good estimation for binding free energy and associated uncertainties^46–48^. In this regard, a set of 25 replicas for ESMACS calculation, with 4 ns production runs, were established in this study. For each replica, the energy system was minimized using a steepest descent algorithm for 200 steps followed by 7000 steps of conjugated gradient. This was followed by 10 ns of equilibration simulations in which the protein was restrained using a harmonic potential on all heavy protein atoms. These restraints were gradually lowered over 5 consecutive 2 ns simulations, employing force constants of 1000, 500, 200, 100, and 50 kJ mol^−1^ nm^−2^ and hearing each replica throughout 2 ns to 300 K during only the first two steps of equilibration procedure^.^ The same initial coordinates were used for a given ligand-receptor complex, with different initial velocities randomly assigned to the atoms according to a Maxwell–Boltzmann distribution.

### Free energy calculations

Free energy calculation was obtained by the ESMACS approach and the binding free energy (ΔG) of every trivial molecule as an inhibitor was achieved with Eq. 1.

1 $$\Delta G_{Bind} = \, G_{Com} - \, \left( {G_{{{\text{Re}} c}} + G_{Lig} } \right)$$

Com, Rec, and Lig subscripts are associated with the complex, receptor, and ligand, ESMACS approach is based on this equation, in which *G*_*i*_ is calculated from a set of structures from MD simulations. The molecular mechanics Poisson–Boltzmann surface area (MM/PBSA) and generalized Born Surface Area (MM/GBSA) as continuum solvation was applied for the binding free energy calculations of selected compounds to EGFR receptor. For ESMACS, the trajectories for each replica containing 200 snapshots were further analyzed by MMPBSA.py.MPI to extract the energetic information for each snapshot. A script was then run to aggregate these results from the ensemble of simulations and values of ΔG_ESMACS_ computed along with bootstrap statistics (Figure S1–S44).

### Hydrogen bonding, energy decomposition and clustering analysis

We used the H-bond module of AmberTools22 to consider a hydrogen bond formed between the chosen ligands and the protein (distance of 3.0 Å and an angle cutoff of 135). Therefore, all frames from all replicas has been used for this purpose. The residue energy decomposition reveals a congenial and displeasing data interface that might be used to improve lead quality. We evaluated the Water Swap residue-wise binding energy decompositions for the residues that made substantial influences on inhibitor binding during the MD simulation^49^. The most conventional and well-known similarity measure is the root mean square deviation (RMSD) values applied for partitioning MD trajectories that are gained by pairwise or matrix error distances^50^.

### Supplementary Information

Supplementary Information.

## Data Availability

The data sets used and analyzed during the current study are available from the corresponding author on reasonable request. We have presented all data in the form of Tables and Figure. The PDB code (1M17) was retrieved from protein data bank (www.rcsb.org). https://www.rcsb.org/structure/1M17.
