# Supplementary material for: Design, synthesis, computational study and cytotoxic evaluation of some new quinazoline derivatives containing pyrimidine moiety
Source: Sci Rep. 2023 Sep 2;13:14461. doi: 10.1038/s41598-023-41530-6 (PMC10475017; doi:10.1038/s41598-023-41530-6)
Supplement: Supplementary file 1 — Supplementary Information. [file 41598_2023_41530_MOESM1_ESM.docx]

**Design, synthesis, computational study and cytotoxic evaluation of some new quinazoline derivatives contain pyrimidine moiety**

Somayeh Zare^1^, Leila Emami^2^, Zahra Faghih^3^, Farshid Zargari^4,5^, Zeinab Faghih^2^, Soghra Khabnadideh^2^^[[1]](#footnote-1)^

*^1^School of Pharmacy, Shiraz University of Medical Sciences, Shiraz, Iran*

*^2^Pharmaceutical Sciences Research Center, Shiraz University of Medical Sciences, Shiraz, Iran*

*^3^Shiraz Institute for Cancer Research, Medical School, Shiraz University of Medical Sciences, Shiraz, Iran*

*^4^Pharmacology Research Center, Zahedan University of Medical Sciences, Zahedan, Iran*

*^5^Department of Chemistry, Faculty of Science, University of Sistan and Baluchestan (USB), Zahedan, Iran*


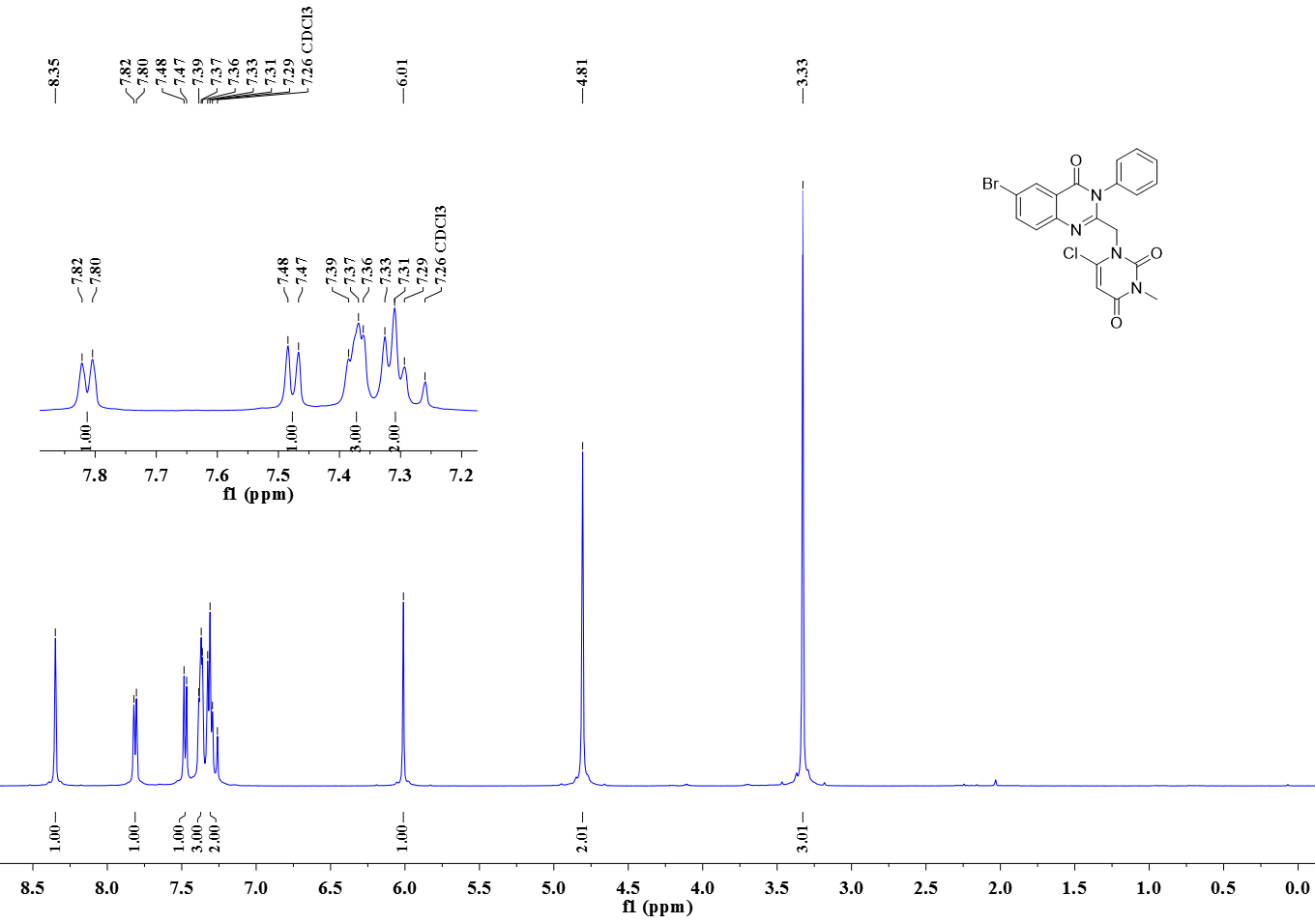


**Figure S3**. ^1^H-NMR spectrum of ***6a***

**
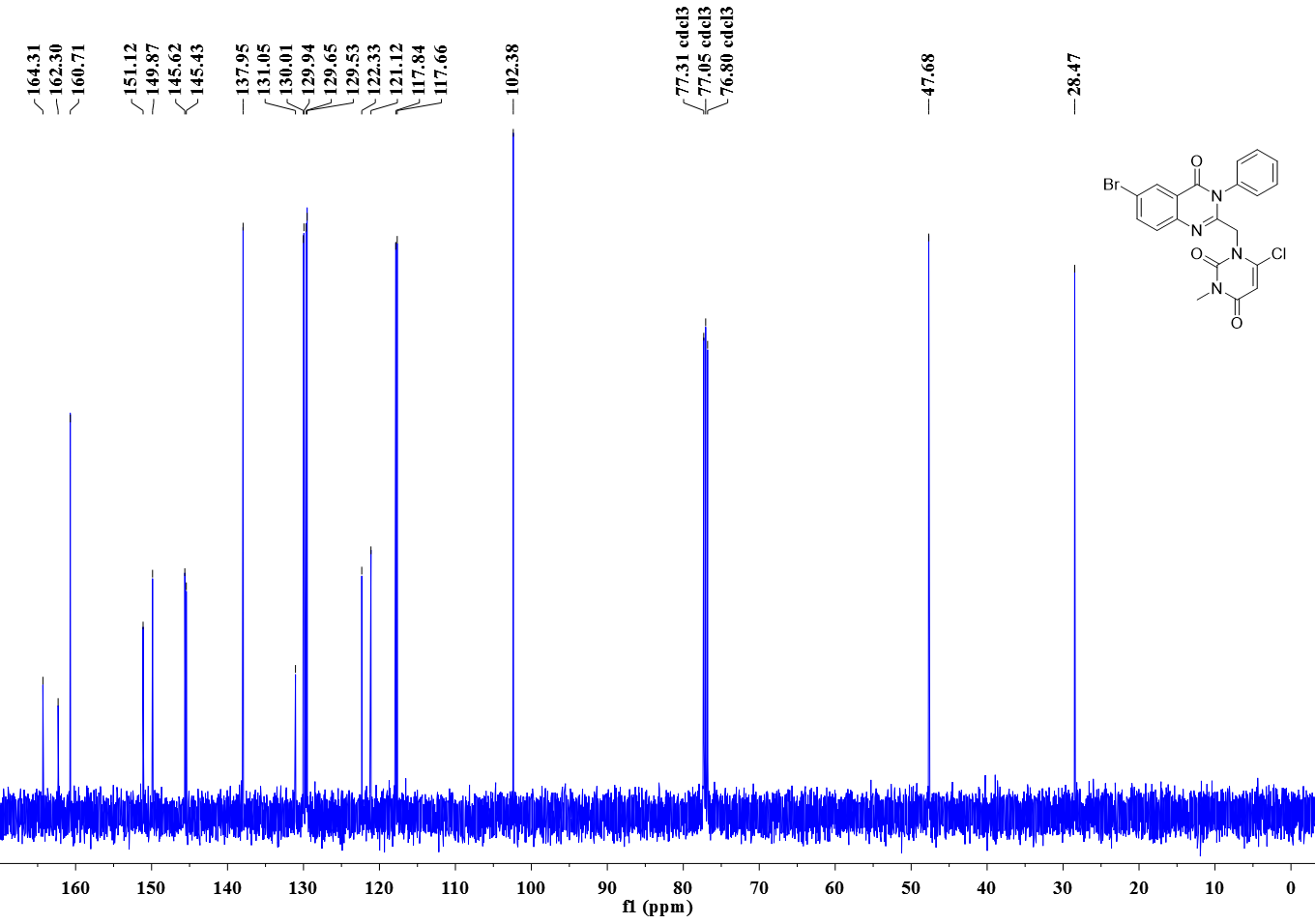
**

**Figure S4**. ^13^C-NMR spectrum of ***6a***


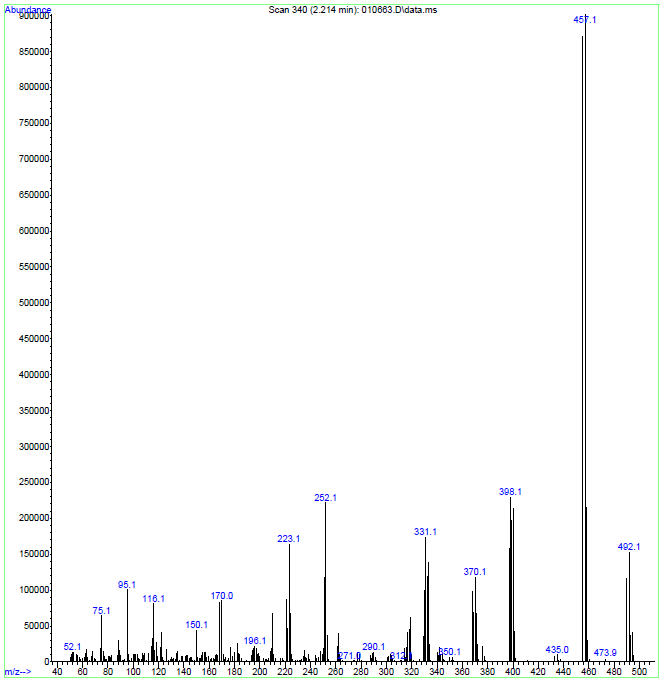


**Figure S5**. Mass spectrum of ***6a***

**
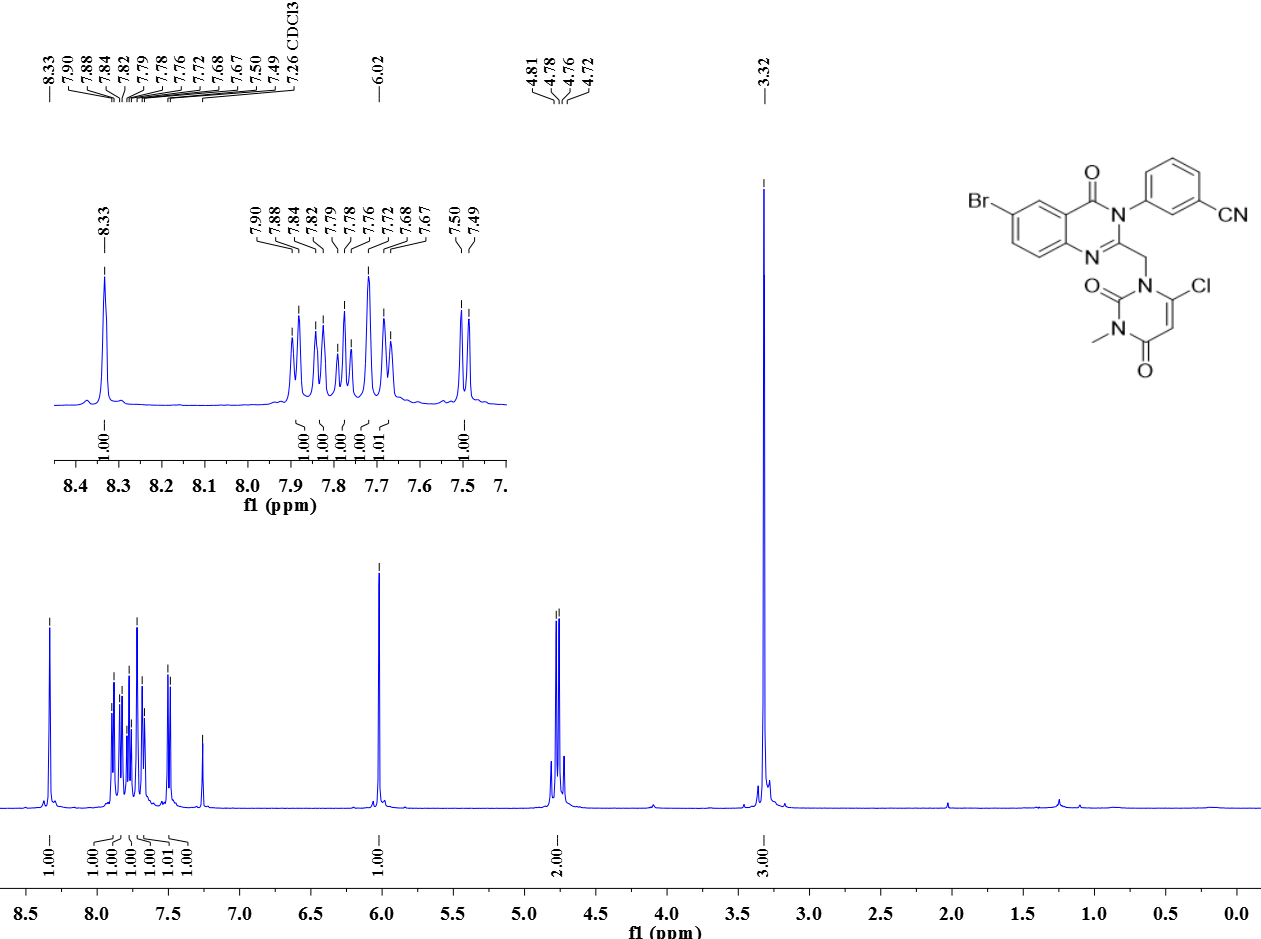
**

**Figure S6**. ^1^H-NMR spectrum of ***6b***

**
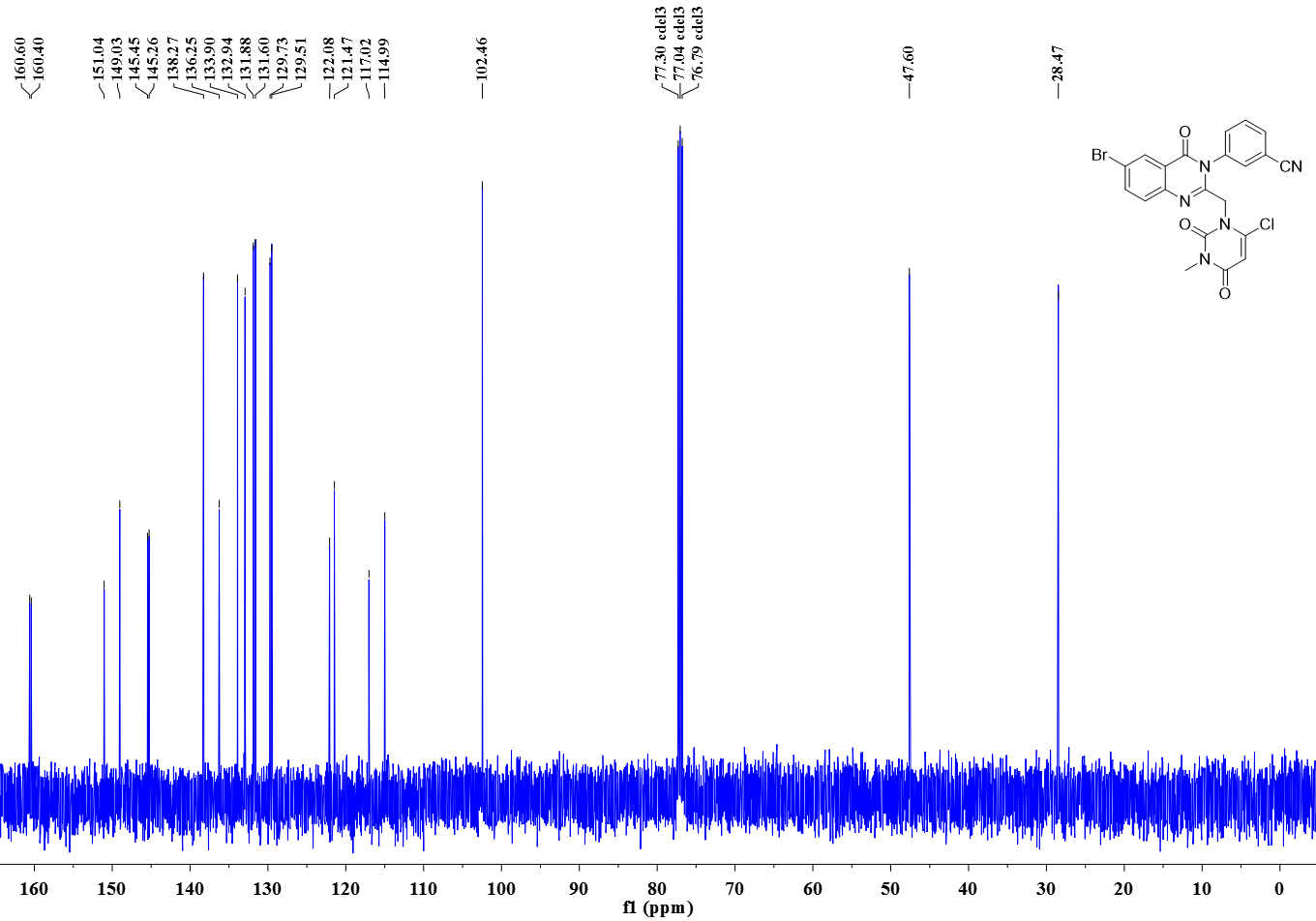
**

Figure S7. ^13^C-NMR spectrum of *6b*


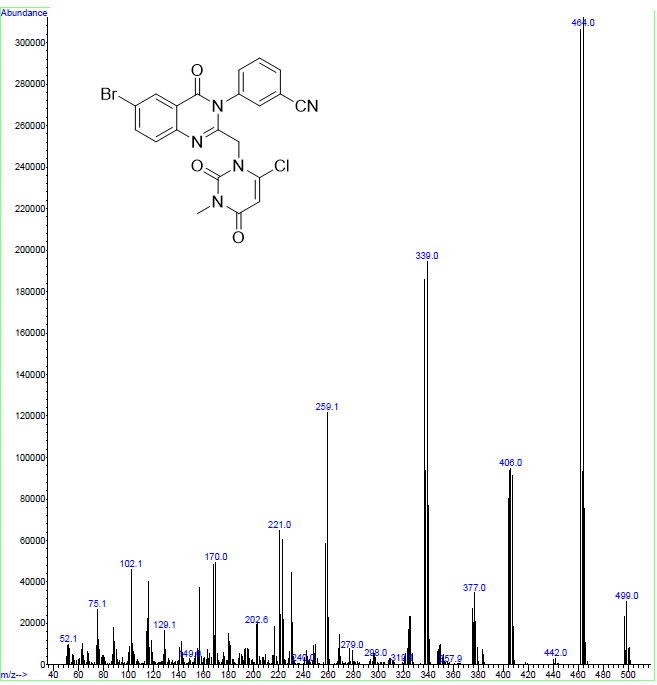


**Figure S8**. Mass spectrum of ***6b***


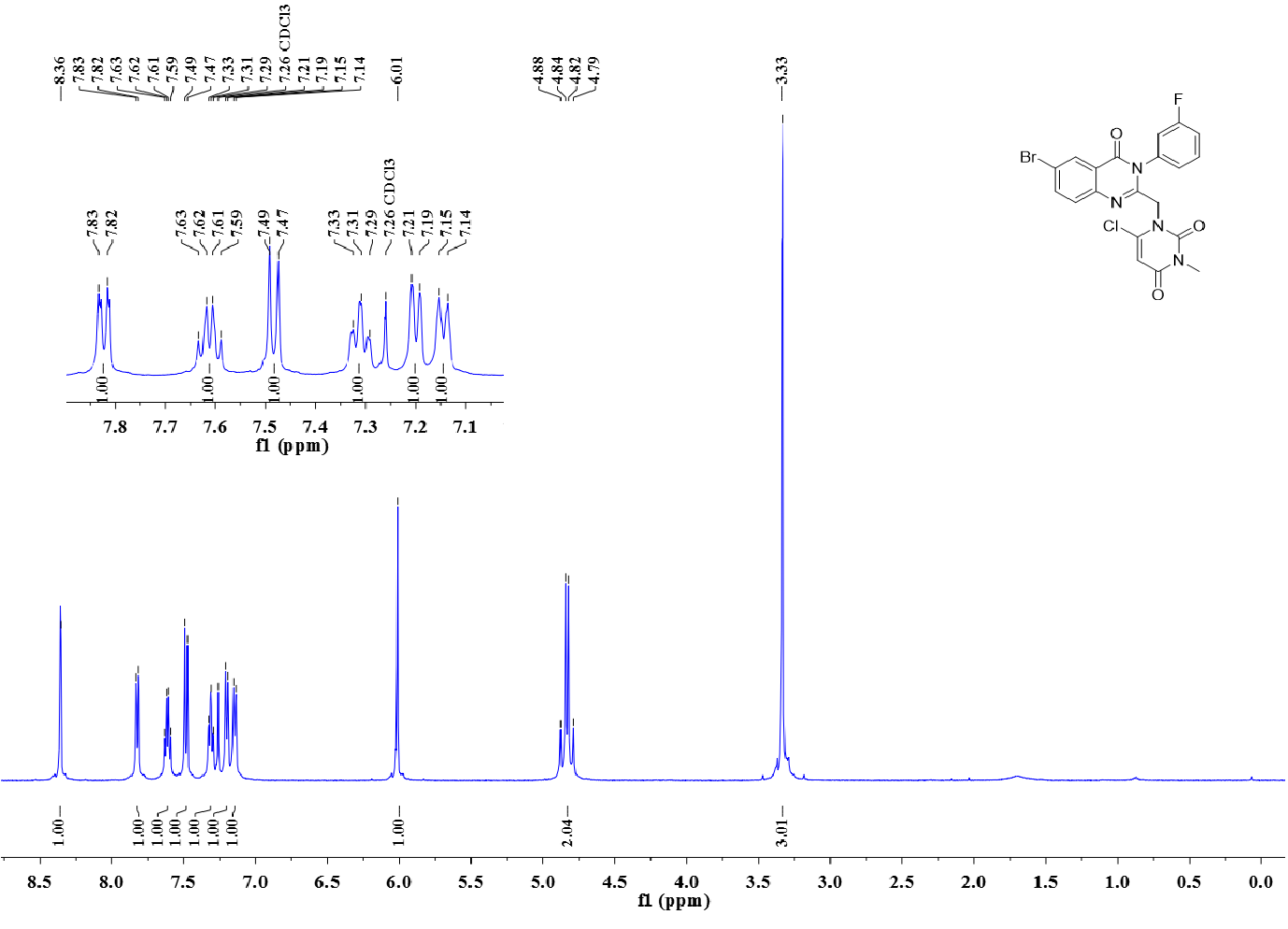


**Figure S9**. ^1^H-NMR spectrum of ***6c***

**
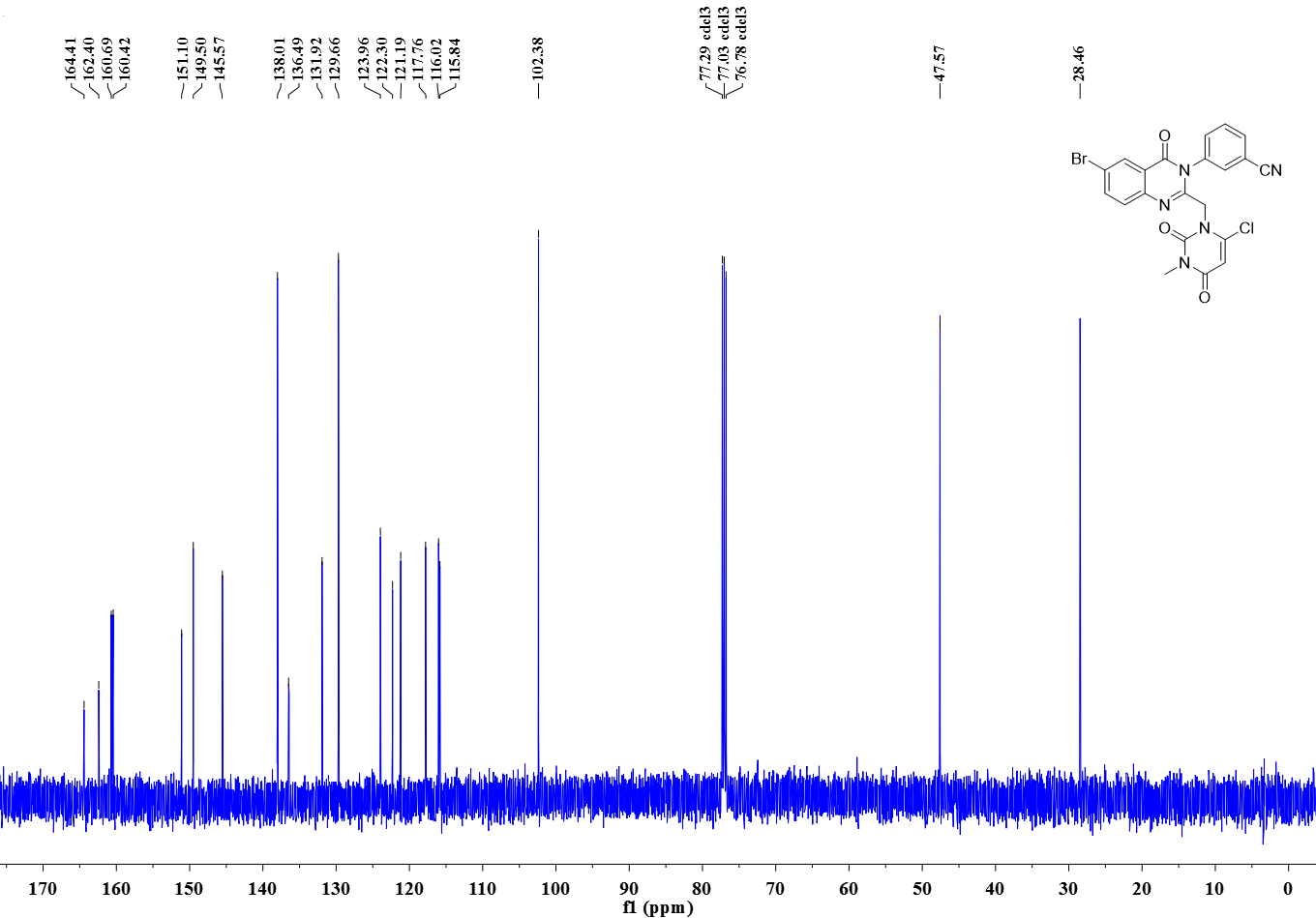
**

**Figure S10** ^13^C-NMR spectrum of ***6c***


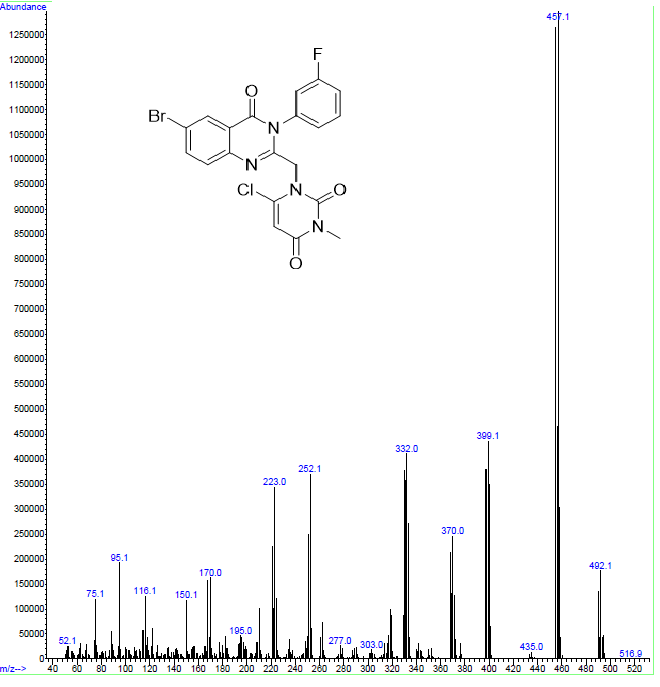


**Figure S11**. Mass spectrum of ***6c***


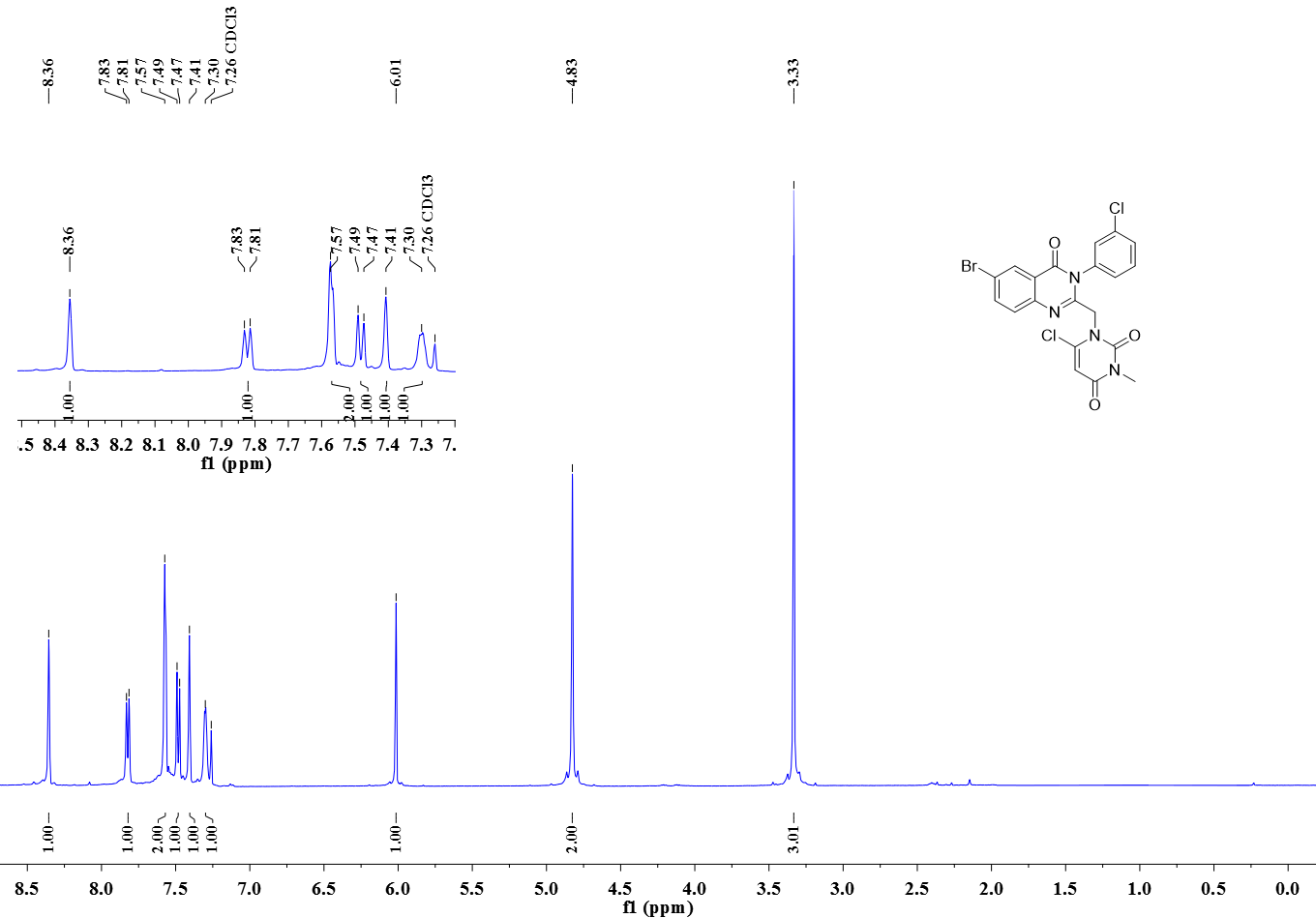


**Figure S12**. ^1^H-NMR spectrum of ***6d***

**
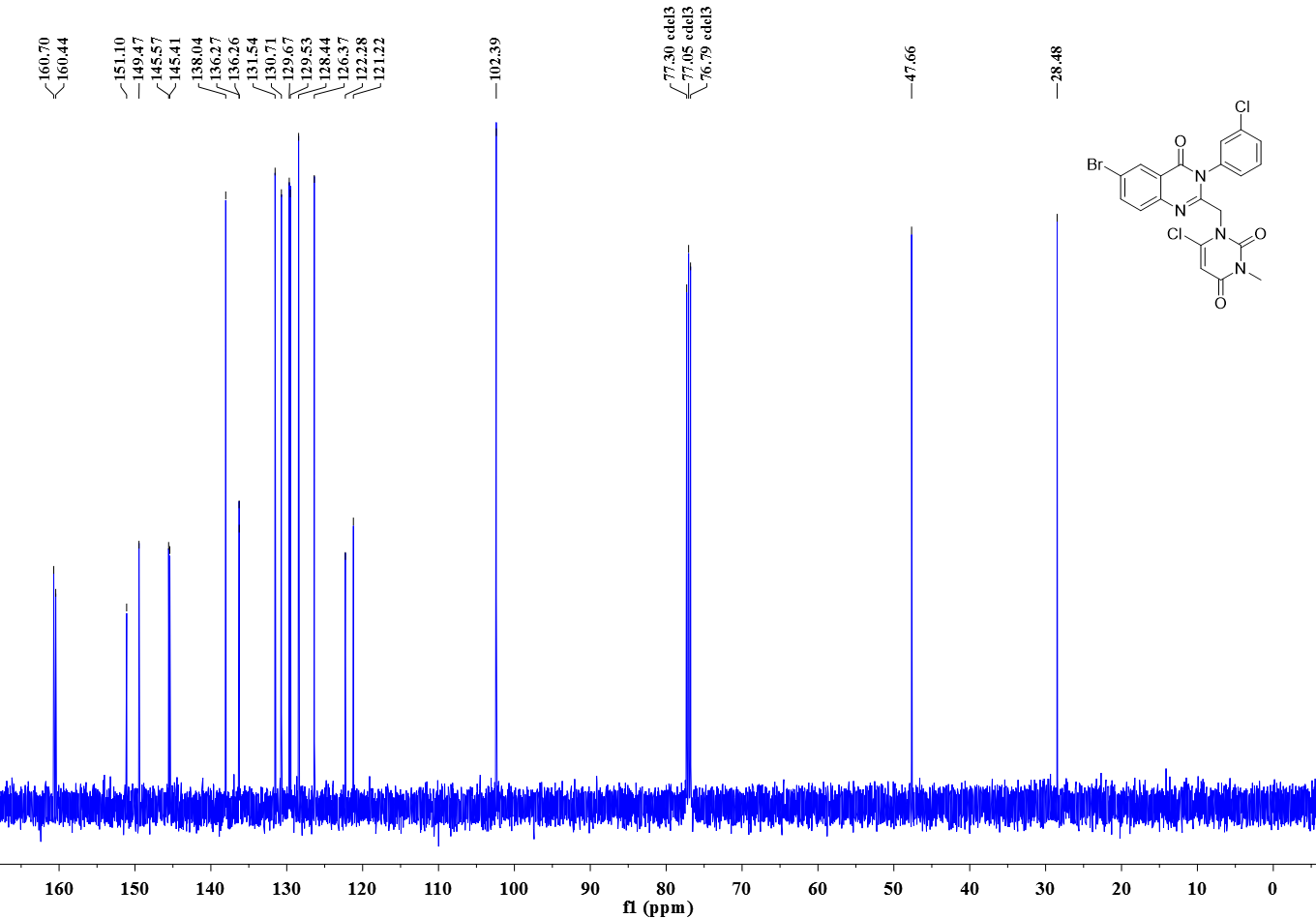
**

**Figure S13**. ^13^C-NMR spectrum of ***6d***


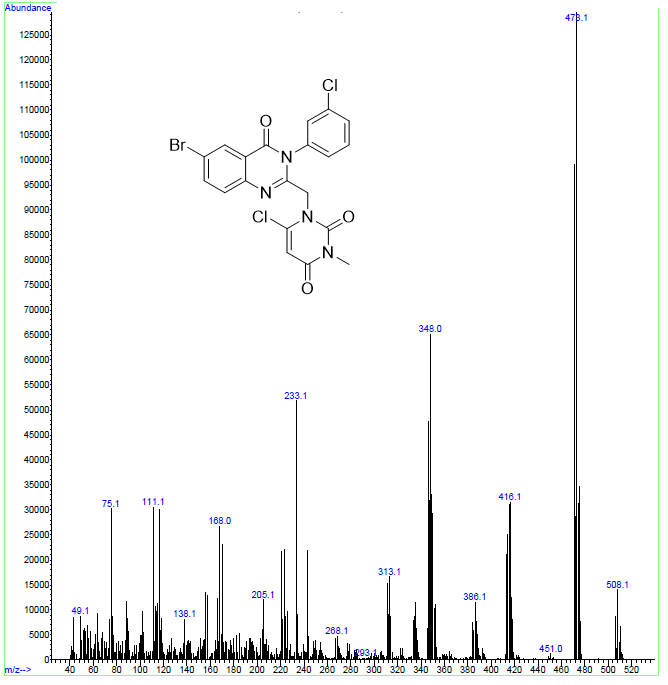


**Figure S14.** Mass spectrum of ***6d***

**
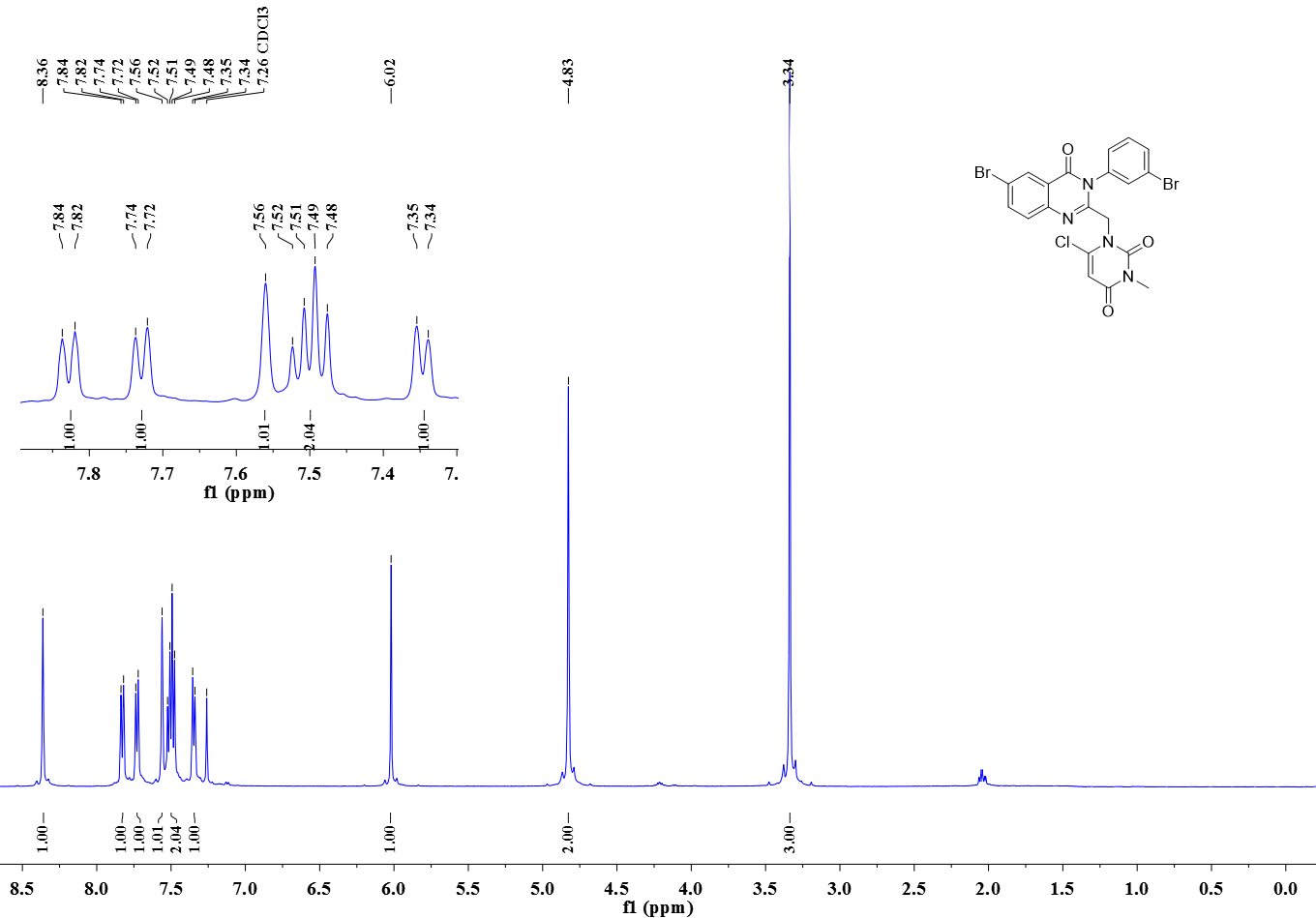
**

**Figure S15**. ^1^H-NMR spectrum of ***6e***

**
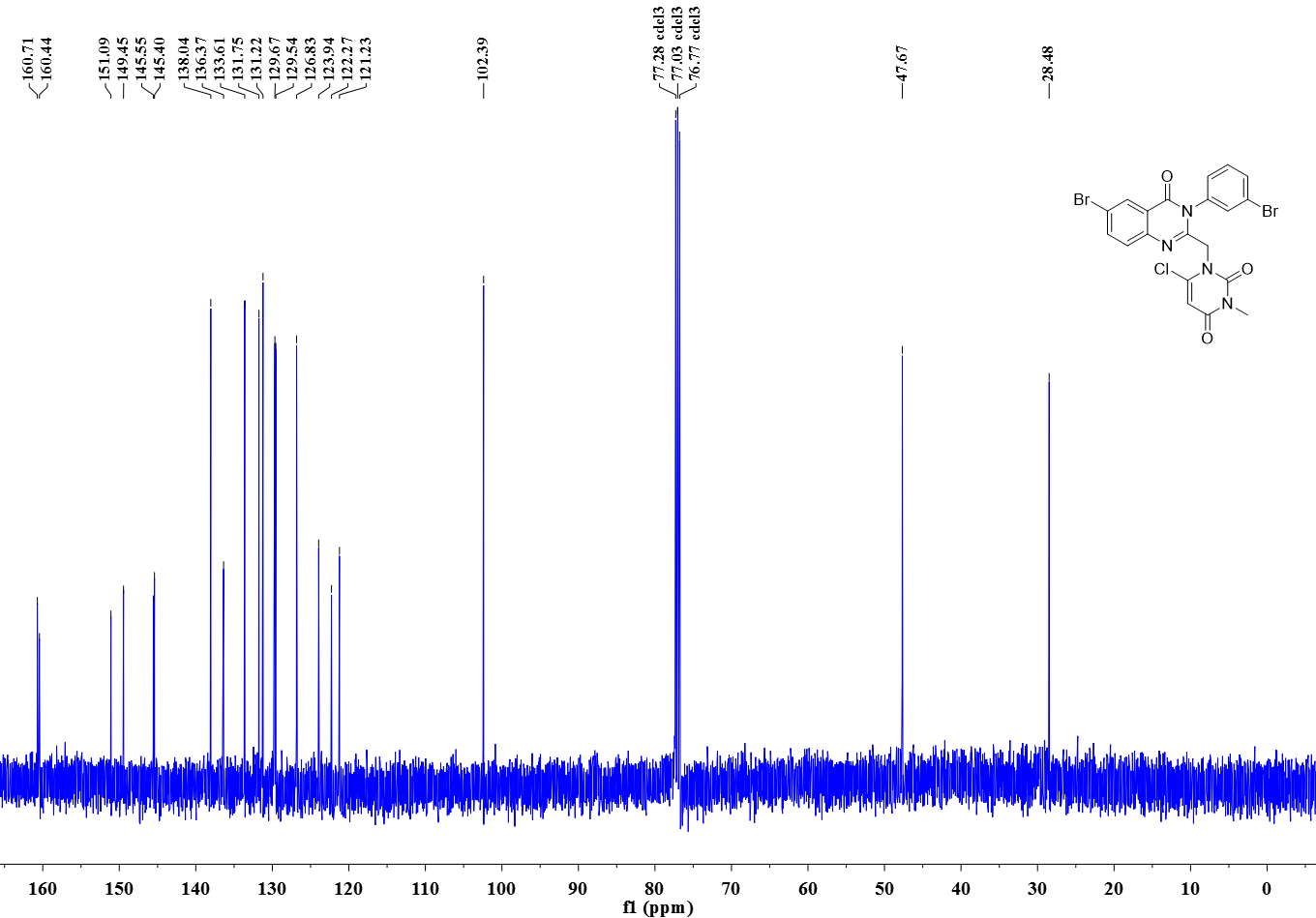
**

**Figure S16**. ^13^C-NMR spectrum of ***6e***

**
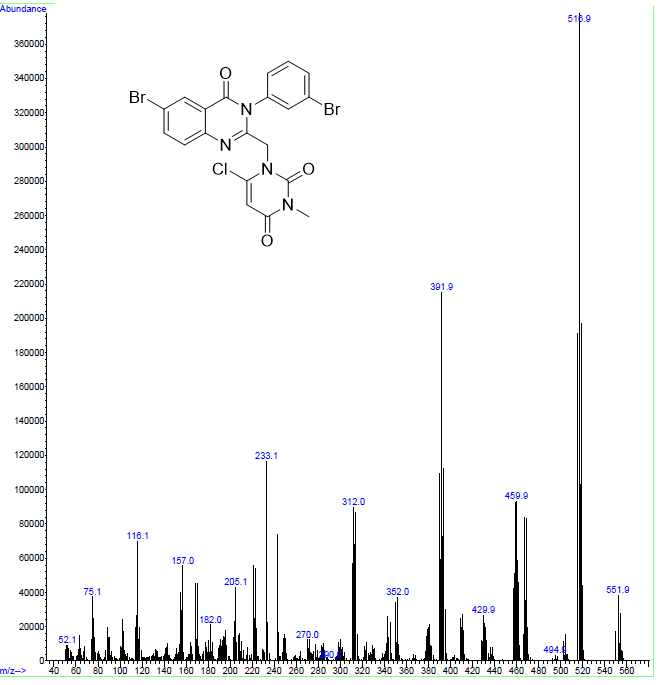
**

**Figure S17**. Mass spectrum of ***6e***

**
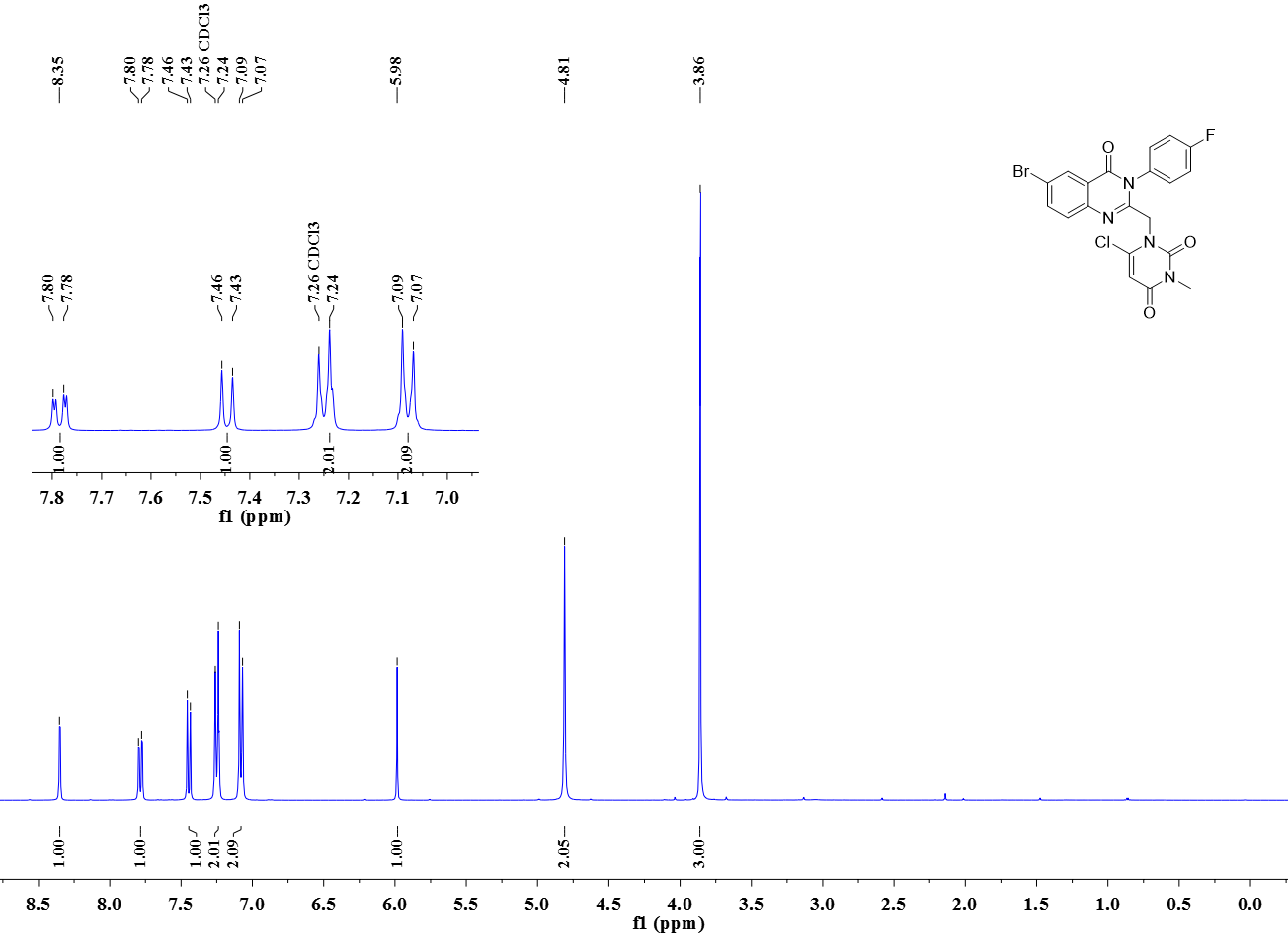
**

**Figure S18**. ^1^H-NMR spectrum of ***6f***

**_
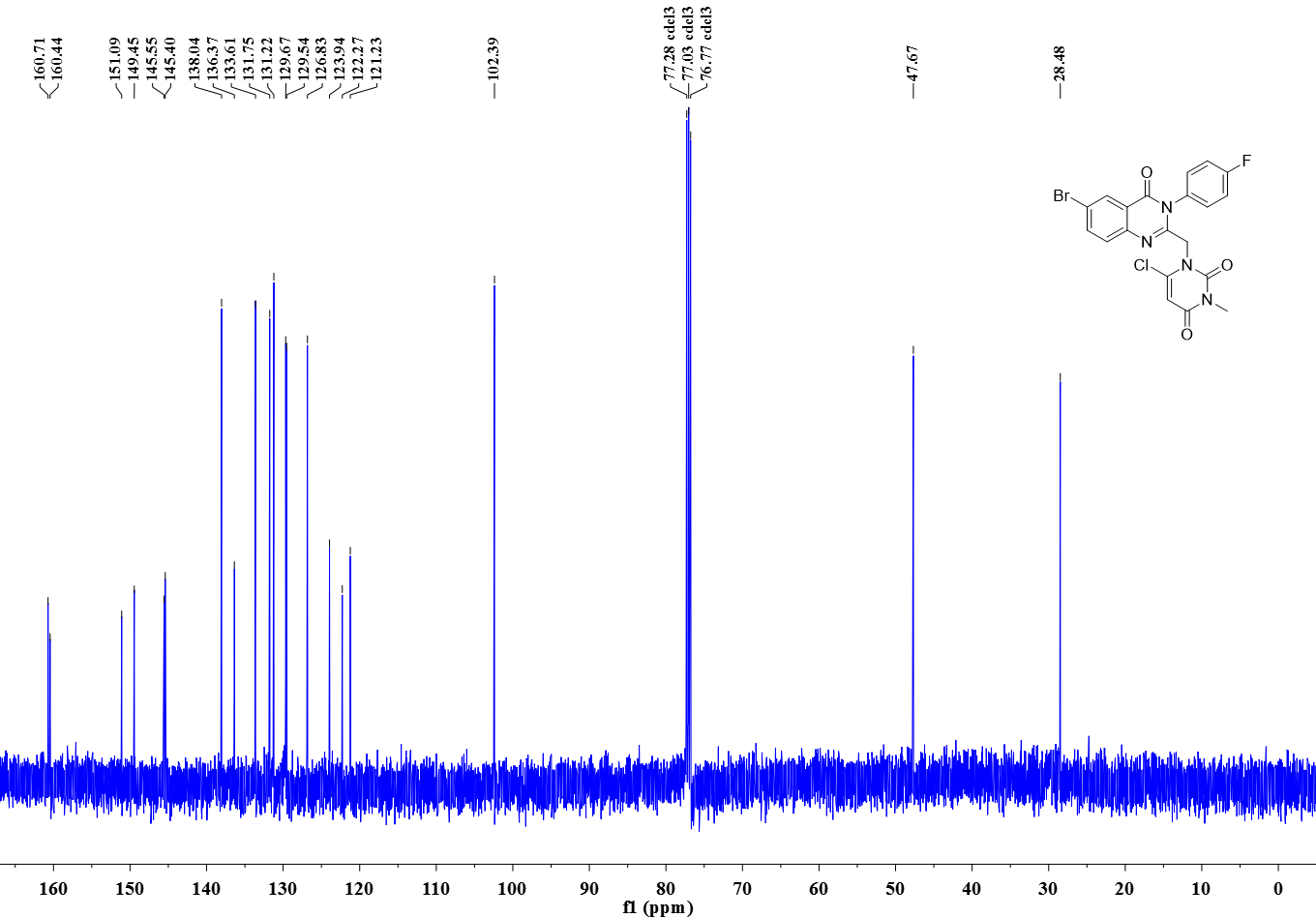
_**

**Figure S19**. ^13^C-NMR spectrum of ***6f***

.


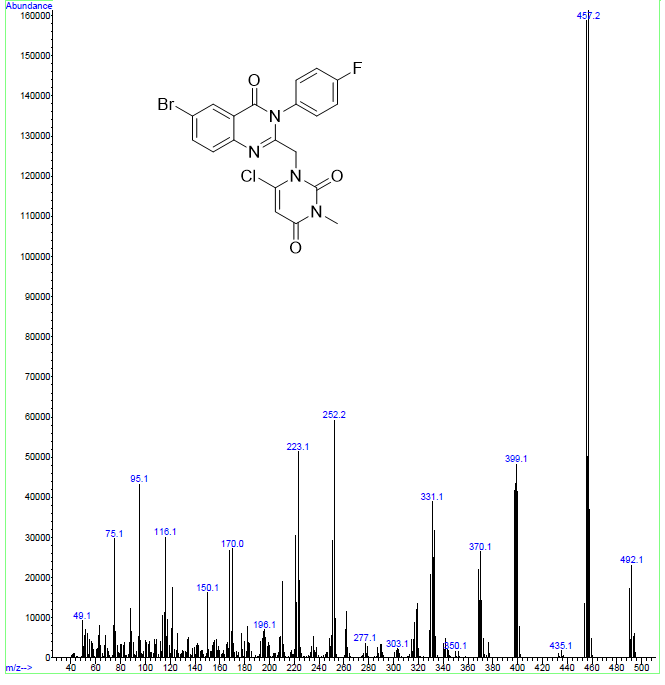


**Figure S20**. Mass spectrum of ***6f***

**
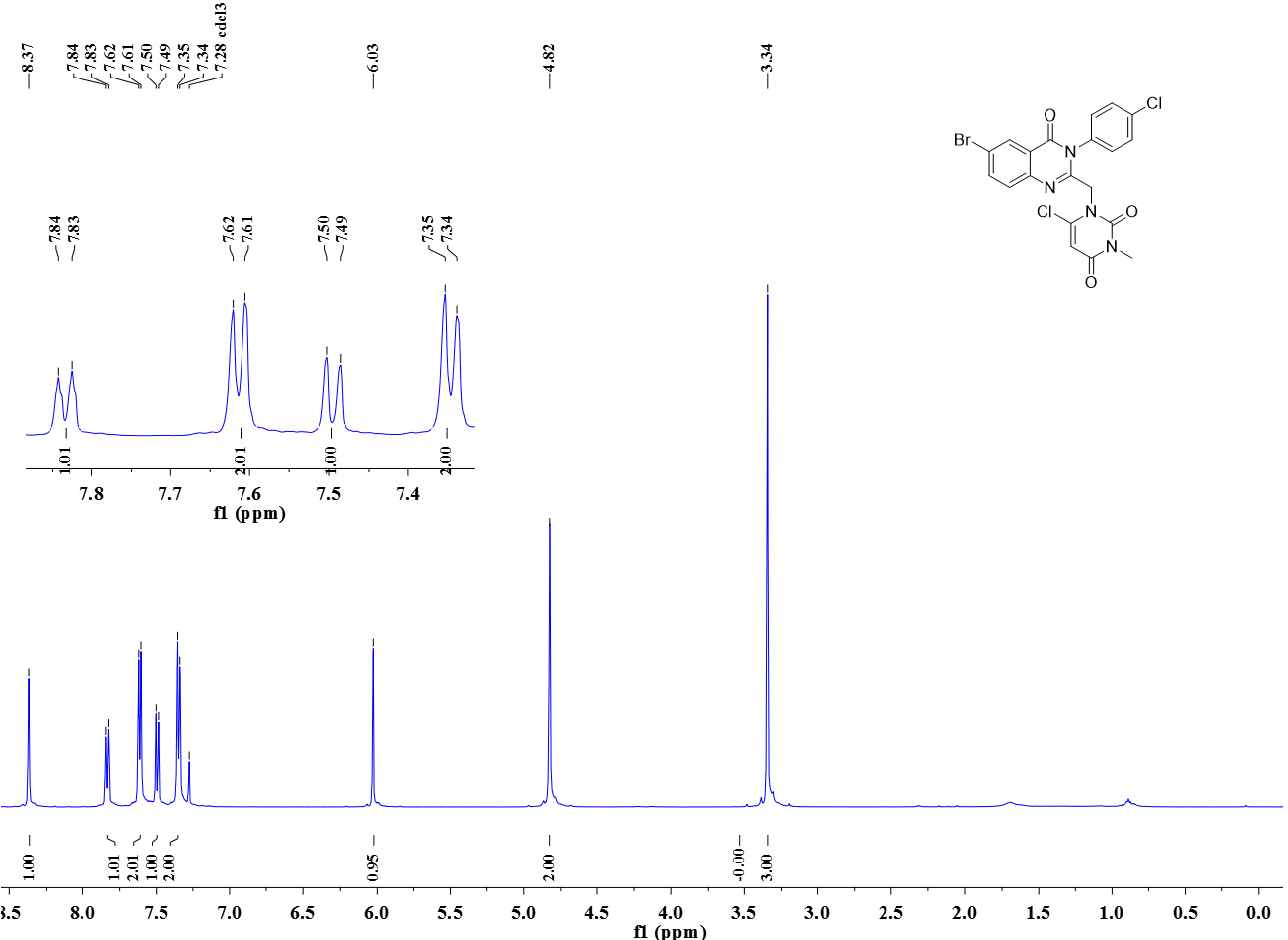
**

**Figure S21**. ^1^H-NMR spectrum of ***6g***

**
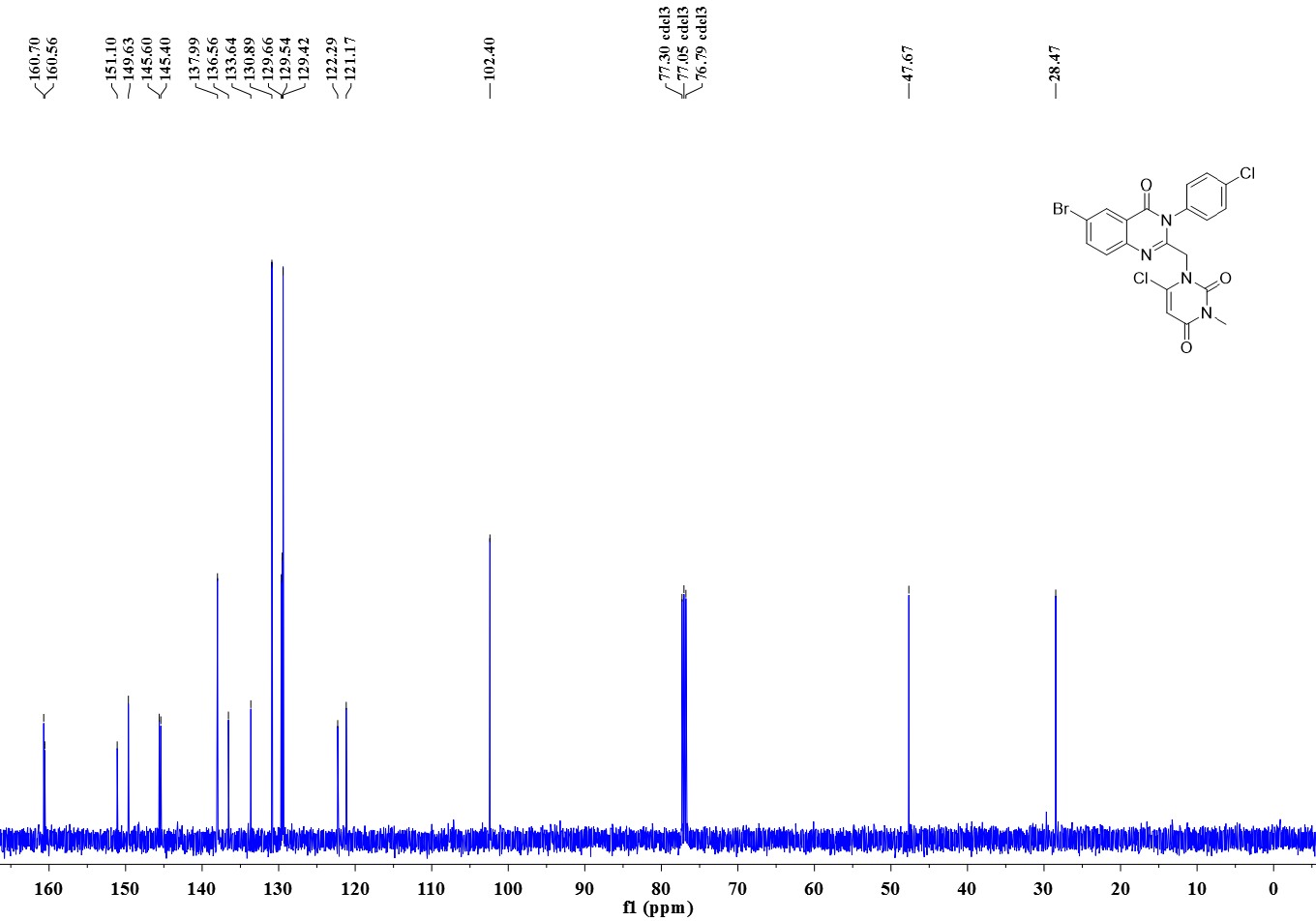
**

**Figure S22**. ^13^C-NMR spectrum of ***6g***

**
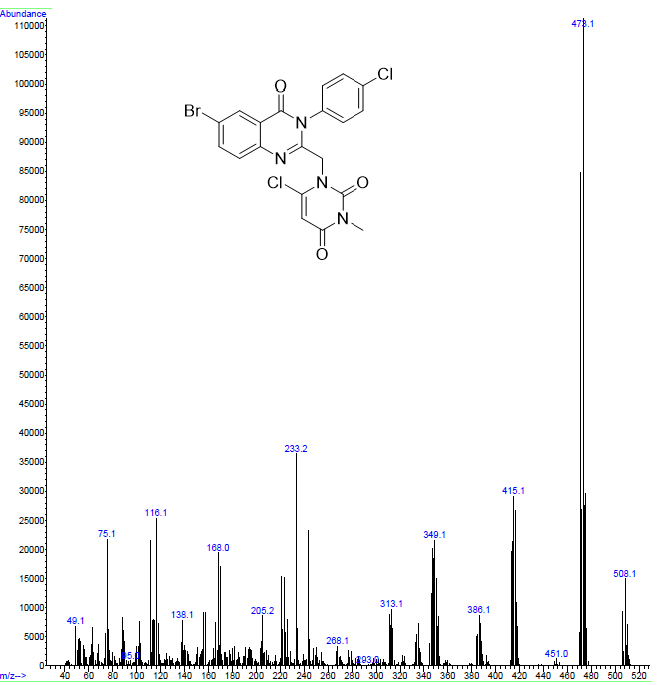
**

**Figure S23**. Mass spectrum of ***6g***


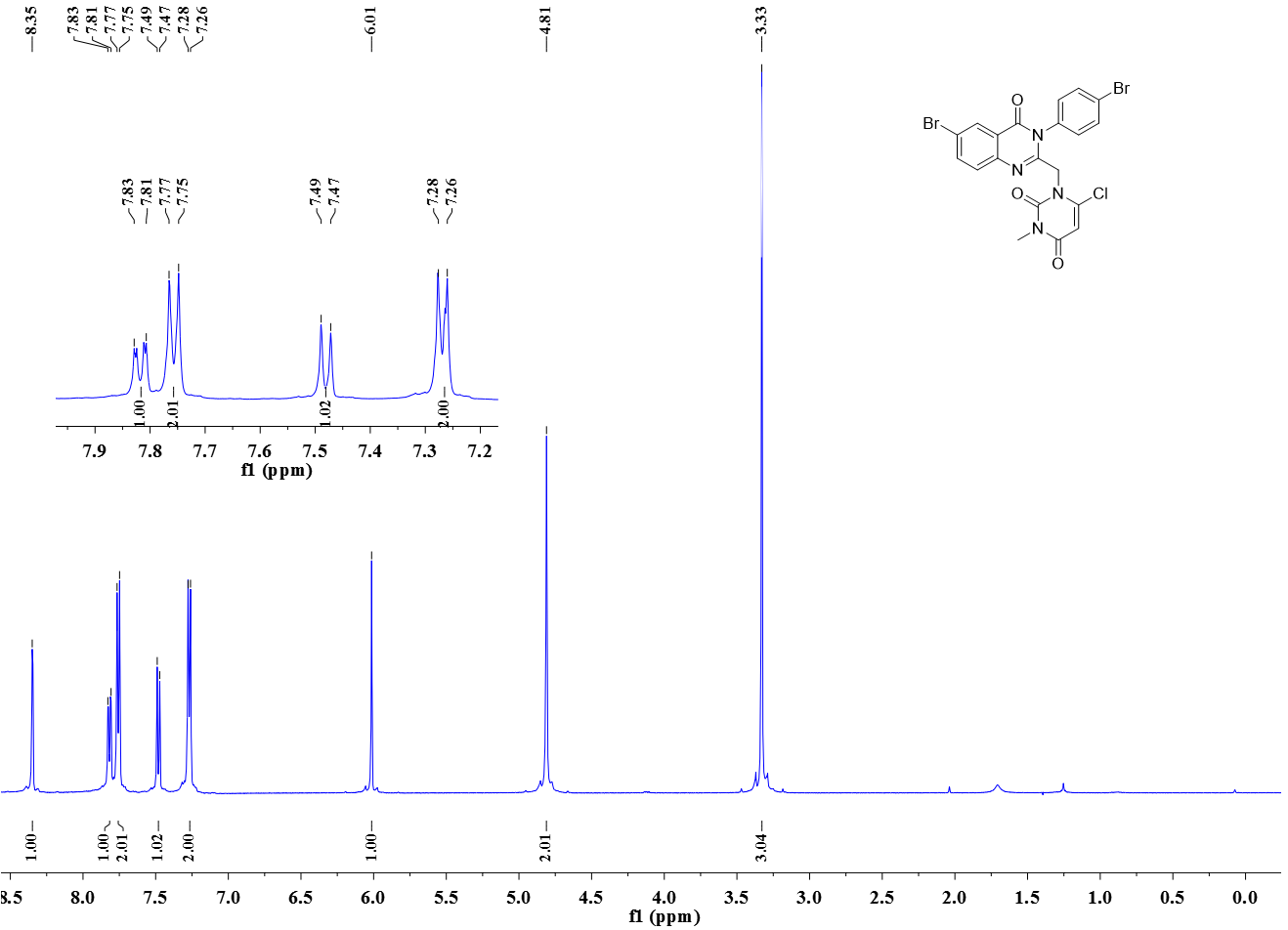


**Figure S24**. ^1^H-NMR spectrum of ***6h***

**
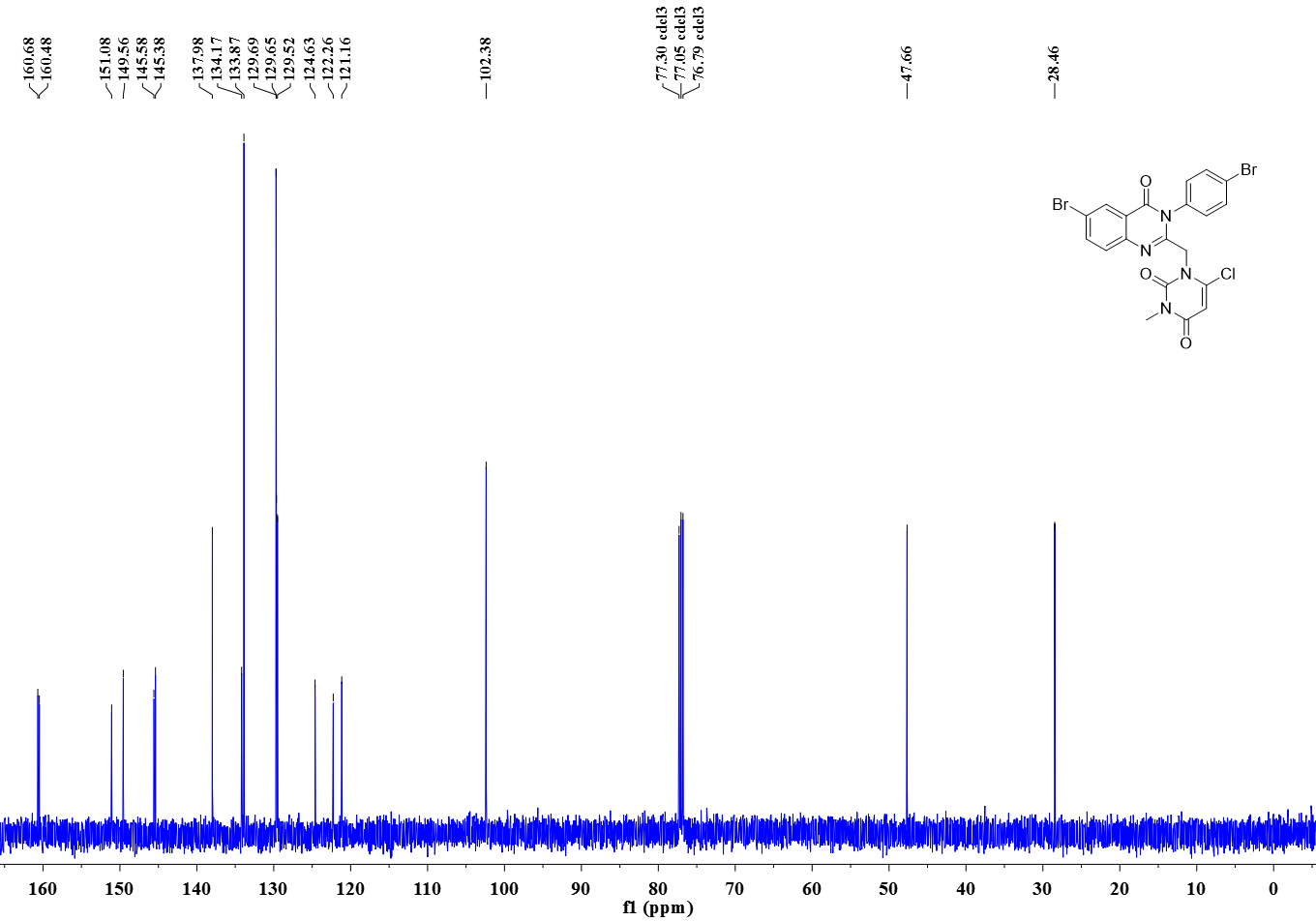
**

**Figure S25**. ^13^C-NMR spectrum of ***6h***


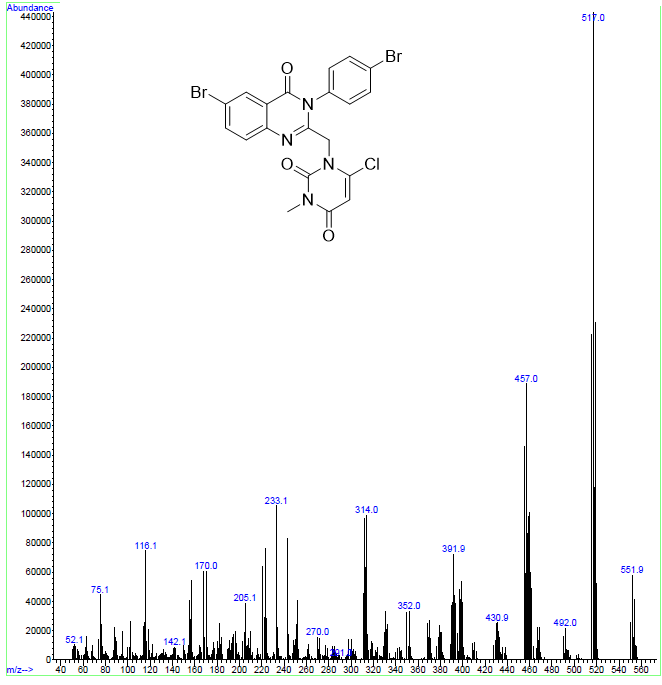


**Figure S26**. Mass spectrum of ***6h***

**
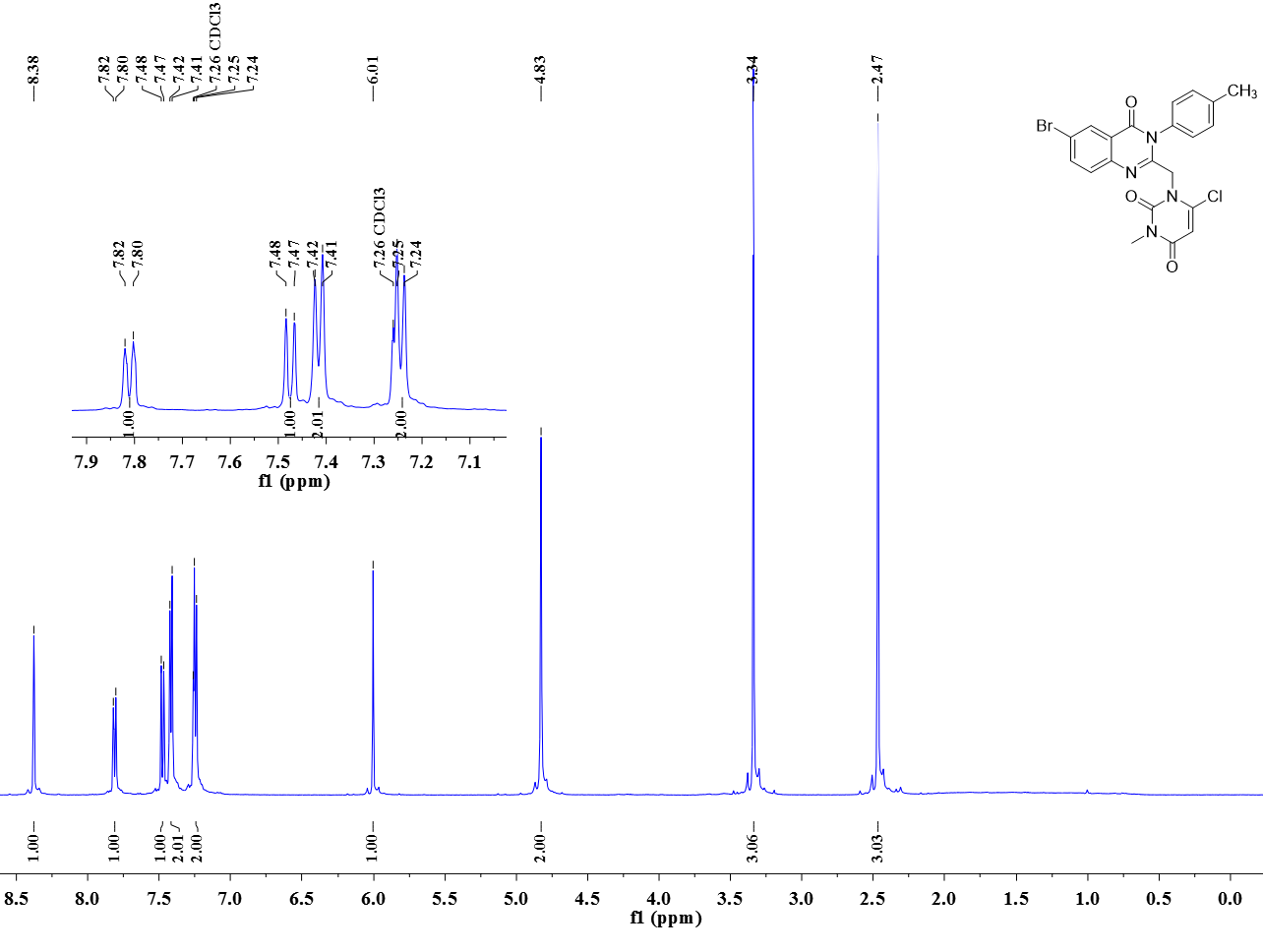
**

**Figure S27**. ^1^H-NMR spectrum of ***6i***

**
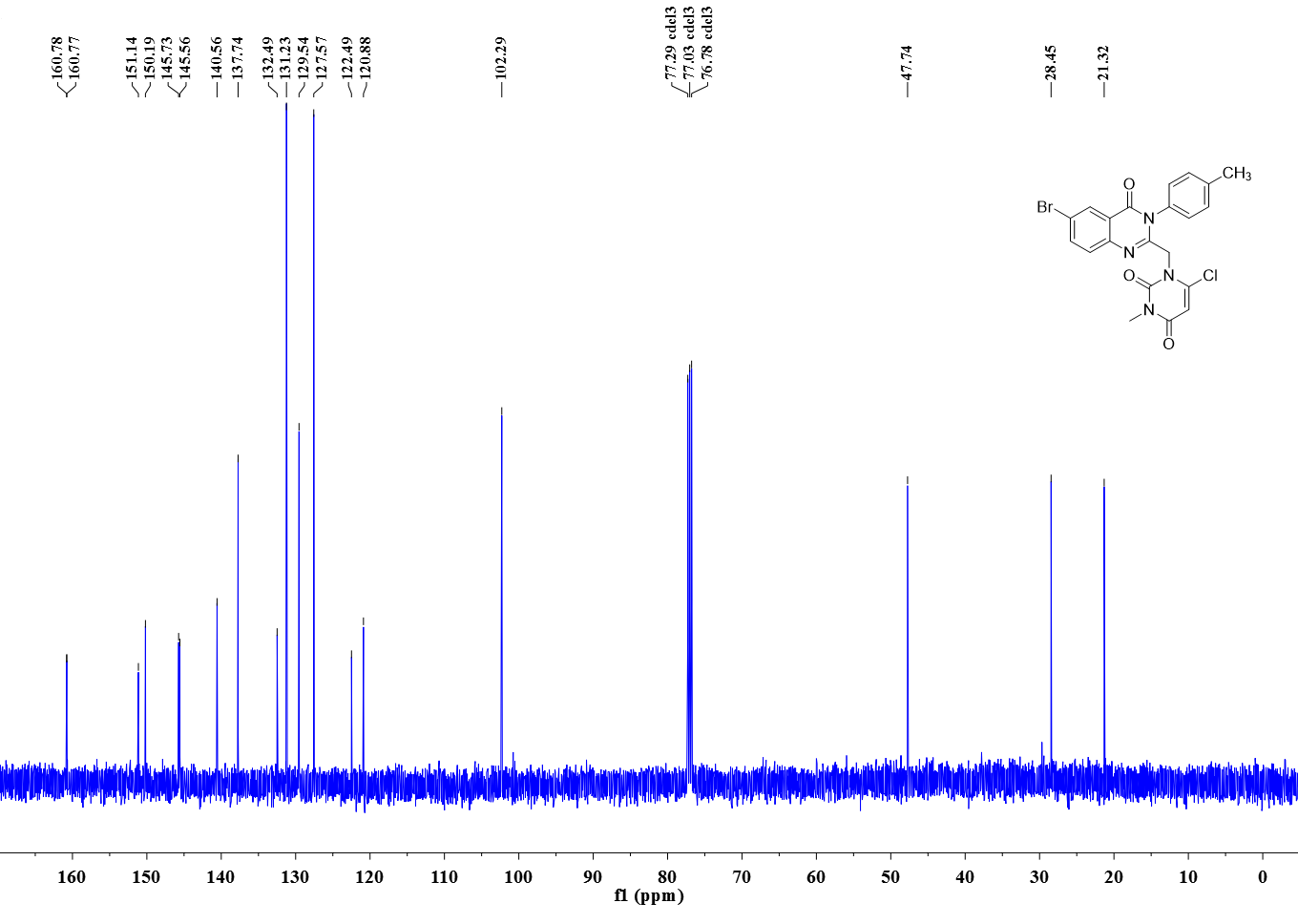
**

**Figure S28**. ^13^C-NMR spectrum of ***6i***

***
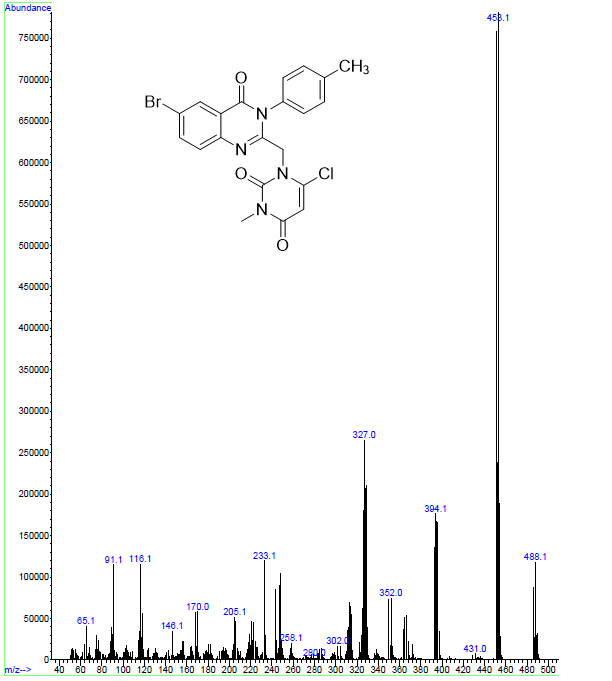
***

**Figure S29**. Mass spectrum of ***6i***

***
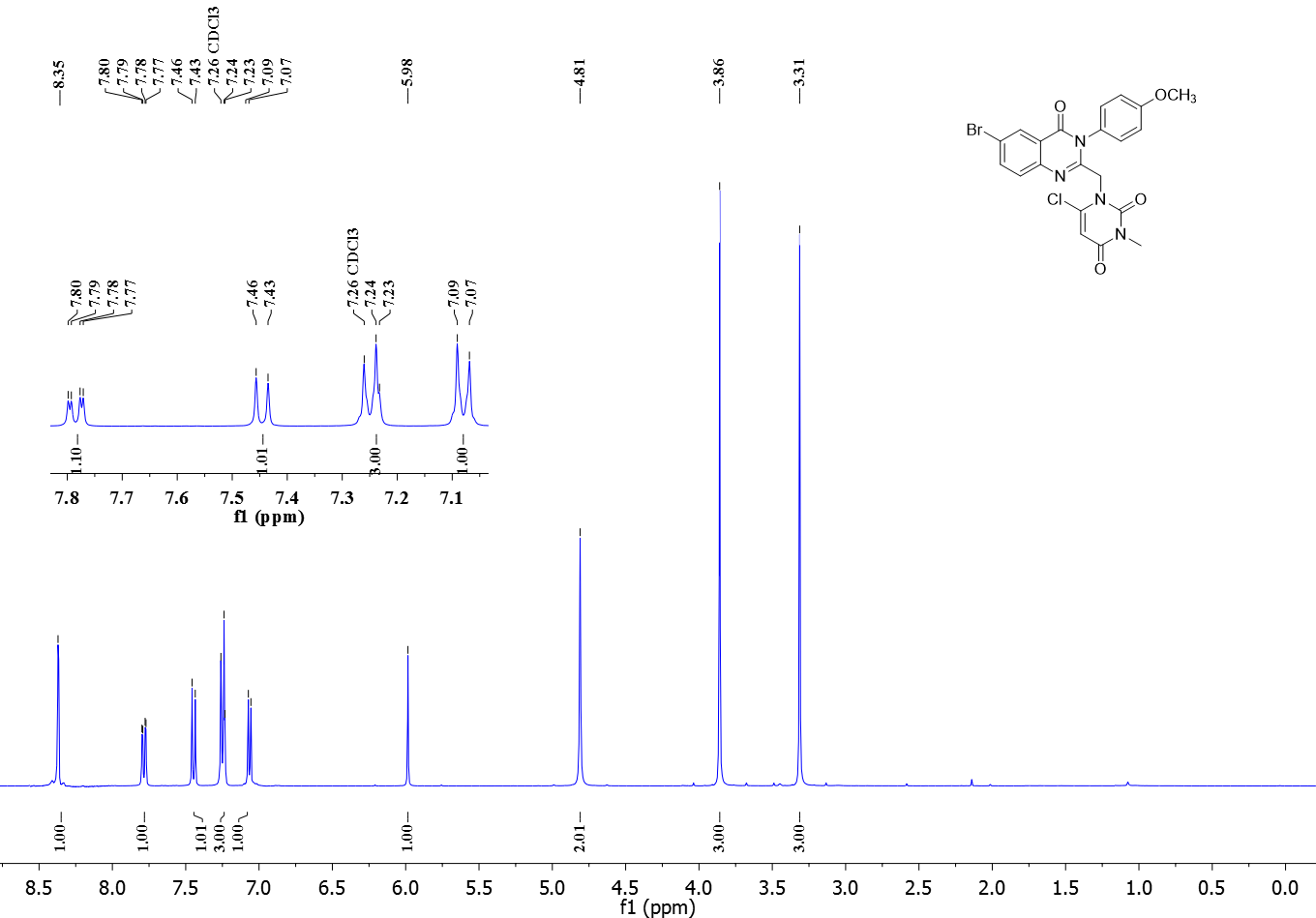
***

**Figure S30**. ^1^H-NMR spectrum of ***6j***

***
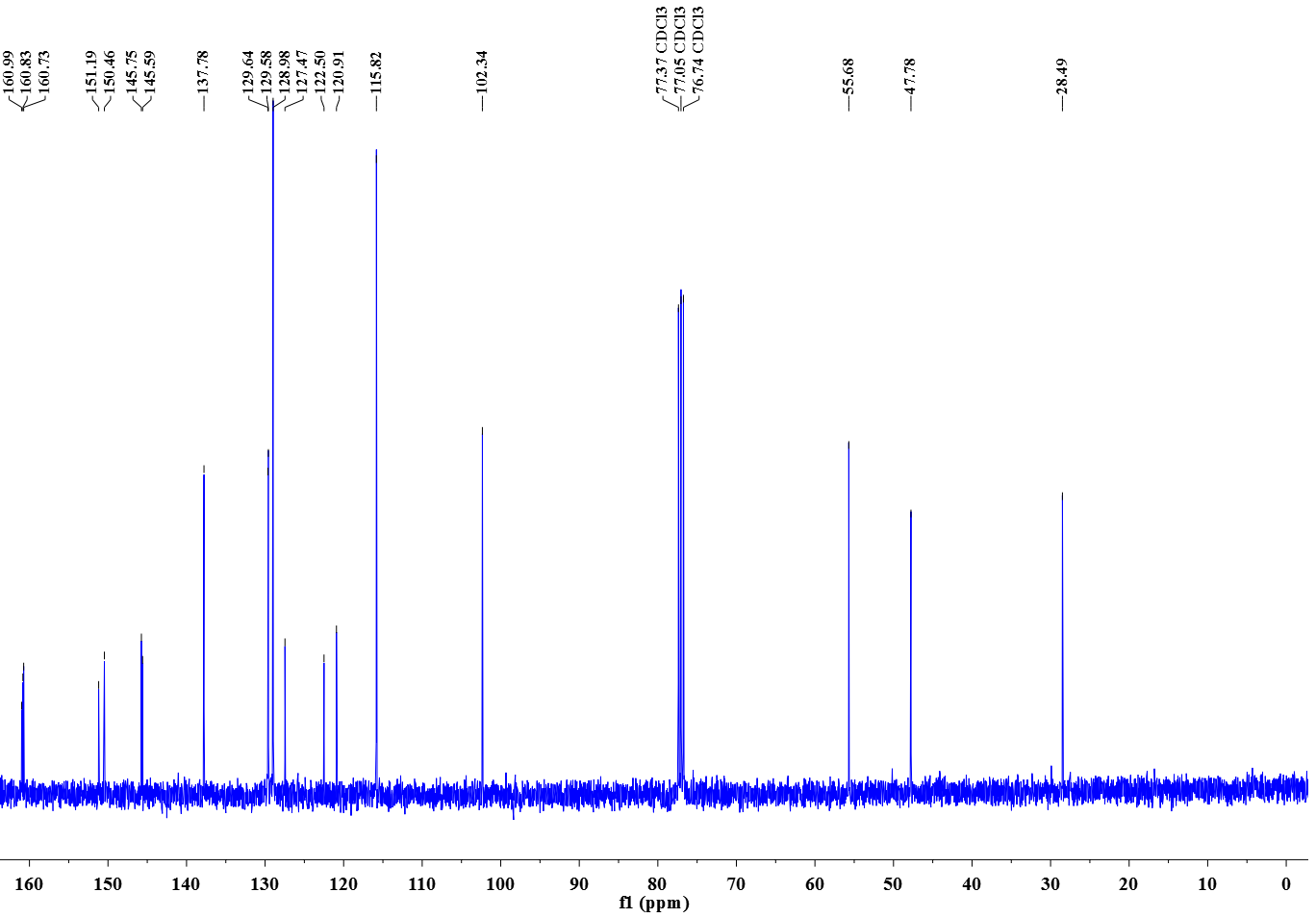
***

**Figure S31**. ^13^C-NMR spectrum of ***6j***


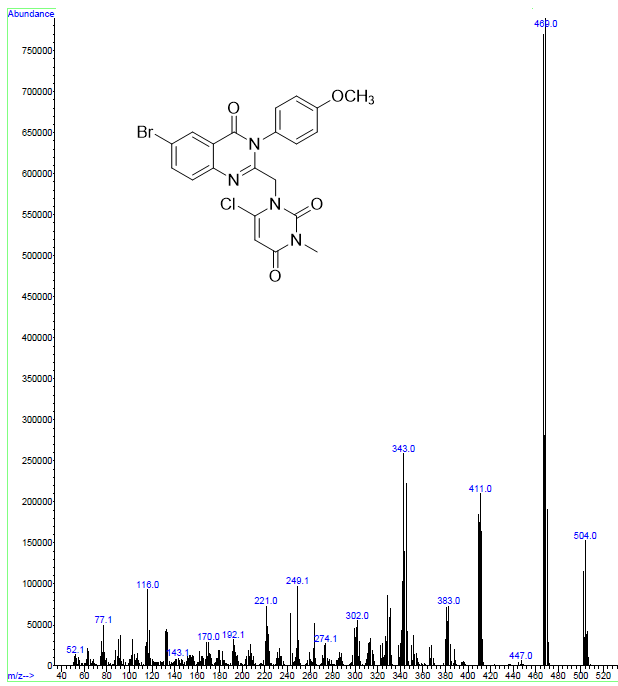


**Figure S32**. Mass spectrum of ***6j***


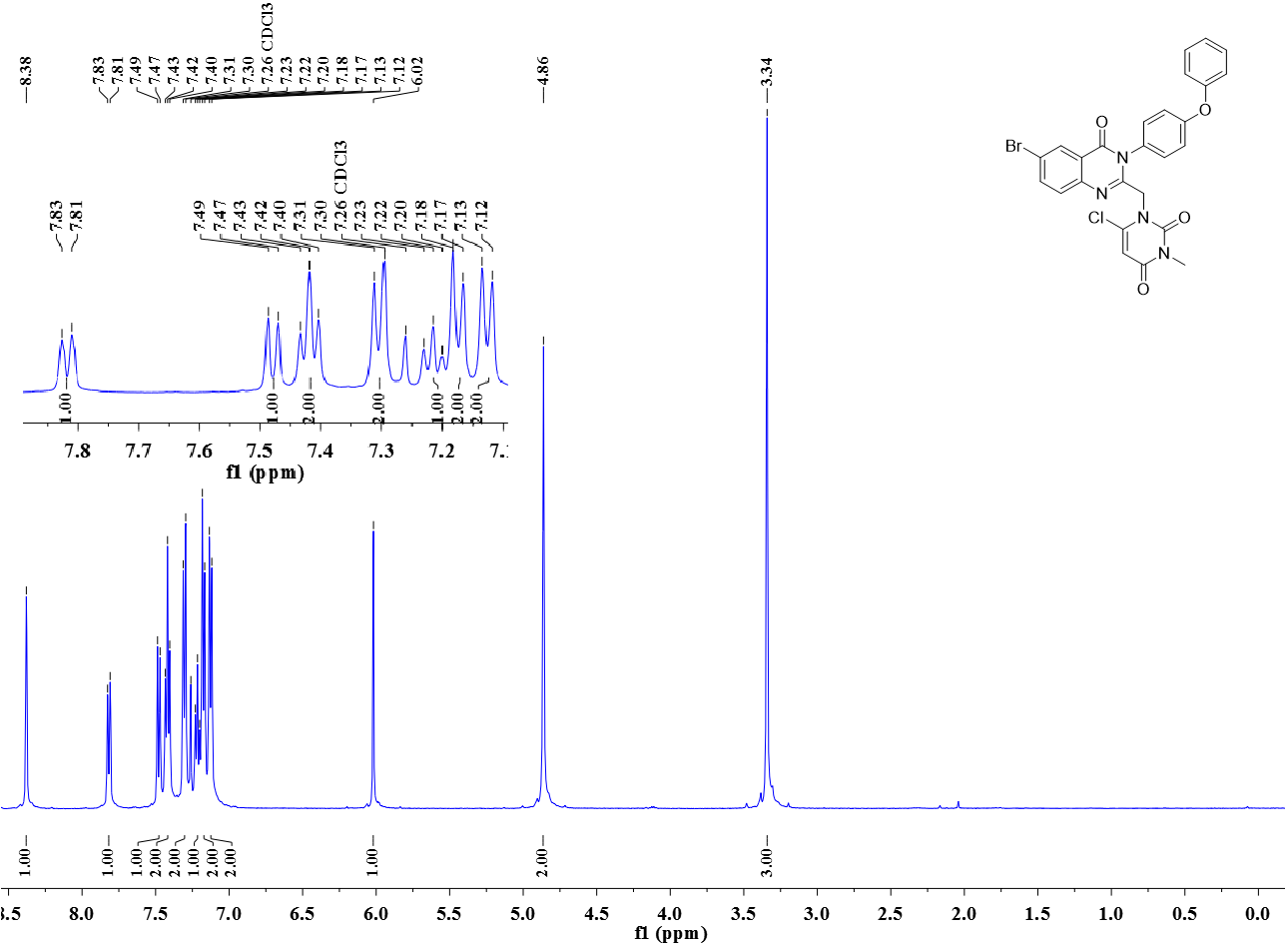


**Figure S33**. ^1^H-NMR spectrum of ***6k***


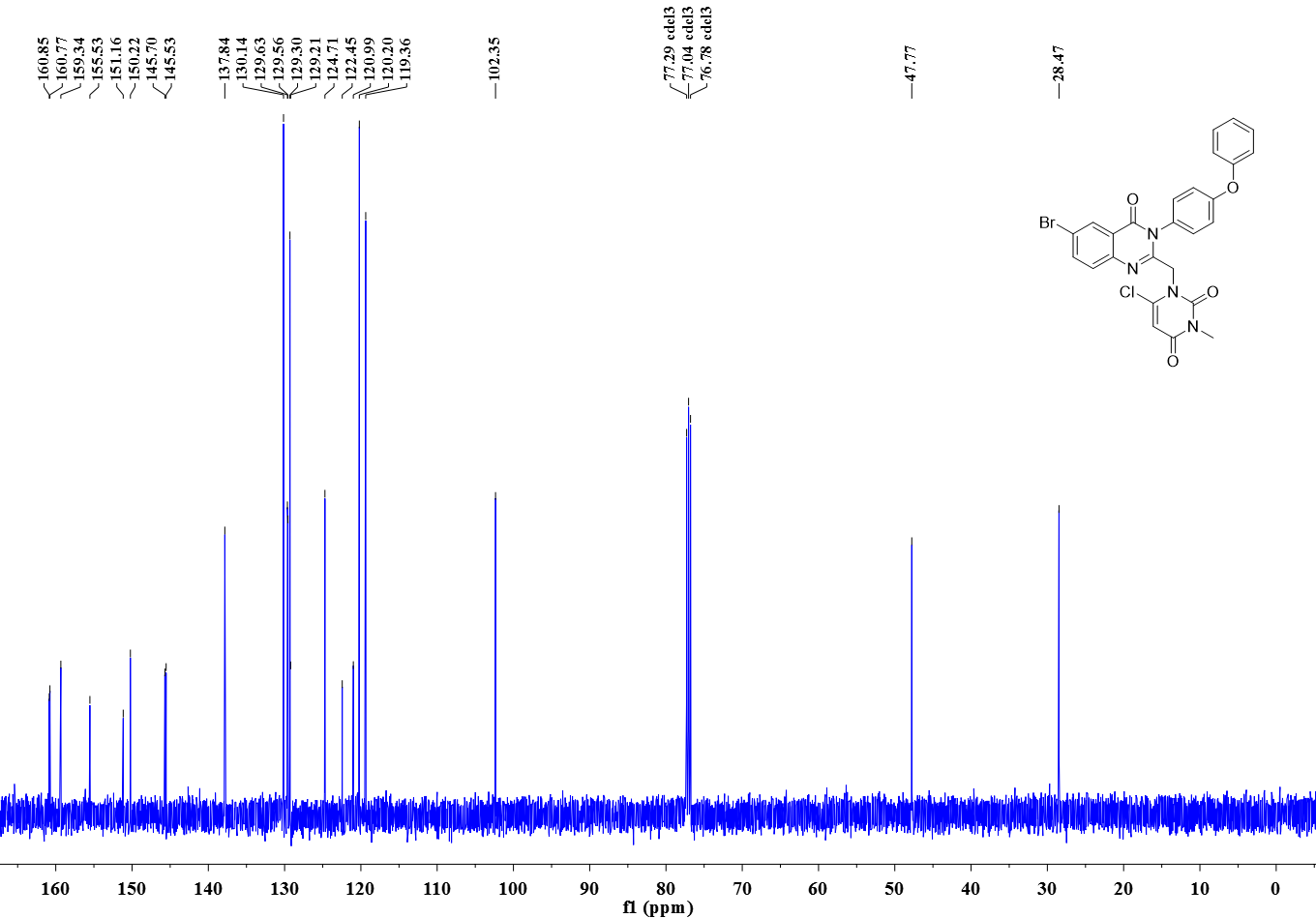


**Figure S34**. ^13^C-NMR spectrum of ***6k***


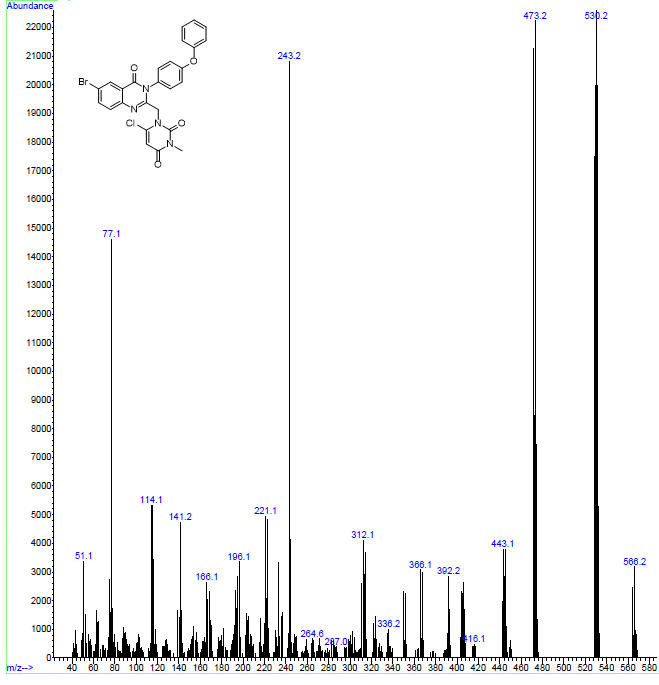


**Figure S35.** Mass spectrum of ***6k***


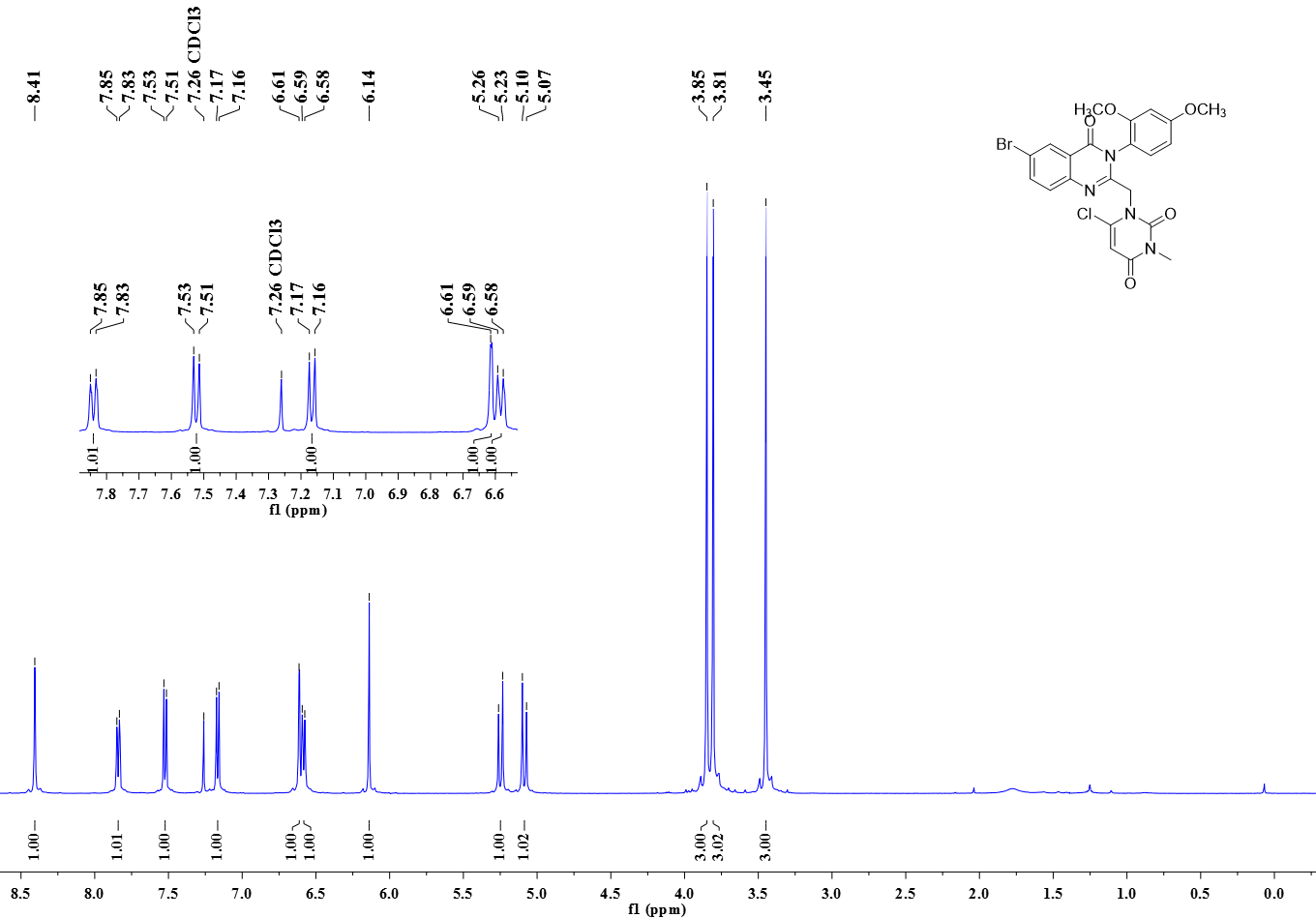


**Figure S36**. ^1^H-NMR spectrum of ***6l***


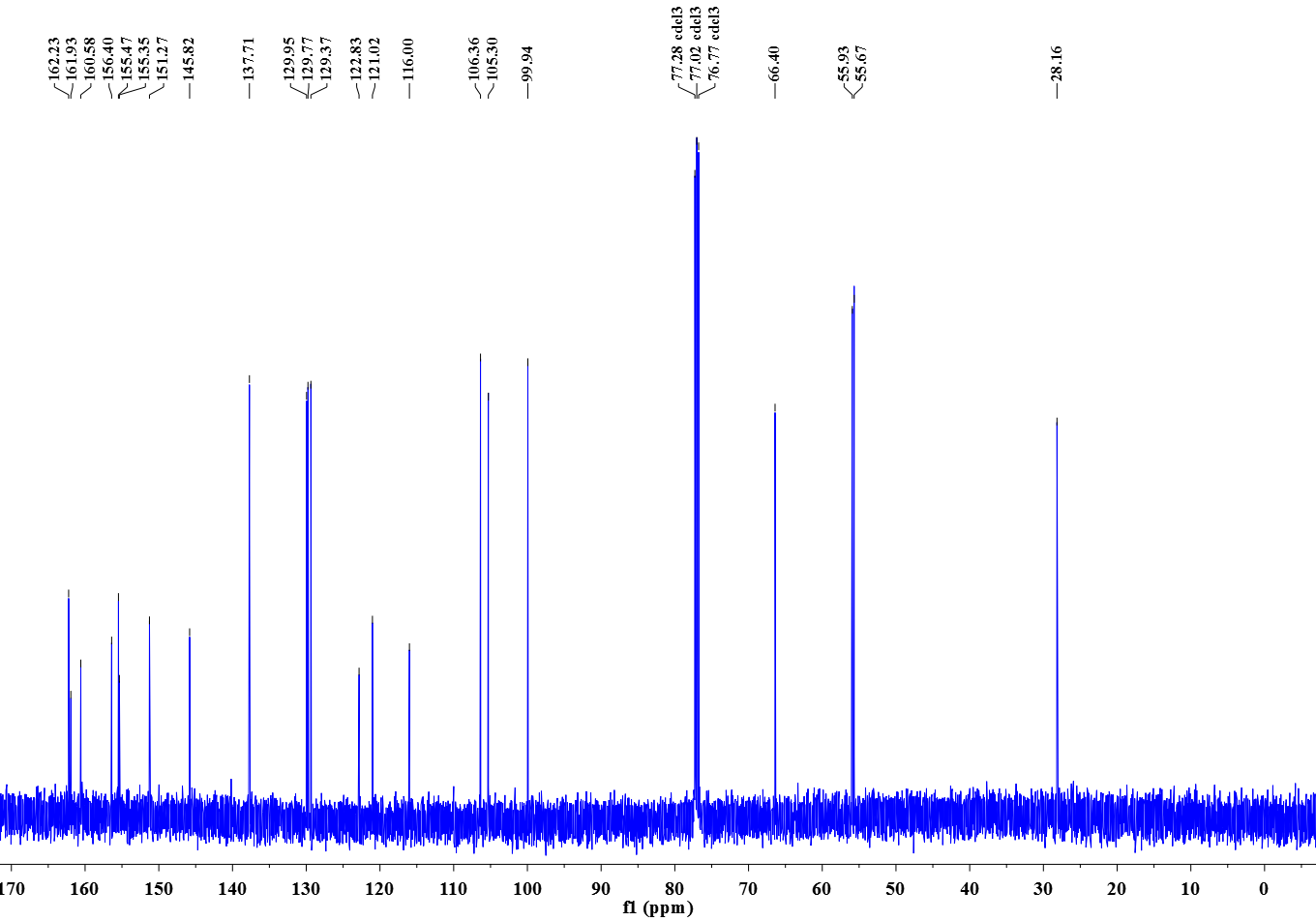


**Figure S37**. ^13^C -NMR spectrum of ***6l***


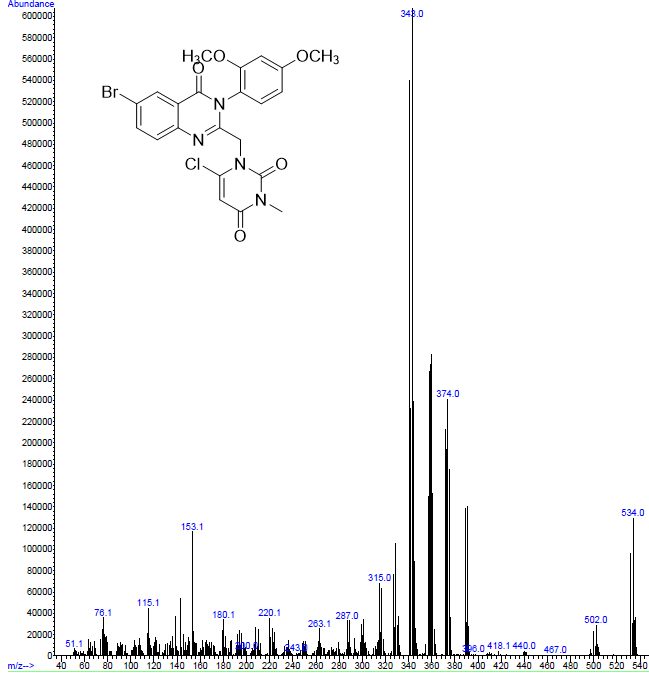


**Figure S38.** Mass spectrum of ***6l***


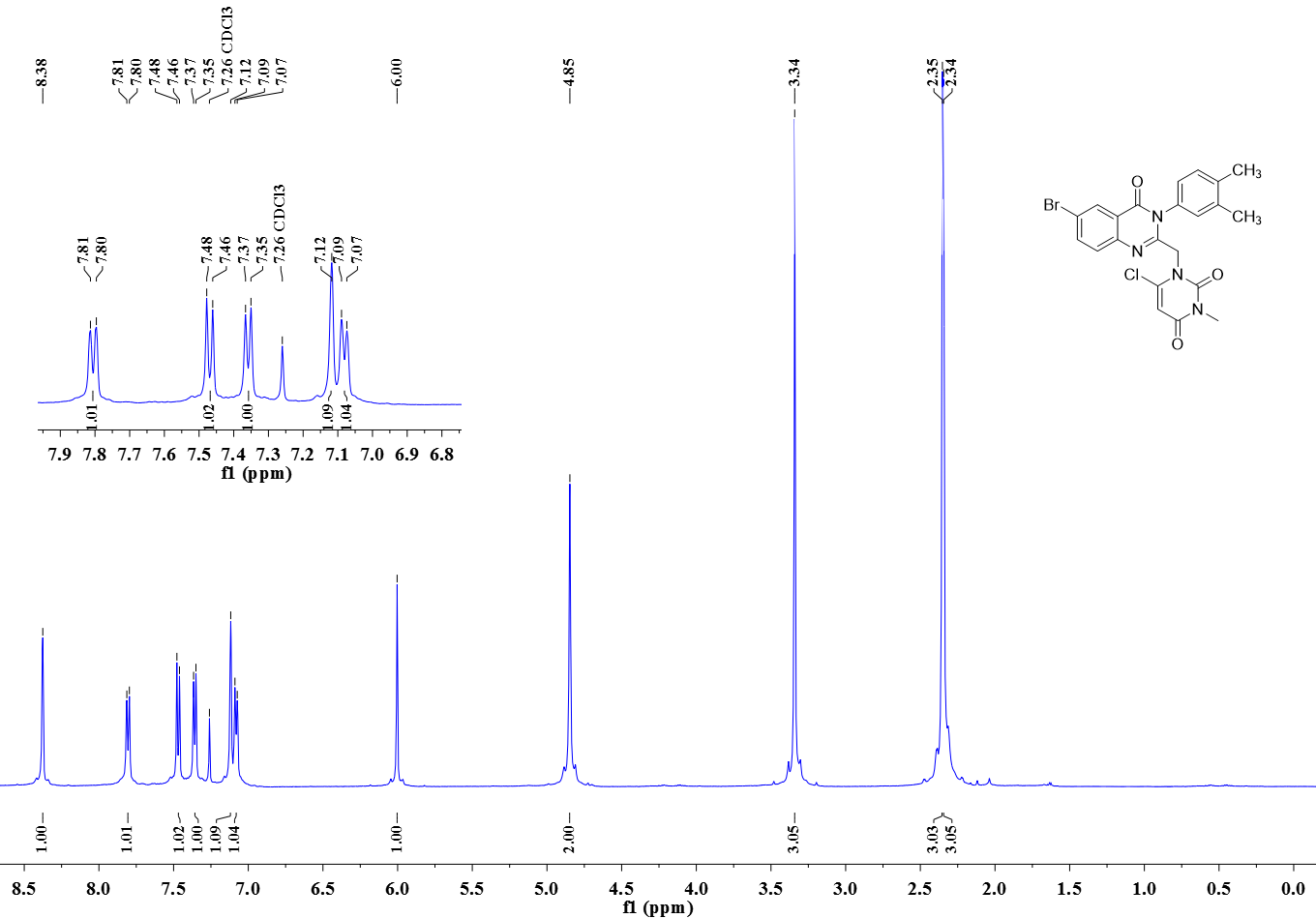


**Figure S39**. ^1^H-NMR spectrum of ***6m***


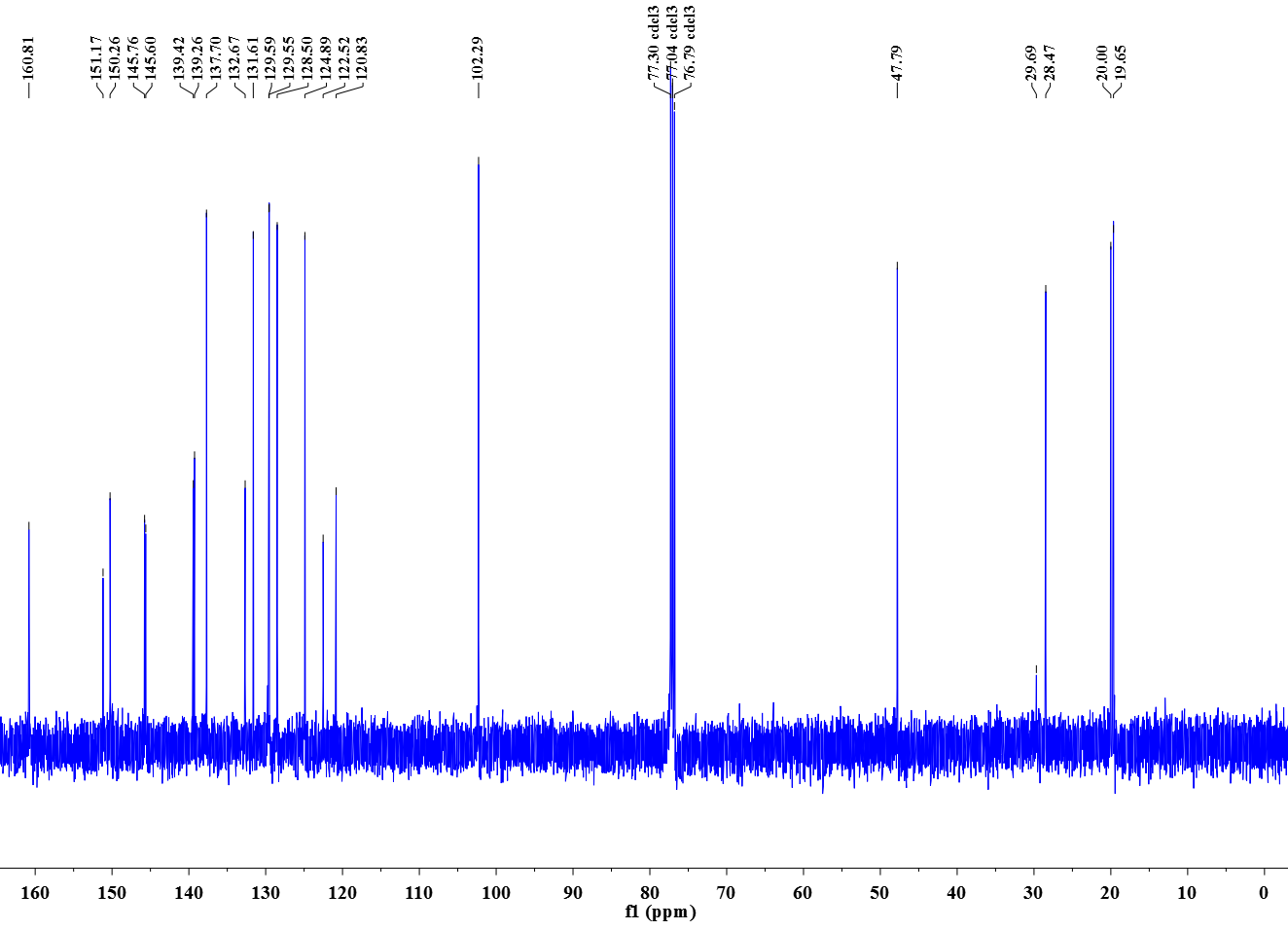
**Figure S34**. ^13^C -NMR spectrum of ***6l***

**Figure S40**. ^13^C -NMR spectrum of ***6m***


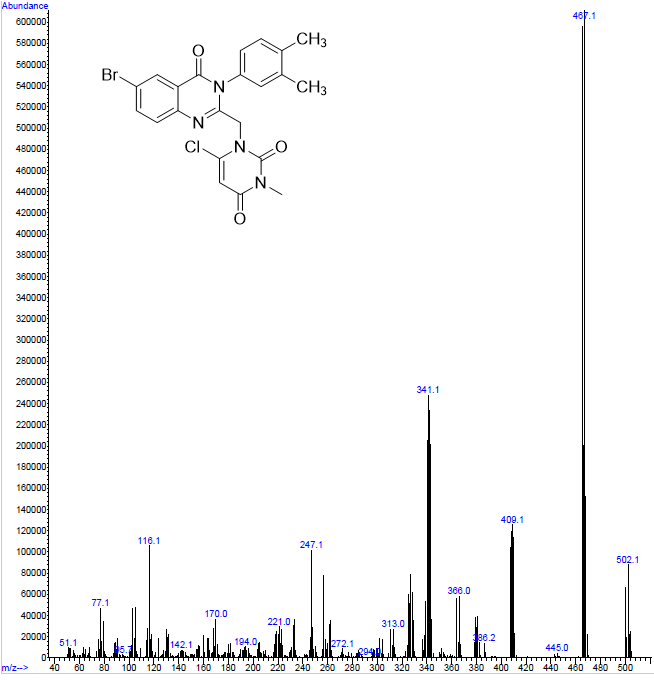


**Figure S41.** Mass spectrum of ***6m***


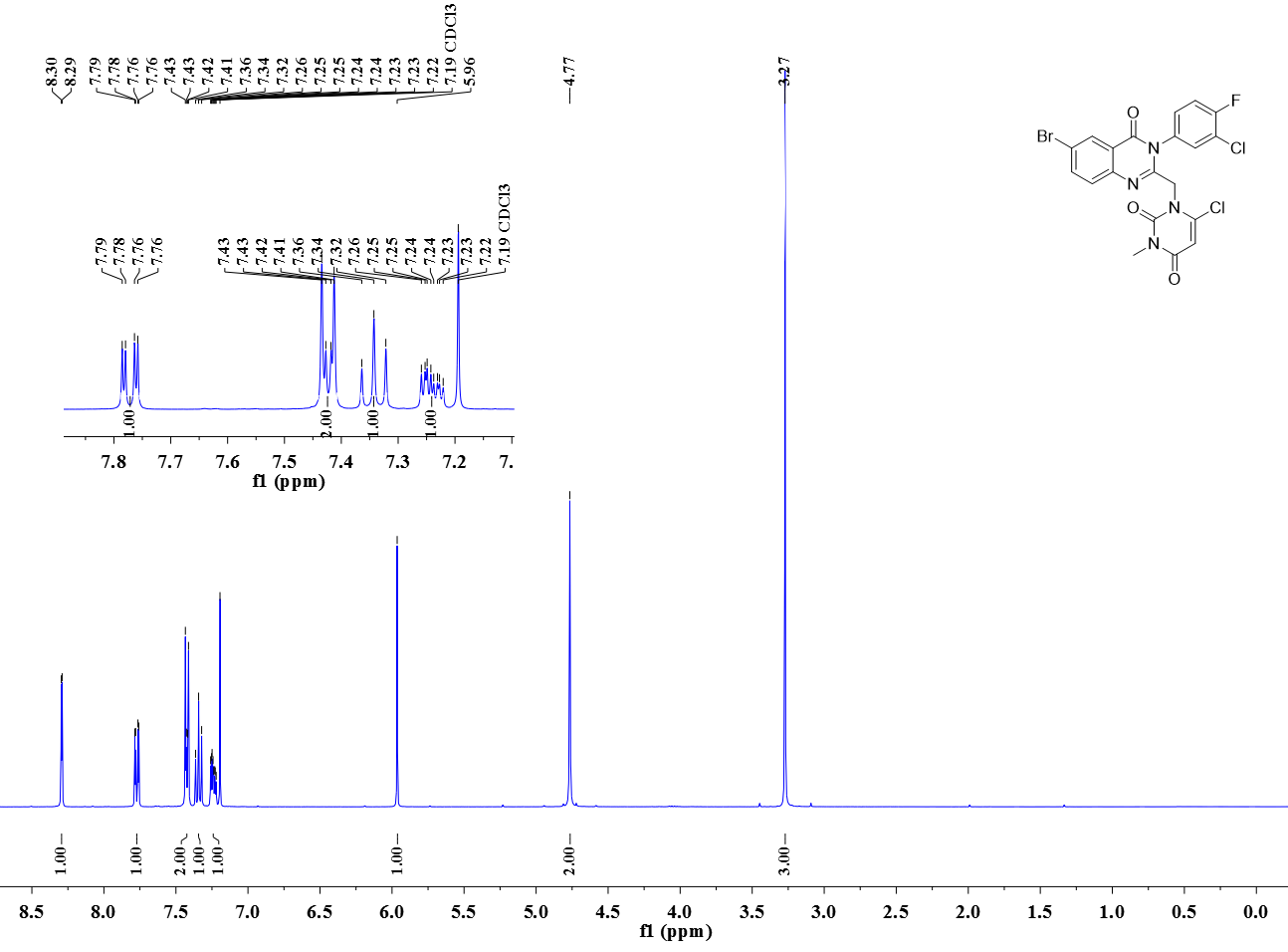


**Figure S42**. ^1^H-NMR spectrum of ***6n***


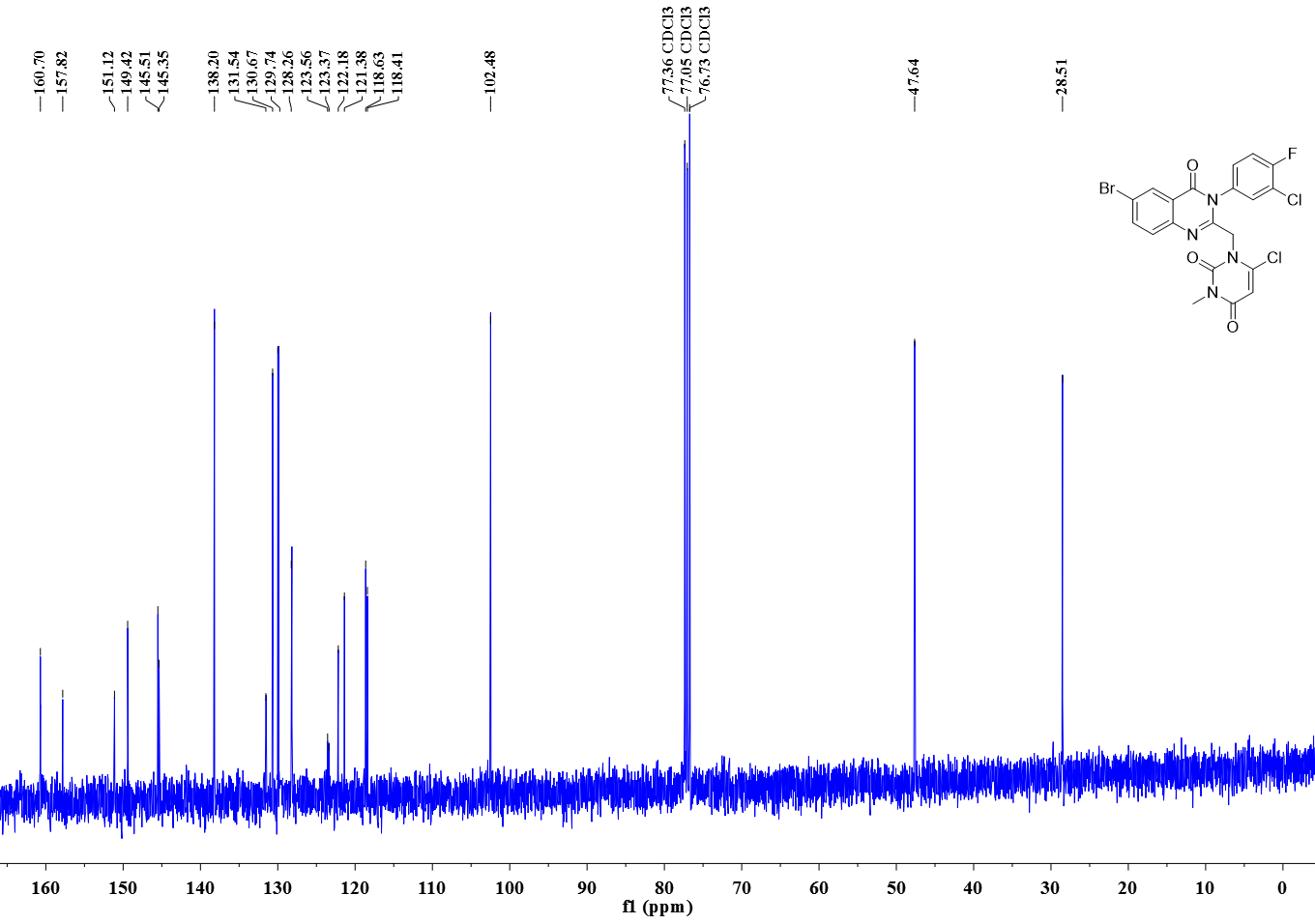


**Figure S43**. ^13^C -NMR spectrum of ***6n***


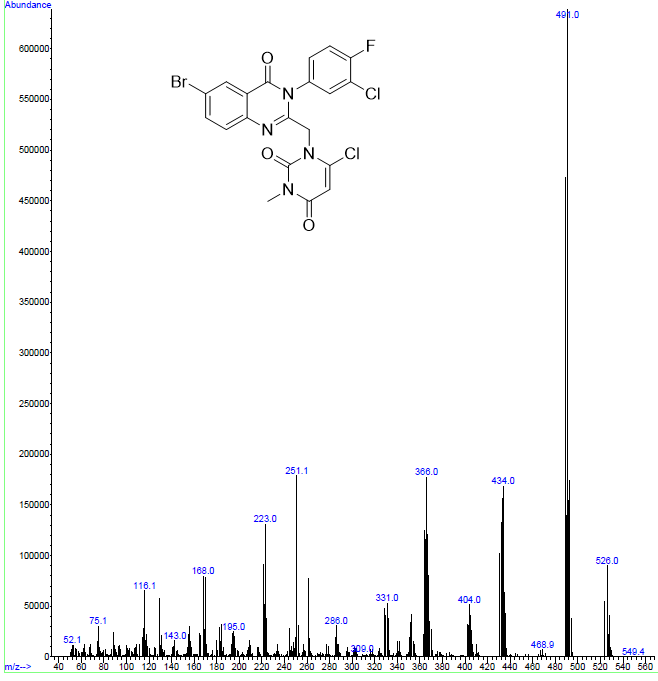


**Figure S44.** Mass spectrum of ***6n***

1. Corresponding Author: Tel: +98-71-32424127-8; Fax: +98-71-32424126; E-mail: [khabns@sums.ac.ir](mailto:khabns@sums.ac.ir). [↑](#footnote-ref-1)
